# The moderating role of trait mindfulness on pain perception: insights from pain-related electroencephalography oscillations

**DOI:** 10.1097/PR9.0000000000001333

**Published:** 2025-10-07

**Authors:** Chen Lu, Nele Berner, Lena Hagel, Nils Jannik Heukamp, Vera Moliadze, Frauke Nees

**Affiliations:** Institute of Medical Psychology and Medical Sociology, University Medical Center Schleswig-Holstein, Kiel University, Kiel, Germany

**Keywords:** EEG oscillation, Moderation model, Pain intensity, Pain perception, Trait mindfulness

## Abstract

**Introduction:**

Pain perception is a subjective experience influenced by individual emotional and cognitive, including attentional and cognitive processes. In this respect, trait mindfulness—as an individual's inherent tendency to remain nonelaborative, nonjudgmental aware and attentive to the present moment—may modulate electroencephalography (EEG) responses to pain stimulation and perceived pain intensity. However, this potential relationship remains largely unexplored in experimental research and was therefore the focus of this study.

**Method:**

Forty healthy participants, aged 20 to 30 years, completed a trait mindfulness questionnaire, followed by experimental thermal pain stimulation while their brain activity was recorded using EEG and pain intensity ratings were assessed during the stimulation (pain perception). Correlation and hierarchical regression analyses were used to explore the relationships between trait mindfulness, EEG responses to pain stimulation, and pain perception.

**Result:**

Trait mindfulness was positively correlated with pain intensity ratings during pain stimulation and δ power in the occipital region. Pain intensity ratings during pain stimulation were negatively correlated with the γ power in the frontal region. Lower θ and α power during pain stimulation predicted higher subsequent pain intensity ratings, but only in individuals with low or moderate trait mindfulness.

**Conclusion:**

This study underscores the moderating role of trait mindfulness in the relationship between frontal, central, parietal, occipital, as well as temporal brain activity and pain perception during pain stimulation. The findings suggest trait mindfulness may be an important factor to consider in clinical pain management and intervention: It can affect cognitive-affective regulation strategies and factors during pain processing.

## Abstract 2

Supplemental Digital Content is Available in the Text.

Trait mindfulness plays a moderating role in the relationship between EEG responses to pain stimulation and pain perception.

## 1. Introduction

Pain is differentially perceived, depending on individual processes at the cognitive and affective levels.^52,58,60^ For example, heightened attention to pain amplifies perceived pain intensity^7^ while being in a positive emotional state mitigates the perceived pain intensity.^32^

Among such attentional and emotion-based processes, the concept of mindfulness has been increasingly discussed. Mindfulness is generally described as a “nonelaborative, nonjudgmental awareness” of present-moment experience,^22^ which is proposed as a facet or personality characteristic based on attentional processes and might thus positively affect the perception of pain.^17,52,57,59,60^ Consistent with this, a reduction of pain intensity in individuals who used mindfulness-based strategies intending to increase the acceptance of pain or reduce the interference of cognitive-affective processes in pain perception (eg, rumination or catastrophizing) was found.^6,9,17,42,53,60^

At the neural level, attentional processes are assumed to be processed in the brain in regions including the anterior cingulate cortex, thalamus, and insula,^28,33^ which are related to perceived pain intensity,^1,45,51^ presumably given its relation to sensory-discriminative and cognitive-affective components of pain. For mindfulness specifically, mindfulness-induced reductions of pain intensity ratings are associated with increased activity in the anterior cingulate cortex and anterior insula^57,59^ and that a higher level of mindfulness is associated with a reduced connectivity of the default mode network,^17^ as well as stronger deactivation of the precuneus to the posterior cingulate cortex during painful stimulation.^60^

Although these findings showed a significant impact of mindfulness on both the neural and psychological level of pain processing, it remains unclear how this is exactly mapped onto the temporal resolution of neural processes, and with mindfulness as a specific psychological factor, and whether and how the interaction relates to pain perception. Regarding this, electroencephalography (EEG), with its superior temporal resolution, offers unique advantages in exploring the interplay of mindfulness and related neural processes in pain processing,^61^ complementing MRI research in this context. Electroencephalography has been used to investigate changes in frequency bands, such as θ, α, and γ oscillations during pain perception, showing a link to various aspects of cognitive and emotional processing during pain experience.^25,47,47,61^ Attention to painful stimuli was found to induce a steady and sustained decrease in α oscillatory power and an increase in γ oscillatory power, both of which are significantly correlated with subjective pain intensity.^41^ Moreover, increased θ oscillations over the prefrontal cortex are associated with stronger emotion regulation through strategies of cognitive reappraisal.^10^ The association between EEG correlates and mindfulness has been found, but still not yet to pain processing. These studies found associations between mindfulness and enhanced α and θ power, which may be seen as the neural correlates of a relaxed alert state.^29^ Whether and how this also affects pain perception or whether these are separate, independent pathways, remains unclear.

Our study thus examined the associations between trait mindfulness, EEG responses to pain stimulation, and pain perception, also considering a potential modulating effect of trait mindfulness on the associations between EEG responses to pain stimulation and pain perception. Based on previous research findings, we primarily focused on θ, α, and γ powers, and investigated associations between EEG powers in other frequency bands, trait mindfulness, and pain perception in an exploratory fashion. Given that sex differences were found in both the levels of trait mindfulness^3,12^ and pain perception,^40,56^ sex was considered as a covariate in analyses.

We hypothesized (1) trait mindfulness is positively correlated to θ and α power during pain processing; (2) pain intensity ratings are negatively correlated to α power and positively correlated to γ power during painful stimulation; and (3) trait mindfulness significantly moderates the association between EEG responses to pain stimulation and pain intensity ratings. Only in individuals with high trait mindfulness, θ, α, and γ power during painful stimulation are assumed to significantly negatively correlate with pain intensity ratings.

## 2. Method

### 2.1. Participants

Fifty-three healthy participants were recruited through flyers and online advertisements. Participants were required to be native German speakers, right-handed, and aged between 20 and 30 years. Exclusion criteria included prior mindfulness-based training experience; any known psychiatric, neurological, or internist disorders; chronic or subacute pain; current or past drug addiction; use of regular medication (excluding hormonal contraception), particularly analgesics or central nervous system-active medications; pregnancy; IQ below 80; or meditation experience (excluding yoga) within the past 8 weeks.

Screening measures for these criteria included pregnancy tests for female participants, assessment of general intelligence using the revised German Culture Fair Intelligence Test 2 (CFT-20-R),^54^ and determination of handedness using the Edinburgh Handedness Inventory.^39^ Additional screenings were conducted primarily through structured interviews and self-reported information provided by participants. All participants were informed about the study and gave written informed consent before the experiment.

Among the 53 participants recruited, we excluded N = 13 given that less than 10 EEG segments^27,36^ were available for data analysis after preprocessing (see 2.2.3.1 EEG signal preprocessing). Finally, 40 participants (mean age = 23.33 ± 1.803 years; 13 male and 27 female participants; see Table 1 for sample characteristics) were included in the analyses.

**Table 1:** Descriptive statistics for sample characteristics.

Variables	M ± SD	Observed range
Sex	Male = 13, female = 27	—
Age	23.33 ± 1.803	20–27
IQ	113.25 ± 15.218	82–142
Mindfulness (MAAS)	3.69 ± 0.747	2.2–4.87
MPIR	77.70 ± 7.838	59.99–90.53
Temperature	46.59 ± 1.514	43.2–49

The temperature refers to the temperature presented as the pain stimulation for participants in the pain perception task.

M, mean; MAAS, Mindful Attention and Awareness Scale; MPIR, mean of pain intensity ratings; SD, standard deviation.

The study was approved by the local ethics committee of the Medical Faculty, Kiel University, Kiel, Germany (AZD482/22) and was conducted in accordance with the latest revision of the Declaration of Helsinki (Clinical Trial Registration Number: German Clinical Trials Register DRKS-ID: DRKS00032271).

### 2.2. Study design

**Figure 1.**
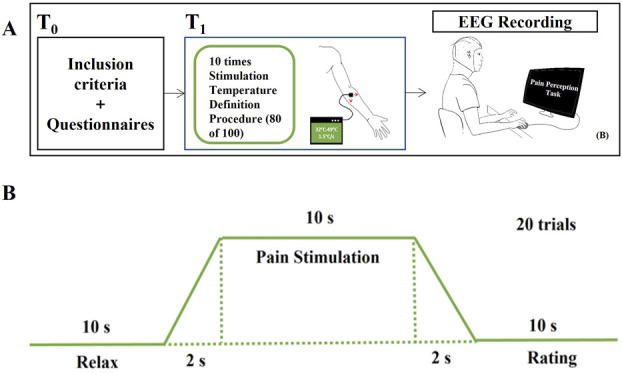
The experimental procedure of this study. (A) An overview of the flow of the study. T0 refers to the participant's first visit, and T1 refers to the participant's second visit. T0 was scheduled within 1 week before T1. (B) The flow of each trial of the pain perception task.

The study design is described in Figure 1.

#### 2.2.1. Assessment of trait mindfulness

We assessed trait mindfulness using the mean of the total scores of the Mindful Attention and Awareness Scale, German Version (MAAS),^34^ with higher scores indicating a higher level of trait mindfulness. The MAAS measures mindfulness as a single factor relating to attention, comprises 15 self-referential statements measured on a 6-point scale (1 = almost always, 6 = almost never). The MAAS was completed online, using the SoSci tool,^26^ before the experimental procedure.

#### 2.2.2. Experimental procedure

The experimental procedure consisted of the stimulus temperature definition procedure and the pain perception task.

##### 2.2.2.1. Stimulation temperature definition

A standardized protocol was implemented to determine the temperature for pain stimulus in the subsequent pain perception tasks for each participant.^44^ Thermal stimuli were delivered using a TSA-2001 thermal stimulator (Medoc Advanced Medical Systems, Haifa, Israel) equipped with a 30 mm × 30-mm flat thermode applied to the ventral surface of the left forearm. The thermode temperature was increased from a baseline of 32°C to a maximum of 49°C at a rate of 1.5°C/second. Participants were instructed to terminate the temperature increase through a button press on reaching a subjective pain intensity of 80 on a verbally communicated 100-point scale, where 0 represented “no pain” and 100 denoted “unbearable pain.” This procedure was repeated 10 times.

The stimulus for the pain perception task was defined as the average temperature recorded during the final 5 trials. If participants did not perceive an 80-level pain intensity at the maximum temperature (49°C), 49°C was designated as the stimulus temperature.

##### 2.2.2.2. Pain perception task

This task comprised 20 consecutive trials. In each trial, the thermode temperature increased from the baseline to the target temperature over 2 seconds, remained at this temperature for 10 seconds before returning to baseline. Then, participants rated pain intensity using a visual analog scale ranging from 0 to 100, which was displayed for 10 seconds. A 10-second relaxation period ensued before the next trial. Participants could use any coping strategy spontaneously during pain stimulation because no specific instructions were given except to minimize movement and blinking to reduce ocular and muscular artifacts.

The mean of pain intensity ratings was calculated as an indicator of pain perception. Given that N = 5 participants missed ratings for 1 to 5 trials, we decided to use the exact number of trials for each participant to calculate the mean of pain intensity ratings, resulting in 15 to 20 trials per participant, which is valid for the pain habituation process.^20,21,50^

##### 2.2.2.3. Electroencephalography recording

Electroencephalography was continuously recorded throughout the pain perception task using 64 sintered Ag/AgCl electrodes mounted on an elastic cap (FMS, Munich, Germany). The electrodes were positioned according to the standard international 10/20 system. The reference and ground electrodes were located between Fp1 and Fp2. In addition, a vertical electrooculogram was recorded beneath the right eye to monitor eye movements during the experiment. Electrode impedances were maintained below 10 kΩ. Electroencephalography data were acquired using two 32-channel BrainAmp amplifiers (Brain Products GmbH, Gilching, Germany) and recorded with BrainVision Recorder Software (Brain Products GmbH). Signals were sampled at 1000 Hz and low-pass filtered at 250 Hz.

#### 2.2.3. Data analysis

##### 2.2.3.1. Electroencephalography signal preprocessing

Electroencephalography signal preprocessing was performed in BrainVisionAnalyzer 2 (Brain Products GmbH). After being down-sampled to 250 Hz, all data were referenced to an average activity of all electrodes (average reference). A fourth-order IIR Butterworth filter was then applied. Data were low-pass filtered at 120 Hz, high-pass filtered at 0.53 Hz, and notch filtered at 50 Hz. Then, the raw data inspection was conducted to identify large artifacts, such as those caused by electrode pops, strong muscle activity, or large movements, which may dominate independent component analysis (ICA) components and reduce the accuracy of separating meaningful neural signals from noise. In this step, signals from the trials without pain stimulation (because of technique issues) will also be marked manually and removed in the step “artifact rejection.” Next, an independent component analysis (ICA, infomax restricted biased) was performed to remove artifacts. Data were segmented according to the markers indicating that the stimulus begins. Each segment contains 500 milliseconds before stimulus onset, a 2-second rise in stimulus temperature, a 10-second platform phase of the target stimulus temperature, and a 2-second fall in stimulus temperature. The segmented data underwent a baseline correlation based on the interval of 500 milliseconds before stimulus onset. Then, artifact rejection was performed automatically before the data were segmented again to make each segment only contain the 10-second platform phase of the target stimulus temperature. Topographic interpolations were conducted before the first-time segmentation, if any bad channels were detected.

A fast Fourier transform (FFT) was performed with a Hanning window (length = 10%). The power spectra (absolute power) were averaged over the segments in every participant. From these average FFTs, we extracted average band power in the δ (0.1–4 Hz), θ (4–8 Hz), α (8–12 Hz), β (12–30 Hz), and γ (30–100 Hz), range for statistical analysis. The EEG channels were grouped according to cortical lobes to facilitate subsequent analyses within each region of interest (ROI). The defined ROIs included the frontal lobe (Fp1, Fp2, Fpz, F1, F2, Fz, F3, F4, F5, F6, F7, F8, AF3, AF4, AF7, AF8), central (FC1, FC2, FC3, FC4, FC5, FC6, Cz, C1, C2, C3, C4, C5, C6, CPz, CP1, CP2, CP3, CP4, CP5, CP6), parietal (Pz, P1, P2, P3, P4, P5, P6, P7, P8, PO3, PO4, PO7, PO8), occipital (O1, O2, Oz, POz), and temporal (FT7, FT8, T7, T8, TP7, TP8, TP9, TP10).

##### 2.2.3.2. Statistical analyses

To investigate the associations among trait mindfulness, EEG responses to pain stimulation (EEG oscillation power of 5 frequency bands in 5 ROIs), and pain intensity ratings, partial correlation analyses were conducted, considering sex as a covariate.

To examine the potential impact of trait mindfulness on the association between EEG responses to pain stimulation and pain intensity ratings, we performed hierarchical regression analyses. The mean pain intensity ratings served as the outcome variable, while EEG power for each frequency band in each ROI was used as the predictor variable. In total, 25 hierarchical regression models were run, each with EEG power from a specific frequency band in a specific brain region as the predictor. In the first step of each model, EEG power and trait mindfulness were entered as predictors. In the second step, sex was included as a covariate to account for its potential influence on the prediction of pain intensity ratings. Finally, in the third step, the interaction term (the product of the EEG power and trait mindfulness) was added to assess the potential moderating effect of trait mindfulness. The underlying assumptions of the regression model (normal distribution, variance homogeneity, autocorrelation, multicollinearity, and case-wise diagnostics for influential outliers, see Supplementary Materials, http://links.lww.com/PR9/A342) were met. A bootstrap resampling procedure with 5,000 samples was used to enhance the robustness of the results. This procedure was applied to the statistical estimation of the partial correlation analysis and all hierarchical regression models. Statistical significance was determined when the 95% bootstrapped confidence interval did not include 0.

Considering that performing correlation analyses and 25 hierarchical regression models between trait mindfulness, pain intensity ratings, and 5 frequency bands in 5 regions, may increase the risk of type I errors (false positives), we applied a false discovery rate (FDR) correction to *P*-values from hypothesis-driven tests (see Supplementary Materials, http://links.lww.com/PR9/A342). Models with EEG power in other frequency bands are treated as exploratory (no further *P*-value correction), and the findings and implications based on these models are referenced accordingly in the Discussion section.

For models where significant interactions were found, we conducted simple slope analyses to examine the relationship between EEG power and pain intensity ratings at lower, moderate, and higher levels of trait mindfulness (ie, M ±1 SD of the moderator). In addition, Johnson-Neyman techniques were used to determine the specific ranges of trait mindfulness within which the effect of EEG power on pain intensity ratings were statistically significant. All continuous variables in hierarchical regression models, simple slope analyses, and the Johnson-Neyman technique were mean-centered to minimize the problem of multicollinearity.^18^

All the above analyses were performed in SPSS 21.0 (IBM Corp., Armonk, NY), including Hayes PROCESS 4.2 macro.^18^ The *P*-value was adjusted using the Benjamini–Hochberg approach^2^ by the p.adjust function of the stats package,^43^ and the Johnson-Neyman plot was created using the ggplot2 package in R.^55^ We applied a *P*-value <0.05 (all tests were 2-sided).

## 3. Results

### 3.1. Correlations between trait mindfulness, electroencephalography responses to pain stimulation, and pain intensity ratings

Trait mindfulness was positively correlated with the pain intensity ratings (r = 0.436, 95% Boot CI [0.144, 0.662]) and occipital δ power (r = 0.405, 95% Boot CI [0.058, 0.656]) when controlled for sex. The pain intensity ratings were negatively correlated to the frontal γ power (r = −0.495, 95% Boot CI [−0.683, −0.185]) when controlled for sex.

### 3.2. The moderating role of trait mindfulness in the relationship between electroencephalography responses to pain stimulation and pain intensity ratings

As summarized in Table 2, we found significant moderating effects of trait mindfulness in the relationship between pain intensity ratings and parietal θ power (b = 36.435, 95% Boot CI [7.209, 73.064], F (4, 35) = 5.169, *P* = 0.002, see Model 8), occipital θ power (b = 33.396, 95% Boot CI [13.569, 82.076], F (4, 35) = 5.145, *P* = 0.002, see Model 9), frontal α power (b = 13.076, 95% Boot CI [4.308, 25.875], F (4, 35) = 5.950, *P* = 0.001, see Model 11), central α power (b = 20.522, 95% Boot CI [9.122, 35.959], F (4, 35) = 5.416, *P* = 0.002, see Model 12), parietal α power (b = 4.169, 95% Boot CI [0.886, 9.042], F (4, 35) = 5.589, *P* = 0.001, see Model 13), occipital α power (b = 3.578, 95% Boot CI [1.114, 6.620], F (4, 35) = 6.403, *P* = 0.001, see Model 14), and temporal α power (b = 18.391, 95% Boot CI [8.433, 29.979], F (4, 35) = 5.804, *P* = 0.001, see Model 15).

**Table 2:** Predicting pain intensity ratings during pain stimulation from trait mindfulness, electroencephalography responses to pain stimulation, and their interactions.

Models	Variables	Step 1	Step 2	Step 3	Simple slope analysis	Johnson-Neyman technique
Model 1	Frontal δ power	−1.885 [−7.281, 2.124]	−3.170 [−7.544, 0.689]	−5.142 [−12.229, −0.295]	—	—
	Trait mindfulness	3.729 [0.252, 6.707]	5.247 [1.811, 8.446]	4.976 [1.630, 8.290]		
	Sex		−6.309 [−11.502, −0.875]	−5.458 [−10.647, −0.268]		
	Frontal δ power × trait mindfulness			5.214 [−0.798, 13.696]		
		F (2, 37) = 2.697, *P* = 0.081	F (3, 36) = 3.987, *P* = 0.015	F (4, 35) = 3.631, *P* = 0.014		
		R^2^ = 0.127	R^2^ = 0.249	R^2^ = 0.293		
Model 2	Central δ power	4.484 [−6.309, 13.551]	2.435 [−8.026, 11.778]	−4.048 [−16.978, 8.019]	—	—
	Trait mindfulness	3.389 [0.046, 6.494]	4.656 [1.237, 7.933]	3.608 [0.330, 6.950]		
	Sex		−5.358 [−10.710, −0.201]	−4.734 [−9.920, 0.182]		
	Central δ power × trait mindfulness			13.514 [−0.869, 33.319]		
		F (2, 37) = 2.814, *P* = 0.073	F (3, 36) = 3.399, *P* = 0.028	F (4, 35) = 3.359, *P* = 0.020		
		R^2^ = 0.132	R^2^ = 0.221	R^2^ = 0.277		
Model 3	Parietal δ power	−0.579 [−4.687, 5.406]	−0.375 [−7.702, 2.534]	−0.238 [−11.136, 3.279]	—	—
	Trait mindfulness	3.575 [0.122, 6.691]	4.804 [1.517, 8.315]	4.773 [1.423, 9.012]		
	Sex		−5.565 [−11.345, −0.169]	−5.066 [−11.559, 1.002]		
	Parietal δ power × trait mindfulness			1.723 [−5.477, 18.430]		
		F (2, 37) = 2.477, *P* = 0.098	F (3, 36) = 3.329, *P* = 0.030	F (4, 35) = 2.473, *P* = 0.062		
		R^2^ = 0.118	R^2^ = 0.217	R^2^ = 0.220		
Model 4	Occipital δ power	1.200 [−3.598, 6.409]	−1.909 [−7.669, 3.802]	−4.575 [−12.665, 1.722]		
	Trait mindfulness	3.389 [−0.056, 6.533]	5.231 [1.671, 8.751]	4.599 [0.665, 8.718]		
	Sex		−6.342 [−12.231, −0.558]	−5.551 [−11.682, −0.053]		
	Occipital δ power × trait mindfulness			6.925 [−2.711, 18.623]		
		F (2, 37) = 2.486, *P* = 0.097	F (3, 36) = 3.450, *P* = 0.027	F (4, 35) = 3.107, *P* = 0.027		
		R^2^ = 0.118	R^2^ = 0.223	R^2^ = 0.262		
Model 5	Temporal δ power	0.205 [−2.118, 4.513]	−0.266 [−2.259, 3.411]	−0.303 [−8.593, 3.633]	—	—
	Trait mindfulness	3.473 [0.082, 6.665]	4.959 [1.423, 8.314]	4.960 [1.444, 9.875]		
	Sex		−5.826 [−11.016, −0.475]	−5.824 [−10.926, −0.209]		
	Temporal δ power × trait mindfulness			0.042 [−2.724, 11.715]		
		F (2, 37) = 2.432, *P* = 0.102	F (3, 36) = 3.345, *P* = 0.030	F (4, 35) = 2.439, *P* = 0.065		
		R^2^ = 0.116	R^2^ = 0.218	R^2^ = 0.218		
Model 6	Frontal θ power	−9.615 [−33.130, 31.426]	−10.092 [−31.523, 26.013]	−14.536 [−41.216, 37.280]	—	—
	Trait mindfulness	3.681 [0.019, 6.735]	4.943 [1.206, 8.101]	4.583 [0.903, 7.873]		
	Sex		−5.654 [−10.378, 0.415]	−4.035 [−9.576, 1.326]		
	Frontal θ power × trait mindfulness			32.229 [−57.455, 72.105]		
		F (2, 37) = 2.800, *P* = 0.074	F (3, 36) = 3.670, *P* = 0.021	F (4, 35) = 3.392, *P* = 0.019		
		R^2^ = 0.131	R^2^ = 0.234	R^2^ = 0.279		
Model 7	Central θ power	−12.287 [−43.008, 40.625]	−12.997 [−40.663, 33.275]	−20.337 [−55.366, 30.837]	—	—
	Trait mindfulness	3.664 [−0.071, 6.696]	4.928 [1.139, 7.997]	4.370 [0.816, 7.690]		
	Sex		−5.665 [−10.387, 0.523]	−3.664 [−8.881, 1.586]		
	Central θ power × trait mindfulness			53.970 [−24.449, 117.501]		
		F (2, 37) = 2.819, *P* = 0.072	F (3, 36) = 3.693, *P* = 0.020	F (4, 35) = 3.994, *P* = 0.009		
		R^2^ = 0.132	R^2^ = 0.235	R^2^ = 0.313		
Model 8	Parietal θ power	−5.949 [−28.804, 10.916]	−7.467 [−27.971, 7.893]	−17.372 [−41.409, −2.172]	Lower levels of trait mindfulness: b = −44.579, *P* = 0.006	Values of trait mindfulness below 0.130
	Trait mindfulness	3.763 [0.335, 6.919]	5.130 [1.546, 8.335]	5.194 [1.972, 8.581]	Moderate levels of trait mindfulness: b = −17.372, *P* = 0.018	
	Sex		−5.916 [−10.585, −0.240]	−3.484 [−8.357, 1.281]	Higher levels of trait mindfulness: b = 9.836, *P* = 0.271	
	Parietal θ power × trait mindfulness			36.435 [7.209, 73.064]		
		F (2, 37) = 2.843, *P* = 0.071	F (3, 36) = 3.884, *P* = 0.017	F (4, 35) = 5.169, *P* = 0.002		
		R^2^ = 0.133	R^2^ = 0.245	R^2^ = 0.371		
Model 9	Occipital θ power	−5.398 [−26.451, 11.992]	−6.831 [−24.616, 8.992]	−17.733 [−43.680, −3.350]	Lower levels of trait mindfulness: b = −42.672, *P* = 0.006	Values of trait mindfulness below 0.137
	Trait mindfulness	3.799 [0.292, 6.917]	5.166 [1.486, 8.397]	5.041 [1.947, 8.816]	Moderate levels of trait mindfulness: b = −17.733, *P* = 0.020	
	Sex		−5.865 [−10.696, 0.026]	−3.680 [−8.597, 1.096]	Higher levels of trait mindfulness: b = 7.205, *P* = 0.372	
	Occipital θ power × trait mindfulness			33.396 [13.569, 82.076]		
		F (2, 37) = 2.747, *P* = 0.077	F (3, 36) = 3.765, *P* = 0.019	F (4, 35) = 5.145, *P* = 0.002		
		R^2^ = 0.129	R^2^ = 0.239	R^2^ = 0.370		
Model 10	Temporal θ power	−7.403 [−31.953, 20.996]	−9.162 [−29.770, 17.007]	−18.230 [−45.550, 12.226]	—	—
	Trait mindfulness	3.785 [0.160, 6.951]	5.132 [1.310, 8.372]	4.663 [1.270, 8.330]		
	Sex		−5.817 [−10.296, 0.103]	−3.882 [−8.819, 1.162]		
	Temporal θ power × trait mindfulness			−31.427 [−6.266, 75.790]		
		F (2, 37) = 2.721, *P* = 0.079	F (3, 36) = 3.712, *P* = 0.020	F (4, 35) = 4.011, *P* = 0.009		
		R^2^ = 0.128	R^2^ = 0.236	R^2^ = 0.314		
Model 11	Frontal α power	−6.465 [−11.952, 3.103]	−5.836 [−10.911, 2.020]	−8.051 [−13.736, −0.732]	Lower levels of trait mindfulness: b = −17.815, *P* = 0.002	Values of trait mindfulness below 0.182
	Trait mindfulness	3.980 [0.386, 7.145]	5.075 [1.494, 8.224]	4.899 [1.754, 8.042]	Moderate levels of trait mindfulness: b = −8.051, *P* = 0.009	
	Sex		−5.115 [−9.675, −0.087]	−3.809 [−8.370, 0.658]	Higher levels of trait mindfulness: b = 1.713, *P* = 0.674	
	Frontal α power × trait mindfulness			13.076 [4.308, 25.875]		
		F (2, 37) = 4.826, *P* = 0.014	F (3, 36) = 4.904, *P* = 0.006	F (4, 35) = 5.950, *P* = 0.001		
		R^2^ = 0.207	R^2^ = 0.290	R^2^ = 0.405		
Model 12	Central α power	−5.733 [−15.518, 6.835]	−4.750 [−13.719, 6.198]	−8.778 [−20.124, 0.825]	Lower levels of trait mindfulness: b = −24.102, *P* = 0.004	Values of trait mindfulness below 0.008
	Trait mindfulness	3.816 [0.354, 7.190]	4.949 [1.412, 8.282]	4.789 [1.582, 7.777]	Moderate levels of trait mindfulness: b = −8.778, *P* = 0.047	
	Sex		−5.303 [−9.880, 0.007]	−4.436 [−9.062, 0.270]	Higher levels of trait mindfulness: b = 6.546, *P* = 0.255	
	Central α power × trait mindfulness			20.522 [9.122, 35.959]		
		F (2, 37) = 3.295, *P* = 0.048	F (3, 36) = 3.800, *P* = 0.018	F (4, 35) = 5.416, *P* = 0.002		
		R^2^ = 0.151	R^2^ = 0.241	R^2^ = 0.382		
Model 13	Parietal α power	−2.590 [−4.672, 0.692]	−2.464 [−4.327, 0.199]	−2.707 [−4.816, −0.230]	Lower levels of trait mindfulness: b = −5.820, *P* = 0.004	Values of trait mindfulness below 0.124
	Trait mindfulness	3.663 [0.362, 6.733]	4.841 [1.445, 7.997]	4.726 [1.633, 7.802]	Moderate levels of trait mindfulness: b = −2.707, *P* = 0.017	
	Sex		−5.329 [−10.098, −0.397]	−4.129 [−8.768, 0.818]	Higher levels of trait mindfulness: b = 0.406, *P* = 0.813	
	Parietal α power × trait mindfulness			4.169 [0.886, 9.042]		
		F (2, 37) = 5.133, *P* = 0.011	F (3, 36) = 5.348, *P* = 0.004	F (4, 35) = 5.589, *P* = 0.001		
		R^2^ = 0.217	R^2^ = 0.308	R^2^ = 0.390		
Model 14	Occipital α power	−1.853 [−3.408, 0.678]	−1.680 [−3.186, 0.327]	−2.423 [−3.942, −0.721]	Lower levels of trait mindfulness: b = −5.095, *P* = 0.001	Values of trait mindfulness below 0.257
	Trait mindfulness	3.927 [0.480, 6.994]	5.017 [1.507, 8.126]	4.506 [1.504, 7.724]	Moderate levels of trait mindfulness: b = −2.423, *P* = 0.004	
	Sex		−5.060 [−9.756, 0.138]	−3.245 [−7.921, 1.427]	Higher levels of trait mindfulness: b = 0.248, *P* = 0.810	
	Occipital α power × trait mindfulness			3.578 [1.114, 6.620]		
		F (2, 37) = 5.211, *P* = 0.010	F (3, 36) = 5.172, *P* = 0.004	F (4, 35) = 6.403, *P* = 0.001		
		R^2^ = 0.220	R^2^ = 0.301	R^2^ = 0.423		
Model 15	Temporal α power	−4.262 [−13.573, 5.437]	−4.060 [−12.026, 4.654]	−9.060 [−17.781, −0.716]	Lower levels of trait mindfulness: b = −22.793, *P* = 0.003	Values of trait mindfulness below 0.083
	Trait mindfulness	3.890 [0.318, 6.969]	5.106 [1.534, 8.186]	4.662 [1.452, 7.448]	Moderate levels of trait mindfulness: b = −9.060, *P* = 0.026	
	Sex		−5.543 [−9.975, −0.272]	−4.615 [−8.873, −0.167]	Higher levels of trait mindfulness: b = 4.673, *P* = 0.311	
	Temporal α power × trait mindfulness			18.391 [8.433, 29.979]		
		F (2, 37) = 3.008, *P* = 0.062	F (3, 36) = 3.759, *P* = 0.019	F (4, 35) = 5.804, *P* = 0.001		
		R^2^ = 0.140	R^2^ = 0.239	R^2^ = 0.399		
Model 16	Frontal β power	−53.992 [−110.174, 39.058]	−86.245 [−176.762, −27.037]	−82.526 [−192.353, 9.132]	—	—
	Trait mindfulness	3.337 [0.027, 6.040]	5.000 [1.803, 7.671]	4.871 [1.467, 7.655]		
	Sex		−8.078 [−13.036, −2.823]	−7.787 [−13.072, −2.193]		
	Frontal β power × trait mindfulness			14.520 [−127.079, 138.166]		
		F (2, 37) = 4.118, *P* = 0.024	F (3, 36) = 6.983, *P* = 0.001	F (4, 35) = 5.133, *P* = 0.002		
		R^2^ = 0.182	R^2^ = 0.368	R^2^ = 0.370		
Model 17	Central β power	11.320 [−173.589, 172.685]	−30.157 [−192.242, 142.722]	−27.683 [−249.949, 139.846]	—	—
	Trait mindfulness	3.547 [0.294, 6.801]	4.891 [1.476, 8.327]	4.945 [1.435, 8.302]		
	Sex		−5.896 [−11.061, −0.328]	−5.963 [−11.223, −0.216]		
	Central β power × trait mindfulness			−11.098 [−228.403, 339.831]		
		F (2, 37) = 2.413, *P* = 0.104	F (3, 36) = 3.376, *P* = 0.029	F (4, 35) = 2.466, *P* = 0.063		
		R^2^ = 0.115	R^2^ = 0.220	R^2^ = 0.220		
Model 18	Parietal β power	9.261 [−33.593, 87.531]	−7.692 [−52.289, 69.227]	3.112 [−79.448, 75.683]	—	—
	Trait mindfulness	3.488 [0.254, 6.847]	4.930 [1.511, 8.400]	5.034 [1.445, 8.486]		
	Sex		−5.929 [−11.091, 0.376]	−5.986 [−11.450, 0.509]		
	Parietal β power × trait mindfulness			−16.378 [−105.919, 108.038]		
		F (2, 37) = 2.495, *P* = 0.096	F (3, 36) = 3.351, *P* = 0.029	F (4, 35) = 2.509, *P* = 0.059		
		R^2^ = 0.119	R^2^ = 0.218	R^2^ = 0.223		
Model 19	Occipital β power	4.840 [−24.873, 43.642]	−4.462 [−30.189, 33.717]	−0.056 [−49.817, 45.677]	—	—
	Trait mindfulness	3.464 [−0.010, 6.607]	4.949 [1.421, 8.190]	4.978 [1.268, 8.196]		
	Sex		−5.886 [−11.092, 0.174]	−5.933 [−11.309, 0.340]		
	Occipital β power × trait mindfulness			−6.516 [−63.469, 81.156]		
		F (2, 37) = 2.470, *P* = 0.098	F (3, 36) = 3.348, *P* = 0.030	F (4, 35) = 2.465, *P* = 0.063		
		R^2^ = 0.118	R^2^ = 0.218	R^2^ = 0.220		
Model 20	Temporal β power	−48.602 [−164.085, 48.864]	−71.445 [−165.596, 26.198]	−85.257 [−190.778, 37.224]	—	—
	Trait mindfulness	3.875 [0.354, 7.150]	5.473 [1.833, 8.928]	4.950 [1.309, 8.304]		
	Sex		−6.523 [−11.340, −1.025]	−5.971 [−10.901, −0.553]		
	Temporal β power × trait mindfulness			49.027 [−120.381, 172.362]		
		F (2, 37) = 3.078, *P* = 0.058	F (3, 36) = 4.513, *P* = 0.009	F (4, 35) = 3.549, *P* = 0.016		
		R^2^ = 0.143	R^2^ = 0.273	R^2^ = 0.289		
Model 21	Frontal γ power	−208.468 [−386.505, −5.351]	−282.165 [−509.877, −170.782]	−254.556 [−483.326, −28.501]	—	—
	Trait mindfulness	2.785 [−0.322, 5.606]	4.244 [1.422, 6.943]	4.238 [1.472, 7.049]		
	Sex		−7.796 [−13.373, −2.838]	−7.484 [−13.289, −2.270]		
	Frontal γ power × trait mindfulness			69.956 [−237.135, 409.738]		
		F (2, 37) = 5.511, *P* = 0.008	F (3, 36) = 8.359, *P* < 0.001	F (4, 35) = 6.201, *P* = 0.001		
		R^2^ = 0.230	R^2^ = 0.411	R^2^ = 0.415		
Model 22	Central γ power	−274.153 [−697.385, 162.454]	−216.016 [−716.366, 327.608]	−168.969 [−776.343, 530.416]	—	—
	Trait mindfulness	3.544 [0.348, 6.525]	4.727 [1.419, 7.985]	5.089 [1.209, 9.030]		
	Sex		−5.316 [−11.049, 0.253]	−5.271 [−11.330, 0.248]		
	Central γ power × trait mindfulness			407.111 [−892.420, 1606.242]		
		F (2, 37) = 3.125, *P* = 0.056	F (3, 36) = 3.666, *P* = 0.021	F (4, 35) = 3.031, *P* = 0.030		
		R^2^ = 0.145	R^2^ = 0.234	R^2^ = 0.257		
Model 23	Parietal γ power	103.571 [−631.042, 352.749]	28.800 [−658.753, 256.286]	−20.398 [−649.496, 574.993]		
	Trait mindfulness	3.520 [0.164, 6.715]	4.769 [1.305, 7.966]	5.163 [0.272, 8.878]	—	—
	Sex		−5.505 [−10.628, −0.189]	−5.357 [−10.664, −0.109]		
	Parietal γ power × trait mindfulness			254.880 [−1091.012, 1092.852]		
		F (2, 37) = 2.612, *P* = 0.087	F (3, 36) = 3.316, *P* = 0.031	F (4, 35) = 2.543, *P* = 0.057		
		R^2^ = 0.124	R^2^ = 0.216	R^2^ = 0.225		
Model 24	Occipital γ power	23.165 [−181.790, 126.246]	11.946 [−166.210, 103.699]	−17.716 [−181.272, 166.267]	—	—
	Trait mindfulness	3.484 [0.078, 6.569]	4.733 [1.249, 7.963]	5.269 [0.381, 9.307]		
	Sex		−5.467 [−10.494, −0.139]	−5.376 [−10.391, −0.106]		
	Occipital γ power × trait mindfulness			75.251 [−303.010, 347.328]		
		F (2, 37) = 2.633, *P* = 0.085	F (3, 36) = 3.353, *P* = 0.029	F (4, 35) = 2.558, *P* = 0.056		
		R^2^ = 0.125	R^2^ = 0.218	R^2^ = 0.226		
Model 25	Temporal γ power	−82.136 [−333.489, 72.630]	−85.401 [−325.757, 73.812]	−112.360 [−382.279, 73.227]	—	—
	Trait mindfulness	3.745 [0.376, 6.714]	5.011 [1.318, 8.494]	4.591 [1.027, 8.344]		
	Sex		−5.668 [−11.541, −0.029]	−4.831 [−10.833, 0.840]		
	Temporal γ power × trait mindfulness			116.861 [−114.106, 334.687]		
		F (2, 37) = 3.063, *P* = 0.059	F (3, 36) = 3.900, *P* = 0.016	F (4, 35) = 3.482, *P* = 0.017		
		R^2^ = 0.142	R^2^ = 0.245	R^2^ = 0.285		

The response variable in all hierarchical regression models in Table 2 is the mean of pain intensity ratings. Trait mindfulness was assessed using the Mindful Attention and Awareness Scale (MAAS). All continuous independent variables were centered (original values minus mean) before being entered into the models. In the first step, the predictors (EEG power in a certain frequency band in a certain ROI, and Trait Mindfulness) are added, in the second step, sex (female = 0, male = 1) is added as a covariate, and in the third step, the product term of the 2 predictors is added. Outside the square brackets are the nonstandardized regression coefficient B, and inside the square brackets are the upper and lower limits of the 95% percentile confidence interval after 5000 Bootstrap samples. For those models where a significant moderation effect of trait mindfulness was found, the simple slope analyses were conducted to examine the relationship between EEG power and pain intensity ratings during pain stimulation at higher and lower, moderate, and higher levels of trait mindfulness (ie, 1 standard deviation above and values of trait mindfulness below the mean of the moderator, the mean of the moderator, and 1 standard deviation above the mean of the moderator). In addition, the Johnson-Neyman technique was used to determine the specific ranges of trait mindfulness within which the effect of EEG power on pain intensity ratings during pain stimulation was statistically significant.

EEG, electroencephalography; ROI, region of interest.

The results of the simple slope analysis and Johnson-Neyman technique for the significant models are summarized in Table 2 and visualized in Figure 2.

Figure 2.Simple slope analysis and Johnson-Neyman plots of the conditional effect of EEG responses to pain stimulation on pain intensity ratings during pain stimulation across levels of trait mindfulness. All continuous independent variables were centered (original values minus mean) before being entered into the models and the units for each variable were 1 point. The relationship between EEG responses and pain intensity ratings during pain stimulation was examined on 3 levels of trait mindfulness and levels of trait mindfulness were defined by 1 standard deviation below the mean, mean, and 1 standard deviation above the mean. For (A–G), the grey shaded area indicates the confidence interval (CI), and the light blue shaded area indicates the significance area. (A) The predictor was θ power in the parietal region. (B) The predictor was θ power in the occipital region. (C) The predictor was α power in the frontal region. (D) The predictor was α power in the central region. (E) The predictor was α power in the parietal region. (F) The predictor was α power in the occipital region. (G) The predictor was α power in the temporal region. For all these models, the moderator was trait mindfulness and the response variable was the mean of pain intensity ratings.

**Figure:**
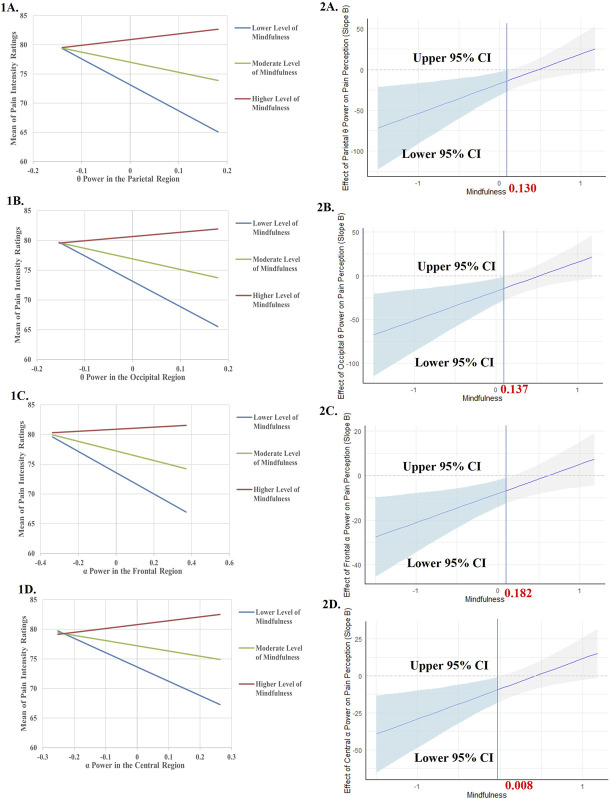

**Figure:**
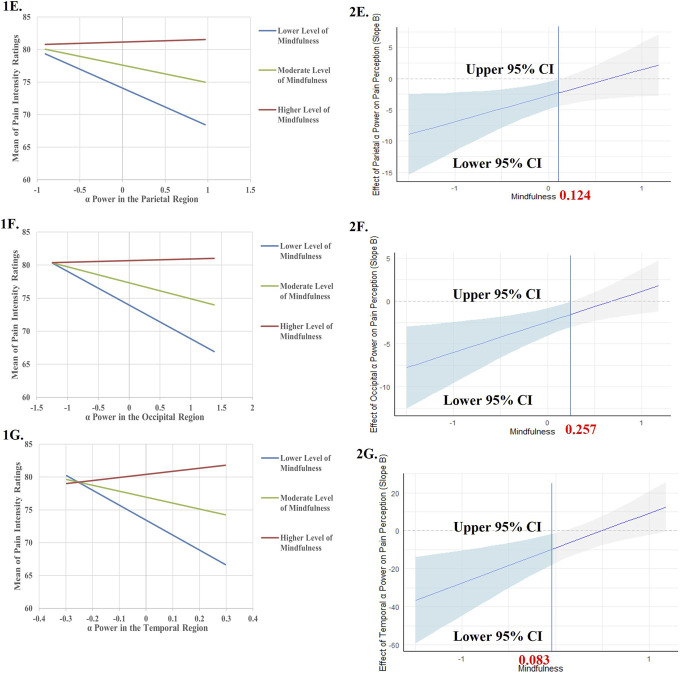

Lower θ power in the parietal or occipital region, and lower α power in the frontal, central, parietal, occipital, or temporal region were found to predict higher pain intensity ratings only when the level of trait mindfulness was low or moderate.

## 4. Discussion

Our study aimed to investigate the associations between trait mindfulness, brain oscillation responses to pain stimulation, and pain perception. We found that (1) trait mindfulness was positively correlated with pain intensity ratings and occipital δ power. (2) Pain intensity ratings were negatively correlated with the frontal γ power, which was not significantly correlated with trait mindfulness. (3) Lower θ power (in the parietal or occipital region) and lower α power (in the frontal, central, parietal (only before FDR correction), occipital, or temporal region) were found to predict higher pain intensity ratings only when trait mindfulness was low or moderate.

Decreased θ and α power during pain stimulation and their correlation with increased perceived pain intensity have also been reported in previous studies,^19,20,38^ which our findings were partly in line with. We also found that these correlations did not apply to individuals with high trait mindfulness. This suggests besides the factors well-known as age and sex, trait mindfulness could be an indispensable member of the individual differences in pain perception and thus is worthy of attention in pain management and intervention.^8,11,35^ For individuals with high trait mindfulness, it may be more appropriate to enhance their mindful attitude when constantly aware of pain than using distraction-based strategies in pain management.

Moreover, the negative correlation between θ and α power and the pain intensity ratings could provide clues for pain modulation mechanisms. Because θ activity in the mid-frontal region and occipital region was found to be related to distraction and may also reflect cognitive reappraisal-based emotion regulation,^10,49^ and α activity reflects top-down adjustments of cognitive control,^5,37^ our findings may suggest an association between less cognitive-affective regulation (eg, distraction) during pain stimulation and higher perceived pain intensity. However, this does not apply to individuals with high trait mindfulness.

A possible explanation is that individuals with low and moderate trait mindfulness may rely on strategies based on cognitive-affective regulation to reduce the pain intensity. Therefore, less cognitive-affective regulation during pain stimulation is linked to higher pain intensity. However, individuals with high trait mindfulness may not rely on those strategies, instead, they may tend to maintain attention to pain stimulation with less interference from cognitive-affective factors.^17,30,60^ This process may relate to a lower pain unpleasantness^13,31,57,59,60^; however, it does not imply a lower pain intensity,^13,31^ which was suggested by our finding as well.

In this regard, while the associations between higher levels of mindfulness and lower pain unpleasantness ratings are often reported, there are mixed findings regarding whether higher levels of mindfulness are associated with lower pain intensity ratings.^13,31,57,59,60^ These mixed findings may reflect multiple mechanisms through which mindfulness influences pain perception, potentially linked to differences in mindfulness assessments across studies, such as novices with short-term mindfulness-based training,^57,59^ experts with long-term mindfulness practice,^13,31^ and trait mindfulness.^60^ Therefore, our finding of a positive correlation between trait mindfulness and pain intensity ratings may suggest how mindfulness, as an individual's inherent tendency, affects pain perception. Specifically, trait mindfulness is associated with higher body awareness,^15,48^ which may link to a continuous awareness and attention to pain during stimulation and, in turn, less habituation to pain stimulation^14^ and thus a higher mean of pain intensity ratings. Particularly, our study assessed trait mindfulness by the MAAS (primarily indexes the attention aspect of mindfulness), which could further explain why individuals with higher scores reported higher pain intensity (greater attention to the present sensation).

Moreover, the subjective pain perception during pain stimulation was found to be encoded by frontal γ oscillations,^46^ and frontal γ oscillations were linked to cognitive-affective regulation based on cognitive reappraisal,^23^ eg, distraction.^4,24^ Therefore, the negative correlations between γ power in the frontal region and pain intensity ratings we found may reflect a top-down modulation of pain perception (eg, distraction); however, this was not associated with trait mindfulness in our data. This may support our assumption that individuals with a high trait mindfulness do not rely on cognitive-affective regulation for pain stimulation.

As a result of exploratory research, we found that trait mindfulness was positively correlated with occipital δ power during pain stimulation, which was different from our hypotheses built on the previous studies.^29^ This may be attributed to what we assessed were EEG responses during pain stimulation and their correlations with trait mindfulness, rather than EEG responses during mindfulness states. Therefore, our findings may only hold in the context of pain stimulation and suggest a possible mechanism by which trait mindfulness affects pain perception. Specifically, because increased δ power has been linked to the role of inhibiting all the interferences that could affect the performance of ongoing tasks,^16^ the positive correlation we found may indicate that higher levels of trait mindfulness are associated with less interference from cognitive-affective factors when maintaining attention to pain stimulation, which partly in line with findings in other studies.^13,60^ This may also suggest why individuals with high trait mindfulness are more likely to maintain awareness and attention on pain stimulation (ie, the present moment during pain stimulation), which may hinder their pain habituation.

This study has several limitations. First, while 5 participants provided fewer than 20 useable trials, prior research has used similar or fewer trials, suggesting that this reduction is unlikely to compromise the reliability of our results. Second, although *P*-values from hypothesis-driven comparisons were adjusted for multiple testing using the FDR method to mitigate the risk of false positives, the findings from exploratory analyses were not corrected and should be interpreted with caution. Third, owing to the use of a single pain intensity level (80/100) and a restricted sample (healthy individuals aged 20–30 years without mindfulness training, sex imbalance in our sample), caution should be exercised for generalizability, eg, clinical pain patients or older adults. Moreover, the sample size of our study may be underpowered for some subtle effects; however, the post-hoc power analysis showed that the power for models with significant interactions ranged from 0.615 to 0.887. In addition, trait mindfulness was assessed using the MAAS (primarily captures the attention aspect), potentially contributing to discrepancies with studies using different measures (eg, scales that integrate other aspects of mindfulness). Nevertheless, this may better differentiate the aspects of trait mindfulness that drive the observed effects.

Future studies could consider exploring varying trial numbers, alternative mindfulness assessments, and broader populations, as well as experimentally examine the speculations we use to explain the current findings, eg, coping strategy (attention or distraction from the area receiving pain stimulation) during pain stimulation in individuals with varying levels of trait mindfulness, and its impact on perceived pain intensity. Future research should measure not only pain intensity but also the pain unpleasantness to directly test whether trait mindfulness modulates the emotional dimension of pain (ie, even if pain intensity remains high, a reduction in unpleasantness may still occur, which could represent an additional relevant factor, particularly regarding potential applications in clinical settings). In addition, it should also be considered examining whether training-enhanced mindfulness can achieve effects consistent with naturally high trait mindfulness (eg, whether it causally changes the relationship between EEG and pain perception) and adding variables such as pain catastrophizing or anxiety in the models, which would help distinguish whether the effects of trait mindfulness are direct or mediated by these factors.

## 5. Conclusion

This study highlights that the predictions of θ and α power during pain stimulation for pain perception apply to individuals with lower or moderate levels of trait mindfulness, but not to individuals with higher levels of trait mindfulness. Our findings suggest that trait mindfulness is worthy of consideration as an influential factor in pain perception processes, which may thus provide some insights and a basis for clinical pain management and intervention. Moreover, our findings might suggest the impact of trait mindfulness on cognitive-affective regulation strategies and factors during pain processing, which, however, requires further validation in future studies.

## Disclosures

All authors declare that they have no competing interests.

## Supplemental digital content

Supplemental digital content associated with this article can be found online at http://links.lww.com/PR9/A342.

## Supplementary Material

SUPPLEMENTARY MATERIAL
