# Supplementary material for: The moderating role of trait mindfulness on pain perception: insights from pain-related electroencephalography oscillations
Source: Pain Rep. 2025 Oct 7;10(6):e1333. doi: 10.1097/PR9.0000000000001333 (PMC12510156; doi:10.1097/PR9.0000000000001333)
Supplement: SUPPLEMENTARY MATERIAL [file painreports-10-e1333-s001.pdf]

**Supplementary Material**

Chen Lu, Nele Berner, Lena Hagel, Nils Jannik Heukamp, Vera Moliadze*, Frauke Nees*

Institute of Medical Psychology and Medical Sociology, University Medical Center Schleswig-Holstein, Kiel University, Kiel, Germany

*Shared last authorship

**Correspondence should be addressed to**: Frauke Nees, Institute of Medical Psychology and Medical Sociology, University Medical Center Schleswig-Holstein, Kiel University, Preußerstraße 1-9 24105 Kiel, Germany.

E-mail: nees@med-psych.uni-kiel.de

Tel: +49 431 / 500-30800

Fax: +49 431 / 500-30804

## 1. The Results of the Multiple Correlation Test.

Considering that performing correlation analyses and 25 hierarchical regression models between trait mindfulness, pain intensity ratings, and five frequency bands in five regions, may increase the risk of Type I errors (false positives), we applied a false discovery rate (FDR) correction to p-values from hypothesis-driven tests of the relationship between trait mindfulness and θ/α power across five brain regions (10 tests), as well as the relationship between pain intensity ratings and α/γ power across five brain regions (10 tests). Similarly, p-values for the interaction term (trait mindfulness × θ/α/γ power) were corrected across five brain regions (15 tests).

As shown in Tables S1-S3, after p-value correction, conclusions remained consistent with those before p-value correction, except the interactions between trait mindfulness and the parietal α power ($p = 0.038$, $q = 0.081$).

Table S1. FDR Corrected p-value for the correlations between Trait Mindfulness and θ/α Power in 5 Regions.

| Test | p | q (FDR corrected p) |
|---|---|---|
| Trait mindfulness ~ Frontal θ | 0.573 | 0.769 |
| Trait mindfulness ~ Central θ | 0.626 | 0.769 |
| Trait mindfulness ~ Parietal θ | 0.300 | 0.750 |
| Trait mindfulness ~ Occipital θ | 0.201 | 0.750 |
| Trait mindfulness ~ Temporal θ | 0.218 | 0.750 |
| Trait mindfulness ~ Frontal α | 0.590 | 0.769 |
| Trait mindfulness ~ Central α | 0.614 | 0.769 |
| Trait mindfulness ~ Parietal α | 0.944 | 0.944 |
| Trait mindfulness ~ Occipital α | 0.692 | 0.769 |
| Trait mindfulness ~ Temporal α | 0.281 | 0.750 |

The p-values in the table refer to the significance of the correlation coefficients between variables before FDR (Benjamini-Hochberg). The correlation analysis mentioned here refers to the correlation analysis between variables after controlling for sex.

Table S2. FDR Corrected p-value for the correlations between Pain Intensity Ratings and α/γ Power in 5 Regions.

| Test | p | q (FDR corrected p) |
|---|---|---|
| Pain intensity ratings ~ Frontal α | 0.146 | 0.365 |
| Pain intensity ratings ~ Central α | 0.456 | 0.651 |
| Pain intensity ratings ~ Parietal α | 0.060 | 0.300 |
| Pain intensity ratings ~ Occipital α | 0.099 | 0.330 |
| Pain intensity ratings ~ Temporal α | 0.656 | 0.656 |
| Pain intensity ratings ~ Frontal γ | 0.001 | 0.010 |
| Pain intensity ratings ~ Central γ | 0.336 | 0.651 |
| Pain intensity ratings ~ Parietal γ | 0.649 | 0.656 |
| Pain intensity ratings ~ Occipital γ | 0.532 | 0.656 |
| Pain intensity ratings ~ Temporal γ | 0.444 | 0.651 |

The p-values in the table refer to the significance of the correlation coefficients between varia bles before FDR(Benjamini-Hochberg). The correlation analysis mentioned here refers to the correlation analysis between variables after controlling for sex.

Table S3. FDR Corrected p-value for the Interactions between Trait Mindfulness and θ/α/γ Po wer in 5 Regions on Influencing Pain Intensity Ratings.

| Test | p | q (FDR corrected p) |
|---|---|---|
| Trait mindfulness ~ Frontal θ | 0.147 | 0.221 |
| Trait mindfulness ~ Central θ | 0.054 | 0.090 |
| Trait mindfulness ~ Parietal θ | 0.012 | 0.035 |
| Trait mindfulness ~ Occipital θ | 0.011 | 0.035 |
| Trait mindfulness ~ Temporal θ | 0.054 | 0.090 |
| Trait mindfulness ~ Frontal α | 0.014 | 0.035 |
| Trait mindfulness ~ Central α | 0.008 | 0.035 |
| Trait mindfulness ~Parietal α | 0.038 | 0.081 |
| Trait mindfulness ~ Occipital α | 0.010 | 0.035 |
| Trait mindfulness ~ Temporal α | 0.004 | 0.035 |
| Trait mindfulness ~ Frontal γ | 0.621 | 0.621 |
| Trait mindfulness ~ Central γ | 0.302 | 0.378 |
| Trait mindfulness ~Parietal γ | 0.535 | 0.596 |
| Trait mindfulness ~ Occipital γ | 0.556 | 0.596 |
| Trait mindfulness ~ Temporal γ | 0.174 | 0.237 |

The p-values in the table refer to the significance of the regression coefficient of the interactio n term of the two variables before FDR (Benjamini-Hochberg).

## 2. Results of the Tests for the Regression Model's Assumptions.

### *2.1 Model 1.*

As shown in Figure S1 and Figure S2, the normality of residuals was evaluated visually using a histogram of standardized residuals and a probability-probability plot (P-P plot). Both plots indicated that the residuals approximately followed a normal distribution.

As shown in Figure S3, variance homogeneity was examined through a scatterplot of standardized residuals against predicted values. The residual plot showed no discernible pattern, suggesting constant variance across levels of the predictor variables.

Autocorrelation was assessed using the Durbin-Watson statistic, with values = 1.685, near 2, indicating no serious first-order autocorrelation.

As shown in Table S4, multicollinearity was evaluated by calculating both the tolerance and the variance inflation factor (VIF) for each predictor variable. All tolerance values were above 0.1, and all VIF values were well below the threshold of 10, indicating a low risk of multicollinearity.

A casewise diagnostic was also performed, identifying observations with standardized residuals exceeding three standard deviations to detect influential outliers; no such cases were found, suggesting the model is robust and not unduly affected by extreme values. Together,

these diagnostics confirm that the model satisfies the fundamental assumptions, supporting the reliability of the regression estimates.

**Figure S1.**

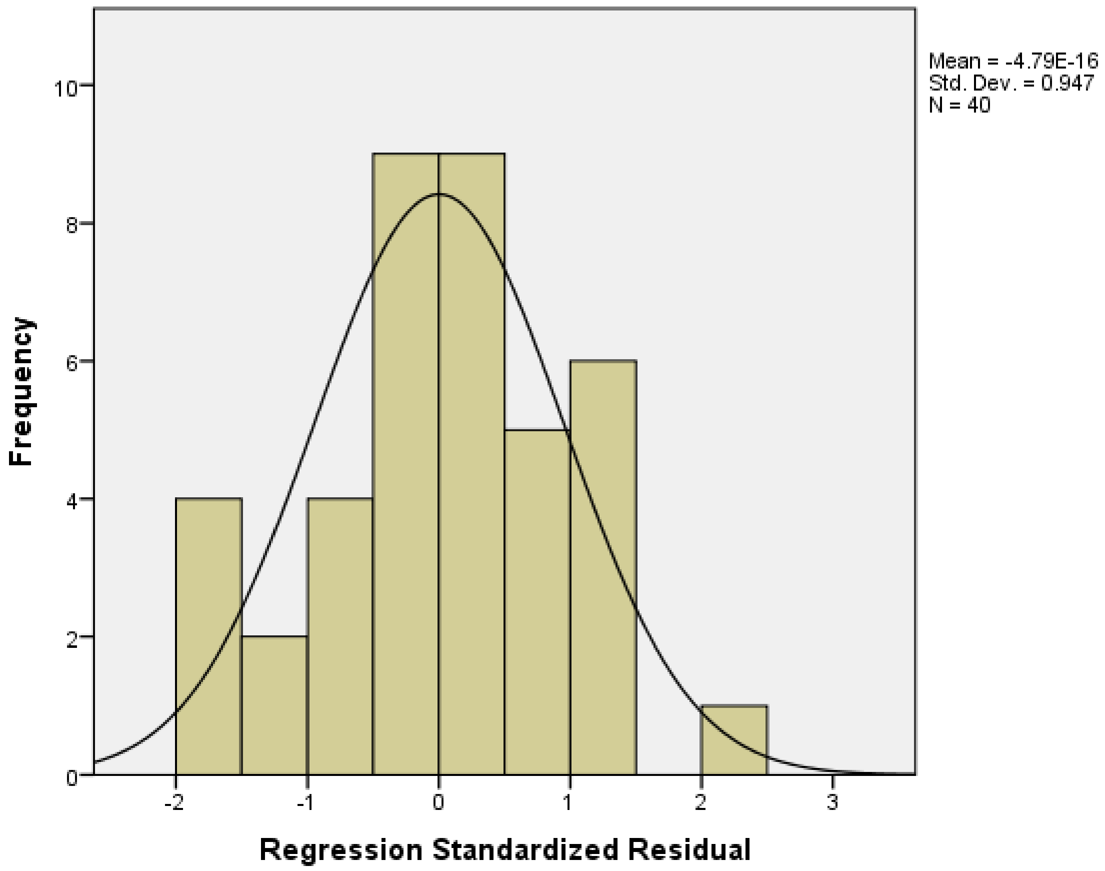


**Histogram of Standardized Residuals**

The dependent variable is the mean of pain intensity ratings.

**Figure S2.**

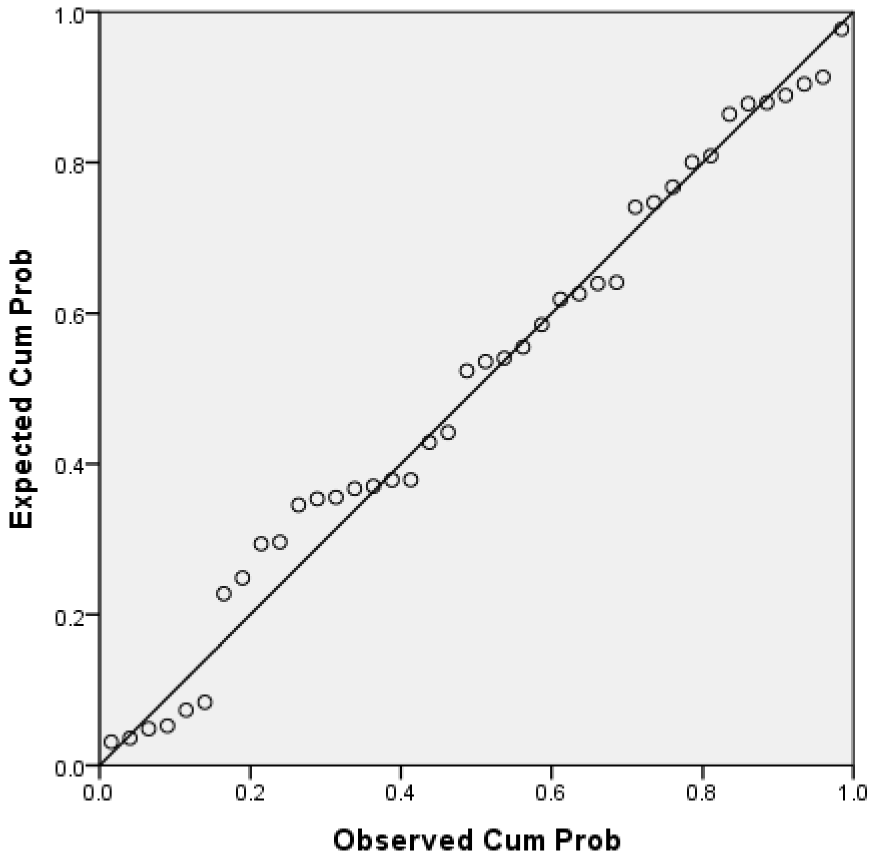


**Normal P-P Plot of Regression Standardized Residual.**

The dependent variable is the mean of pain intensity ratings.

**Figure S3.**

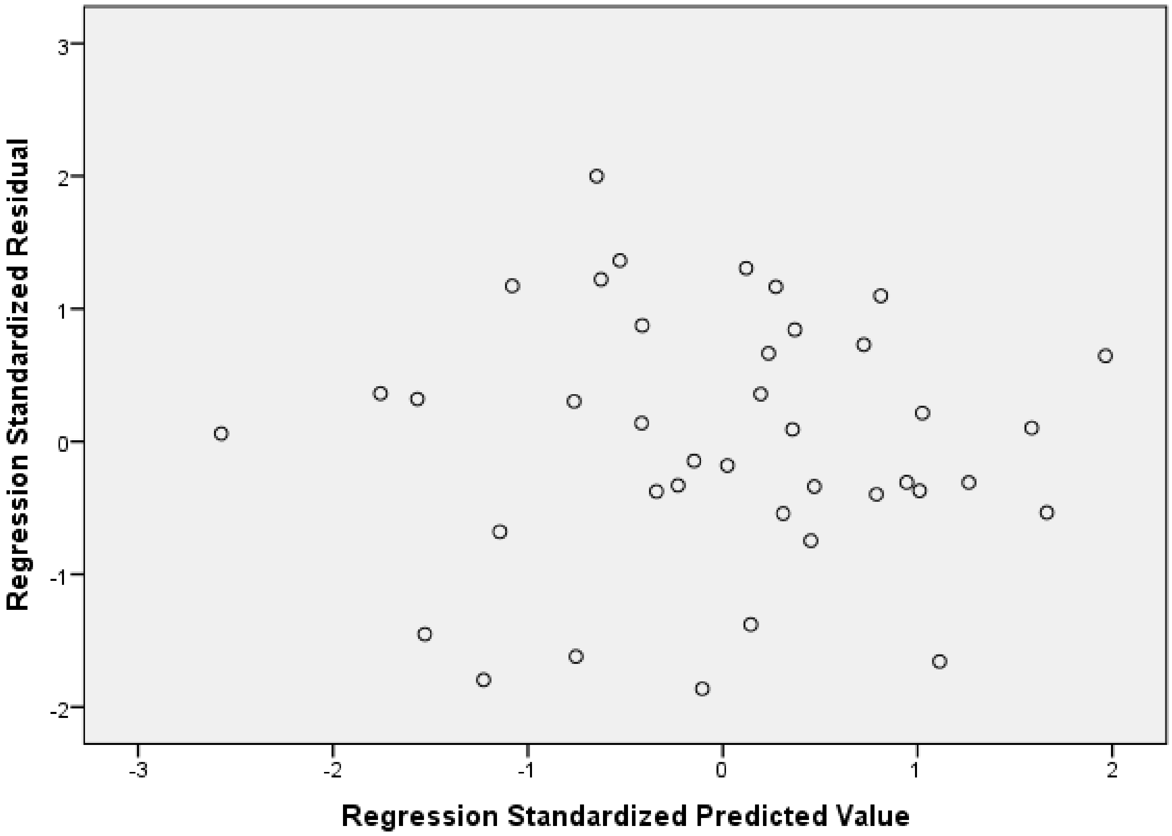


**Scatterplot.**

The dependent variable is the mean of pain intensity ratings.

Table S5. The Tolerance and the Variance Inflation Factor for Each Predictor Variable.

| Steps | Variables | Collinearity Statistics | |
|---|---|---|---|
| | | Tolerance | VIF |
| 1 | Frontal δ | 0.979 | 1.022 |
| | Trait Mindfulness | 0.979 | 1.022 |
| 2 | Frontal δ | 0.935 | 1.070 |
| | Trait Mindfulness | 0.838 | 1.193 |
| | Sex | 0.838 | 1.193 |
| 3 | Frontal δ | 0.721 | 1.387 |
| | Trait Mindfulness | 0.828 | 1.208 |
| | Sex | 0.798 | 1.254 |
| | Frontal δXTrait Mindfulness | 0.698 | 1.432 |

The dependent variable is the mean of pain intensity ratings. All continuous independent variables were centered (original values minus mean) before being entered into the models.

*2.2 Model 2.*

As shown in Figure S4 and Figure S5, the normality of residuals was evaluated visually using a histogram of standardized residuals and a probability-probability plot (P-P plot). Both plots indicated that the residuals approximately followed a normal distribution.

As shown in Figure S6, variance homogeneity was examined through a scatterplot of standardized residuals against predicted values. The residual plot showed no discernible pattern, suggesting constant variance across levels of the predictor variables.

Autocorrelation was assessed using the Durbin-Watson statistic, with values = 1.720, near 2, indicating no significant first-order autocorrelation.

As shown in Table S6, multicollinearity was evaluated by calculating both the tolerance and the variance inflation factor (VIF) for each predictor variable. All tolerance values were above 0.1, and all VIF values were well below the threshold of 10, indicating a low risk of multicollinearity.

A casewise diagnostic was also performed, identifying observations with standardized residuals exceeding three standard deviations to detect influential outliers; no such cases were found, suggesting the model is robust and not unduly affected by extreme values. Together, these diagnostics confirm that the model satisfies the fundamental assumptions, supporting the reliability of the regression estimates.

**Figure S4.**

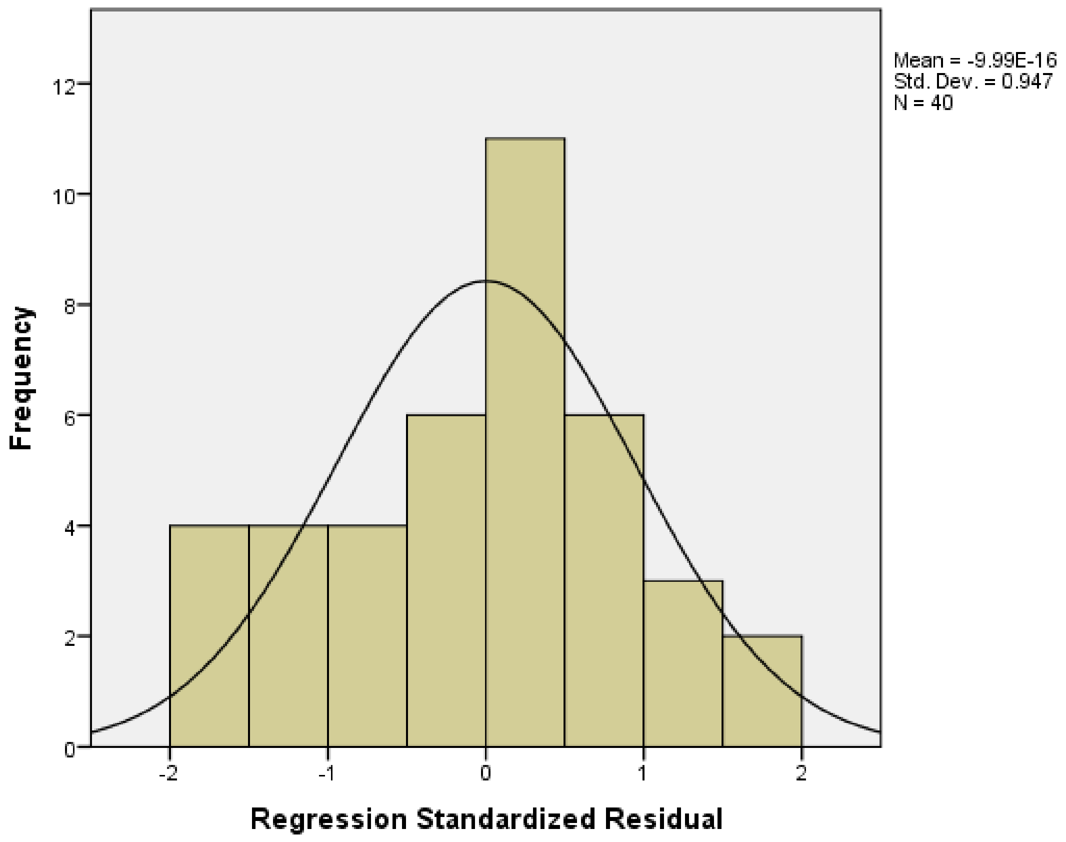


**Histogram of Standardized Residuals**

The dependent variable is the mean of pain intensity ratings.

**Figure S5.**

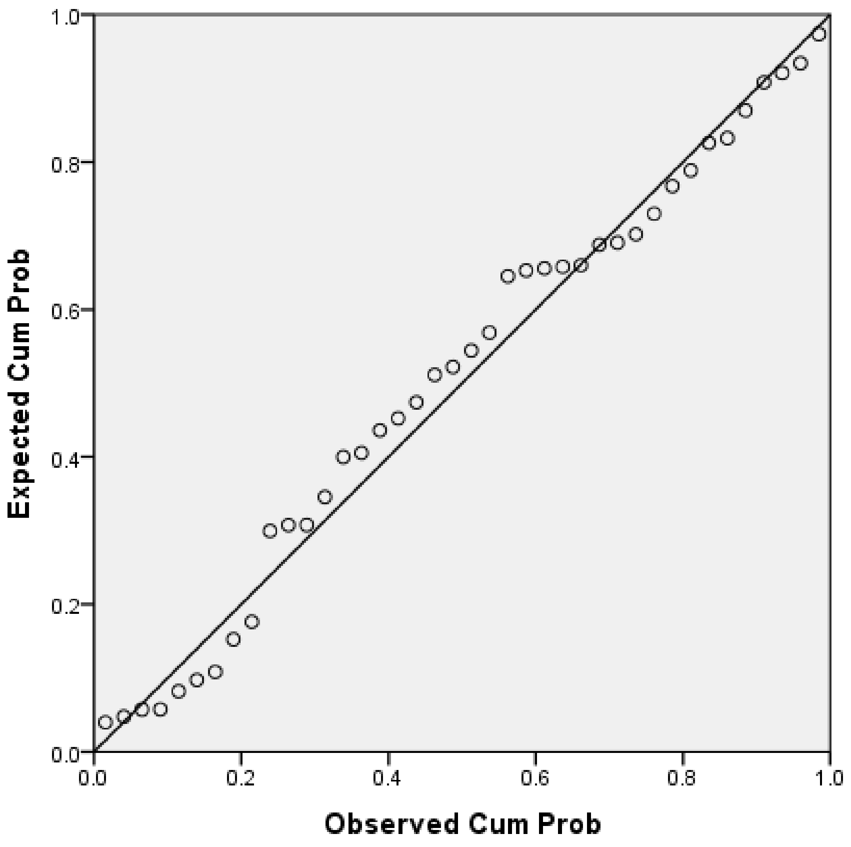


**Normal P-P Plot of Regression Standardized Residual.**

The dependent variable is the mean of pain intensity ratings.

**Figure S6.**

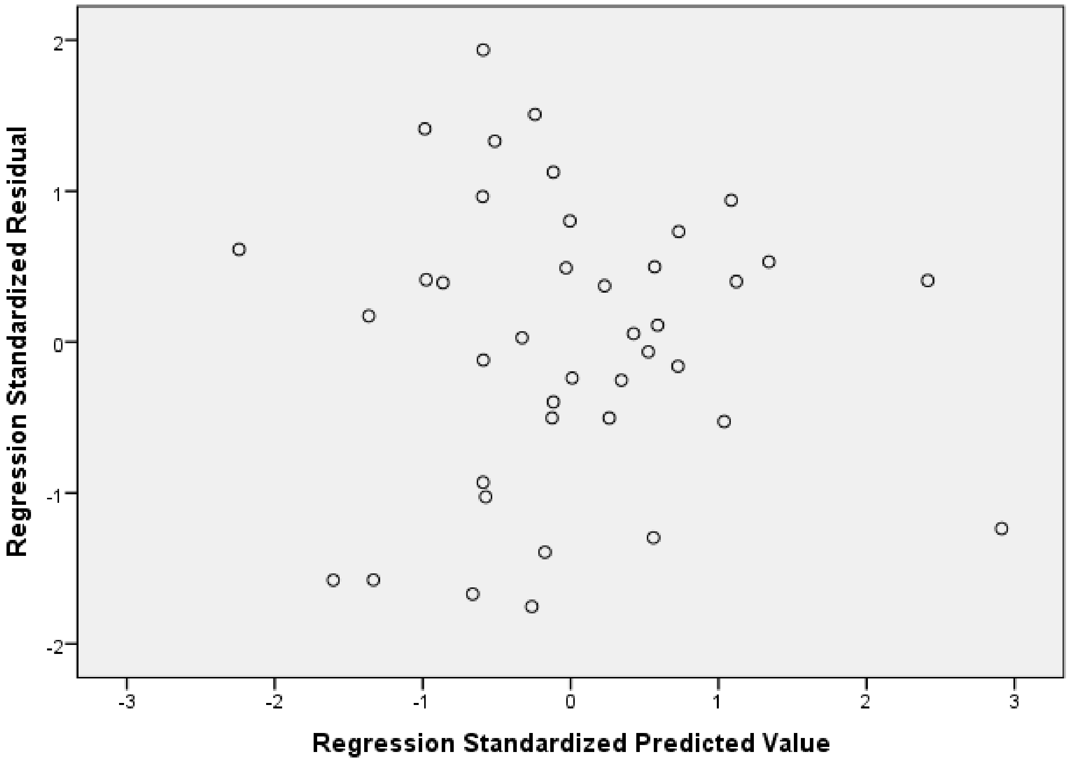


**Scatterplot.**

The dependent variable is the mean of pain intensity ratings.

Table S6. The Tolerance and the Variance Inflation Factor for Each Predictor Variable.

| Steps | Variables | Collinearity Statistics | |
|---|---|---|---|
| | | Tolerance | VIF |
| | Central δ | 0.985 | 1.015 |
| | Trait Mindfulness | 0.985 | 1.015 |
| | Central δ | 0.947 | 1.056 |
| | Trait Mindfulness | 0.848 | 1.179 |
| | Sex | 0.844 | 1.186 |
| | Central δ | 0.588 | 1.701 |
| | Trait Mindfulness | 0.738 | 1.355 |
| | Sex | 0.826 | 1.211 |
| | Central δ × Trait Mindfulness | 0.522 | 1.917 |

The dependent variable is the mean of pain intensity ratings. All continuous independent variables were centered (original values minus mean) before being entered into the models.

*2.3 Model 3.*

As shown in Figure S7 and Figure S8, the normality of residuals was evaluated visually using a histogram of standardized residuals and a probability-probability plot (P-P plot). Both plots indicated that the residuals approximately followed a normal distribution.

As shown in Figure S9, variance homogeneity was examined through a scatterplot of standardized residuals against predicted values. The residual plot showed no discernible pattern, suggesting constant variance across levels of the predictor variables.

Autocorrelation was assessed using the Durbin-Watson statistic, with values = 1.693, near 2, indicating no serious first-order autocorrelation.

As shown in Table S7, multicollinearity was evaluated by calculating both the tolerance and the variance inflation factor (VIF) for each predictor variable. All tolerance values were above 0.1, and all VIF values were well below the threshold of 10, indicating a low risk of multicollinearity.

A casewise diagnostic was also performed, identifying observations with standardized residuals exceeding three standard deviations to detect influential outliers; no such cases were found, suggesting the model is robust and not unduly affected by extreme values. Together, these diagnostics confirm that the model satisfies the fundamental assumptions, supporting the reliability of the regression estimates.

**Figure S7.**

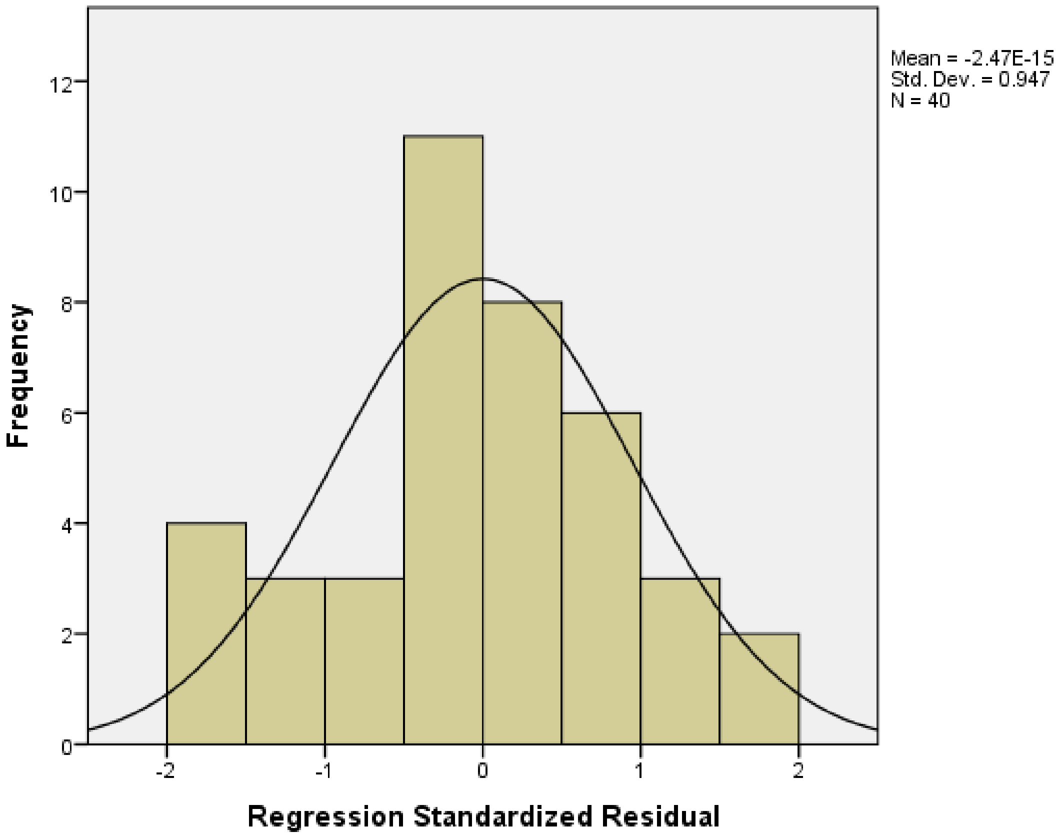


**Histogram of Standardized Residuals**

The dependent variable is the mean of pain intensity ratings.

**Figure S8.**

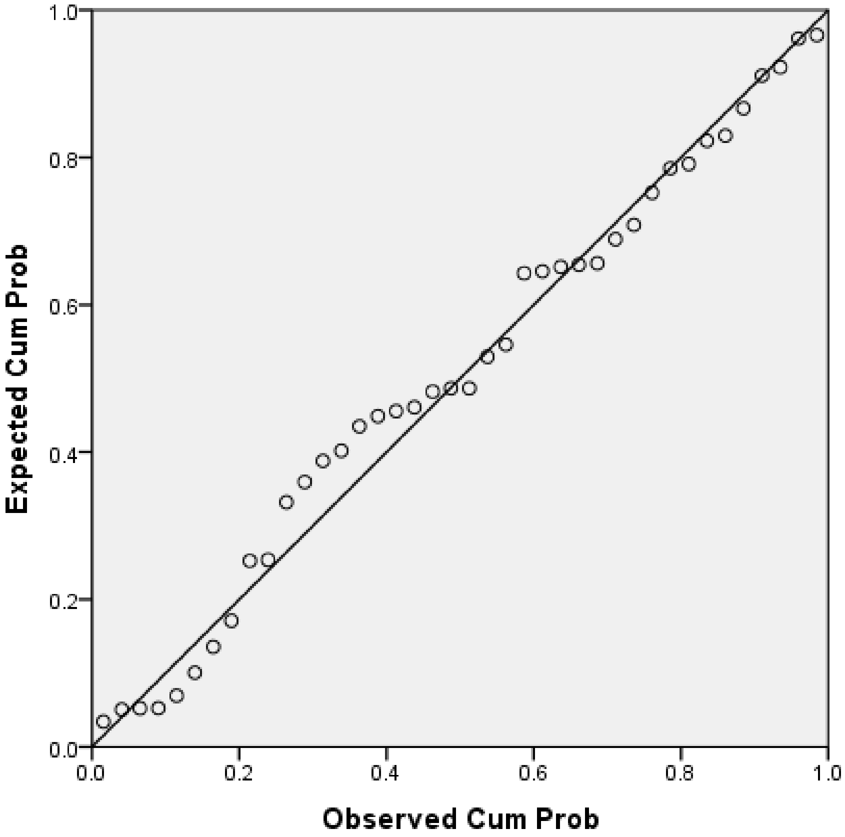


**Normal P-P Plot of Regression Standardized Residual.**

The dependent variable is the mean of pain intensity ratings.

**Figure S9.**

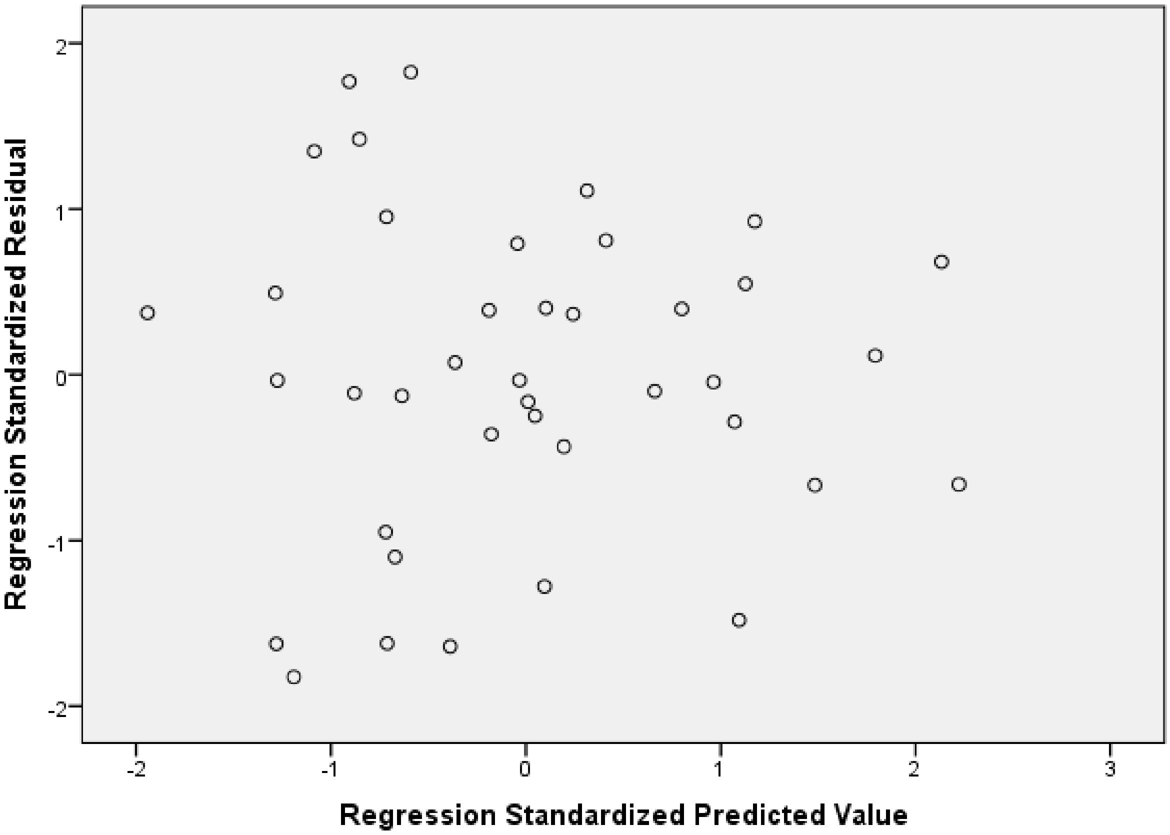


**Scatterplot.**

The dependent variable is the mean of pain intensity ratings.

Table S7. The Tolerance and the Variance Inflation Factor for Each Predictor Variable.

| Steps | Variables | Collinearity Statistics | |
|---|---|---|---|
| | | Tolerance | VIF |
| 1 | Parietal δ | 0.999 | 1.001 |
| | Trait Mindfulness | 0.999 | 1.001 |
| 2 | Parietal δ | 0.995 | 1.005 |
| | Trait Mindfulness | 0.878 | 1.139 |
| | Sex | 0.874 | 1.144 |
| 3 | Parietal δ | 0.941 | 1.063 |
| | Trait Mindfulness | 0.876 | 1.142 |
| | Sex | 0.698 | 1.434 |
| | Parietal δ × Trait Mindfulness | 0.742 | 1.348 |

The dependent variable is the mean of pain intensity ratings. All continuous independent variables were centered (original values minus mean) before being entered into the models.

*2.4 Model 4.*

As shown in Figure S10 and Figure S11, the normality of residuals was evaluated visually using a histogram of standardized residuals and a probability-probability plot (P-P plot). Both plots indicated that the residuals approximately followed a normal distribution.

As shown in Figure S12, variance homogeneity was examined through a scatterplot of standardized residuals against predicted values. The residual plot showed no discernible pattern, suggesting constant variance across levels of the predictor variables.

Autocorrelation was assessed using the Durbin-Watson statistic, with values = 1.672, near 2, indicating no serious first-order autocorrelation.

As shown in Table S8, multicollinearity was evaluated by calculating both the tolerance and the variance inflation factor (VIF) for each predictor variable. All tolerance values were above 0.1, and all VIF values were well below the threshold of 10, indicating a low risk of multicollinearity.

A casewise diagnostic was also performed, identifying observations with standardized residuals exceeding three standard deviations to detect influential outliers; no such cases were found, suggesting the model is robust and not unduly affected by extreme values. Together, these diagnostics confirm that the model satisfies the fundamental assumptions, supporting the reliability of the regression estimates.

**Figure S10.**

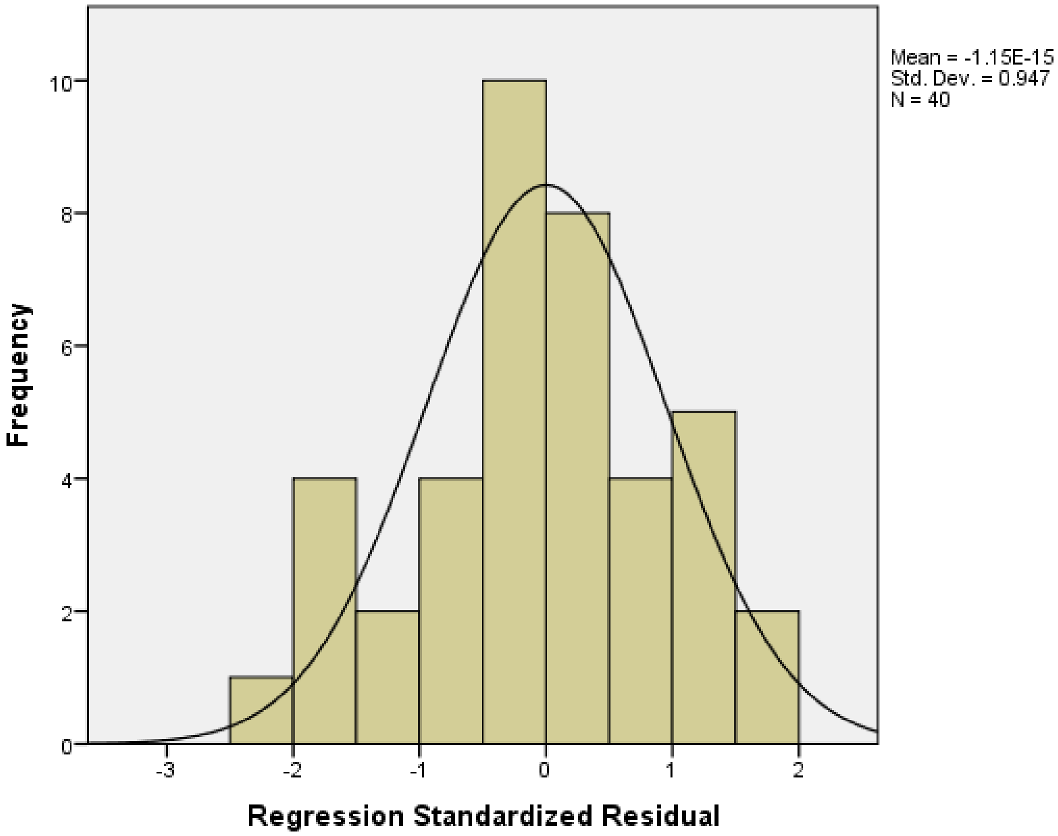


**Histogram of Standardized Residuals**

The dependent variable is the mean of pain intensity ratings.

**Figure S11.**

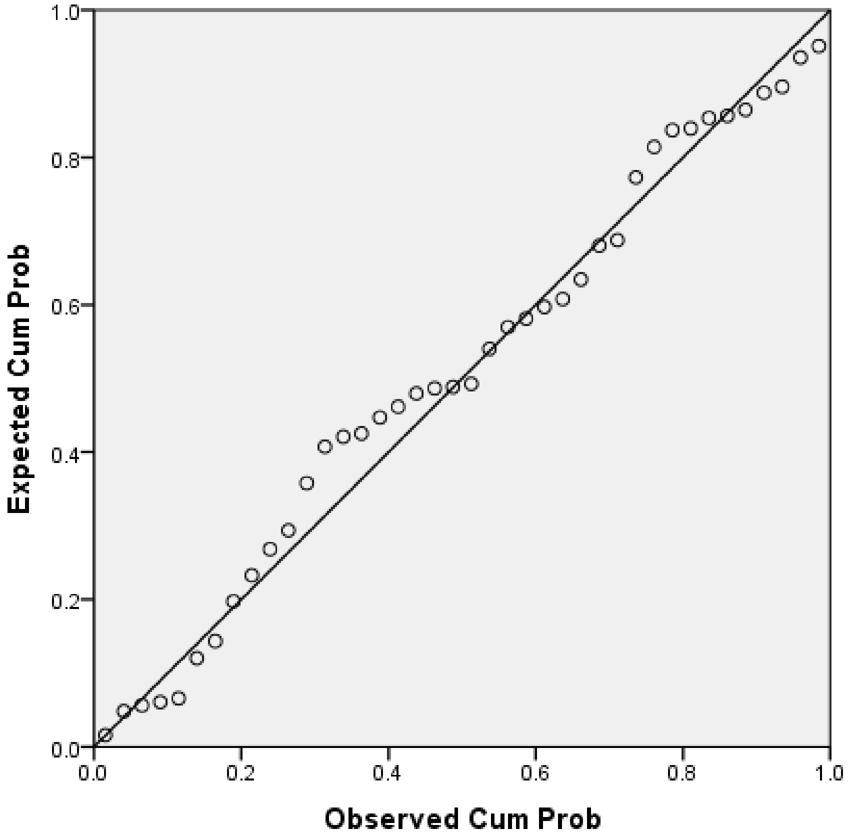


**Normal P-P Plot of Regression Standardized Residual.**

The dependent variable is the mean of pain intensity ratings.

**Figure S12.**

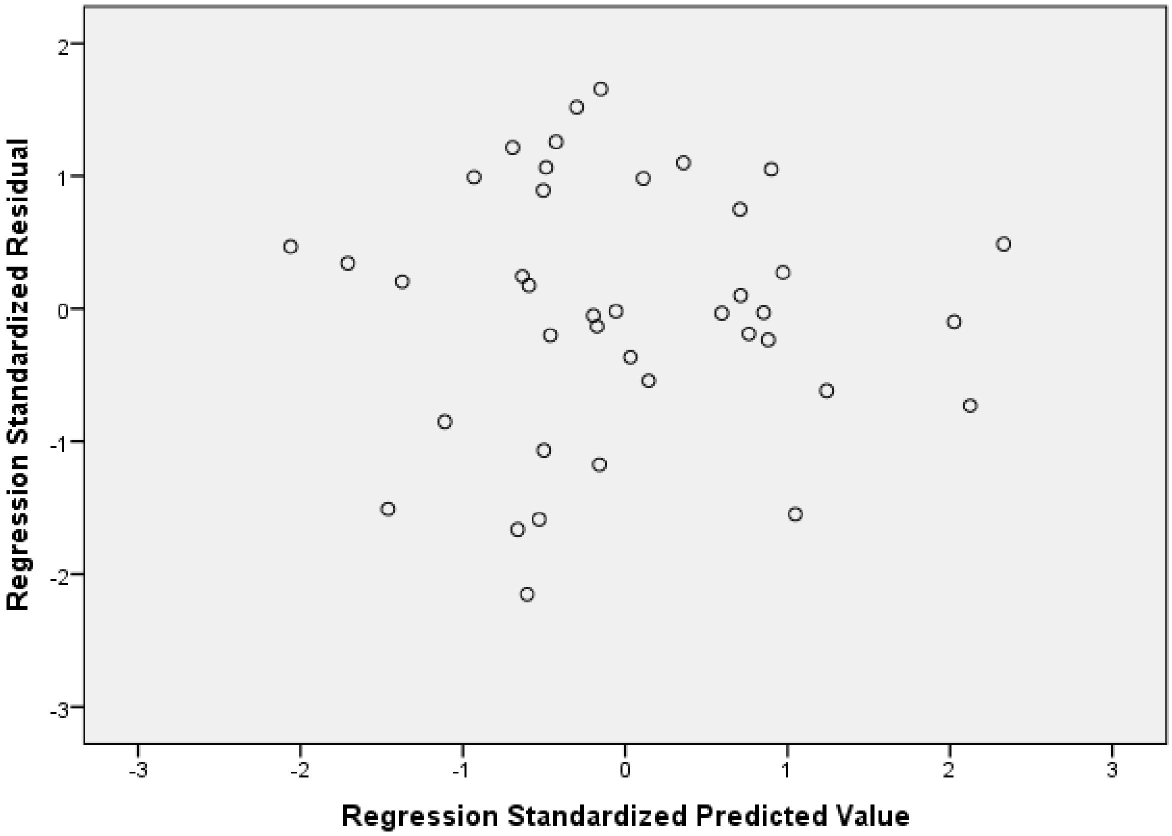


**Scatterplot.**

The dependent variable is the mean of pain intensity ratings.

Table S8. The Tolerance and the Variance Inflation Factor for Each Predictor Variable.

| Steps | Variables | Collinearity Statistics | |
|---|---|---|---|
| | | Tolerance | VIF |
| 1 | Occipital δ | 0.935 | 1.070 |
| | Trait Mindfulness | 0.935 | 1.070 |
| 2 | Occipital δ | 0.758 | 1.319 |
| | Trait Mindfulness | 0.733 | 1.363 |
| | Sex | 0.712 | 1.405 |
| 3 | Occipital δ | 0.551 | 1.815 |
| | Trait Mindfulness | 0.686 | 1.457 |
| | Sex | 0.683 | 1.464 |
| | Occipital δXTrait Mindfulness | 0.552 | 1.811 |

The dependent variable is the mean of pain intensity ratings. All continuous independent variables were centered (original values minus mean) before being entered into the models.

*2.5 Model 5.*

As shown in Figure S13 and Figure S14, the normality of residuals was evaluated visually using a histogram of standardized residuals and a probability-probability plot (P-P plot). Both plots indicated that the residuals approximately followed a normal distribution.

As shown in Figure S15, variance homogeneity was examined through a scatterplot of standardized residuals against predicted values. The residual plot showed no discernible pattern, suggesting constant variance across levels of the predictor variables.

Autocorrelation was assessed using the Durbin-Watson statistic, with values = 1.694, near 2, indicating no serious first-order autocorrelation.

As shown in Table S9, multicollinearity was evaluated by calculating both the tolerance and the variance inflation factor (VIF) for each predictor variable. All tolerance values were above 0.1, and all VIF values were well below the threshold of 10, indicating a low risk of multicollinearity.

A casewise diagnostic was also performed, identifying observations with standardized residuals exceeding three standard deviations to detect influential outliers; no such cases were found, suggesting the model is robust and not unduly affected by extreme values. Together, these diagnostics confirm that the model satisfies the fundamental assumptions, supporting the reliability of the regression estimates.

**Figure S13.**

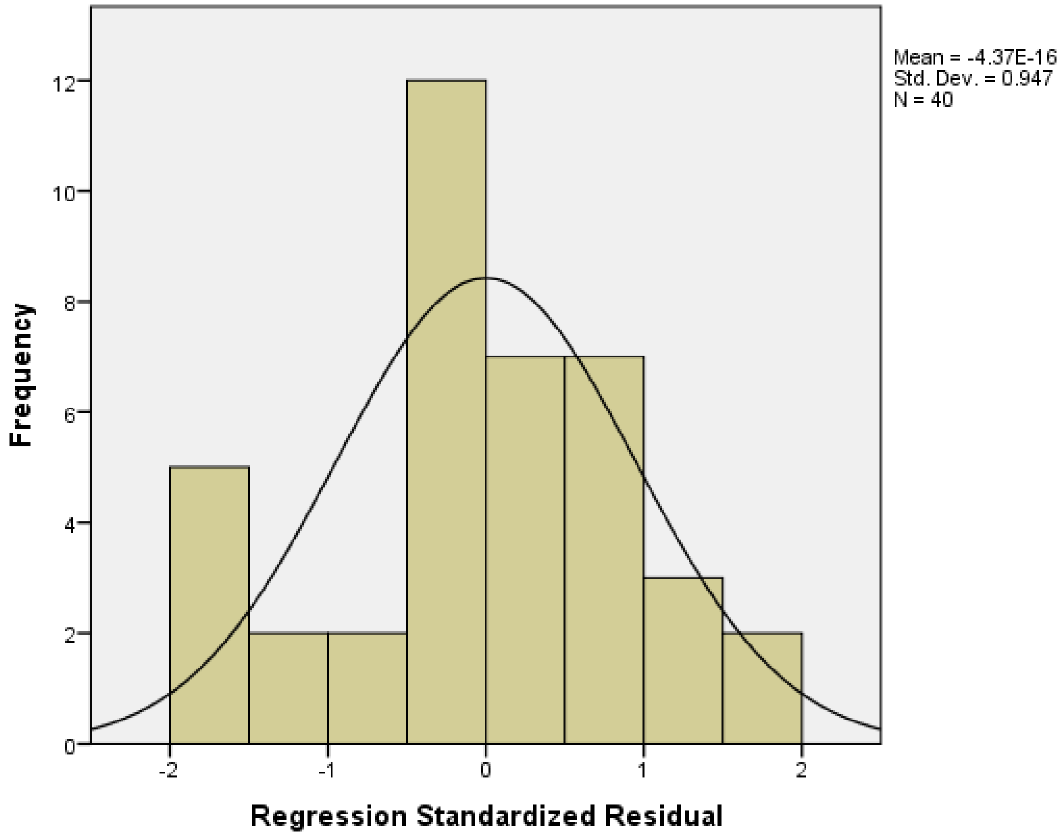


**Histogram of Standardized Residuals**

The dependent variable is the mean of pain intensity ratings.

**Figure S14.**

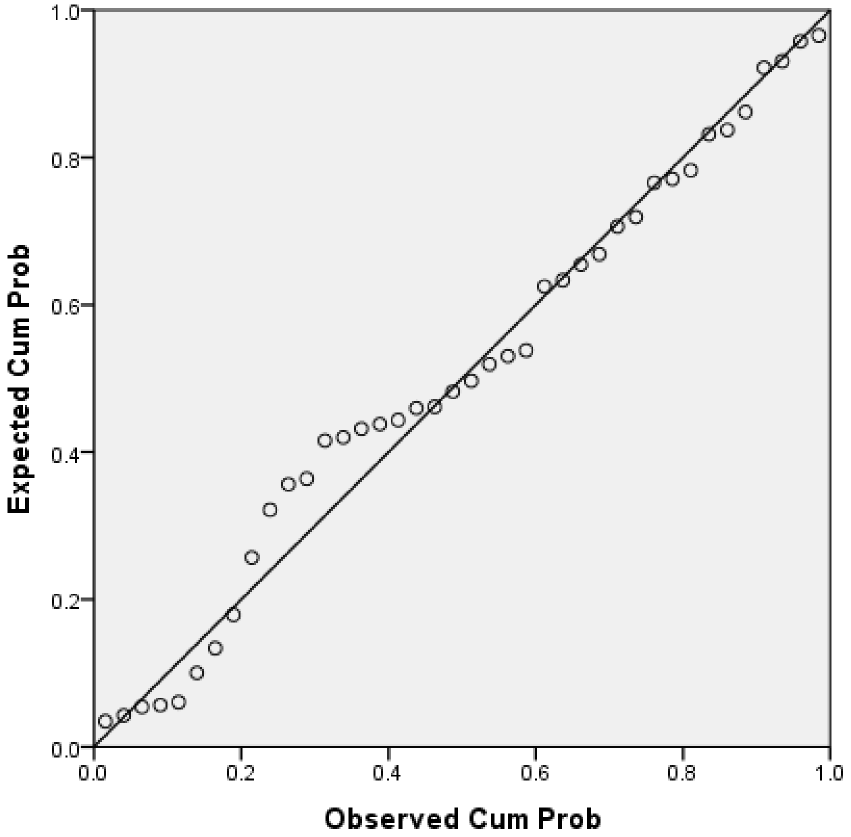


**Normal P-P Plot of Regression Standardized Residual.**

The dependent variable is the mean of pain intensity ratings.

**Figure S15.**

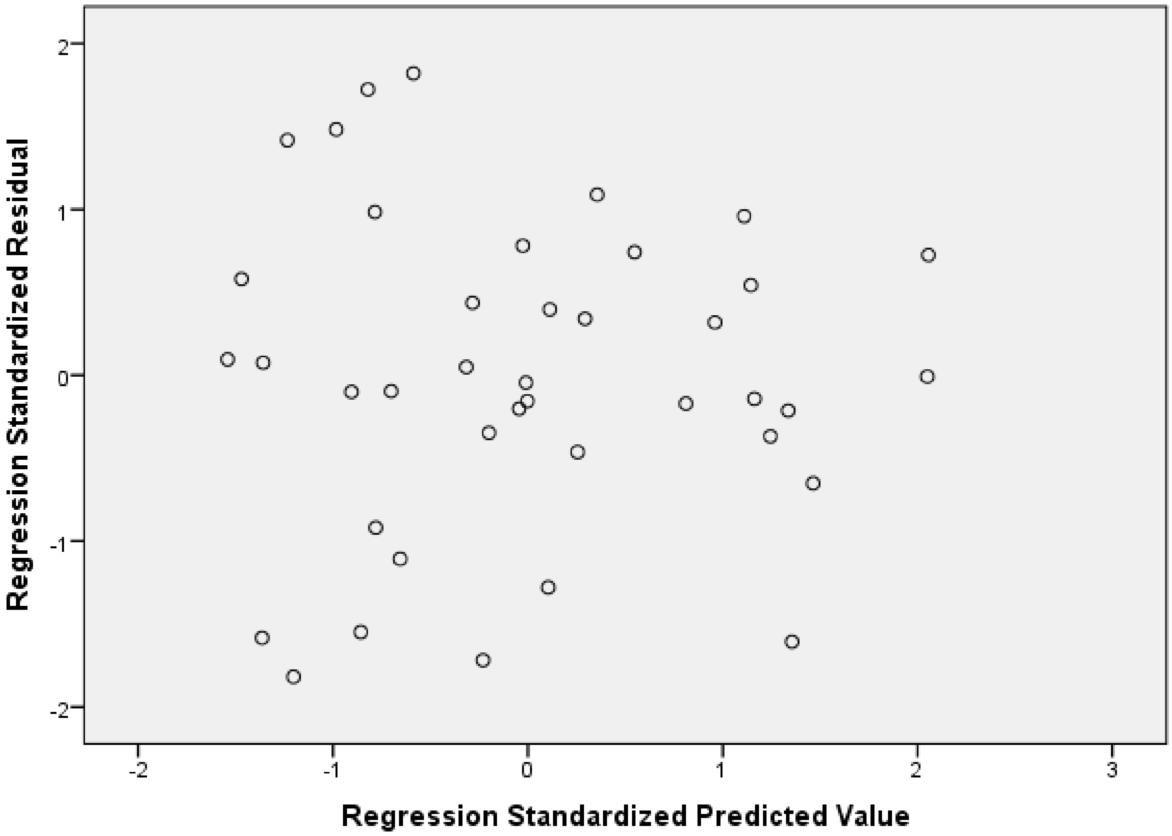


**Scatterplot.**

The dependent variable is the mean of pain intensity ratings.

Table S9. The Tolerance and the Variance Inflation Factor for Each Predictor Variable.

| Steps | Variables | Collinearity Statistics | |
|---|---|---|---|
| | | Tolerance | VIF |
| 1 | Temporal δ | 0.956 | 1.046 |
| | Trait Mindfulness | 0.956 | 1.046 |
| 2 | Temporal δ | 0.892 | 1.121 |
| | Trait Mindfulness | 0.804 | 1.243 |
| | Sex | 0.819 | 1.221 |
| 3 | Temporal δ | 0.220 | 4.550 |
| | Trait Mindfulness | 0.804 | 1.244 |
| | Sex | 0.819 | 1.221 |
| | Temporal δXTrait Mindfulness | 0.226 | 4.423 |

The dependent variable is the mean of pain intensity ratings. All continuous independent variables were centered (original values minus mean) before being entered into the models.

*2.6 Model 6.*

As shown in Figure S16 and Figure S17, the normality of residuals was evaluated visually using a histogram of standardized residuals and a probability-probability plot (P-P plot). Both plots indicated that the residuals approximately followed a normal distribution.

As shown in Figure S18, variance homogeneity was examined through a scatterplot of standardized residuals against predicted values. The residual plot showed no discernible pattern, suggesting constant variance across levels of the predictor variables.

Autocorrelation was assessed using the Durbin-Watson statistic, with values = 1.683, near 2, indicating no serious first-order autocorrelation.

As shown in Table S10, multicollinearity was evaluated by calculating both the tolerance and the variance inflation factor (VIF) for each predictor variable. All tolerance values were above 0.1, and all VIF values were well below the threshold of 10, indicating a low risk of multicollinearity.

A casewise diagnostic was also performed, identifying observations with standardized residuals exceeding three standard deviations to detect influential outliers; no such cases were found, suggesting the model is robust and not unduly affected by extreme values. Together, these diagnostics confirm that the model satisfies the fundamental assumptions, supporting the reliability of the regression estimates.

**Figure S16.**

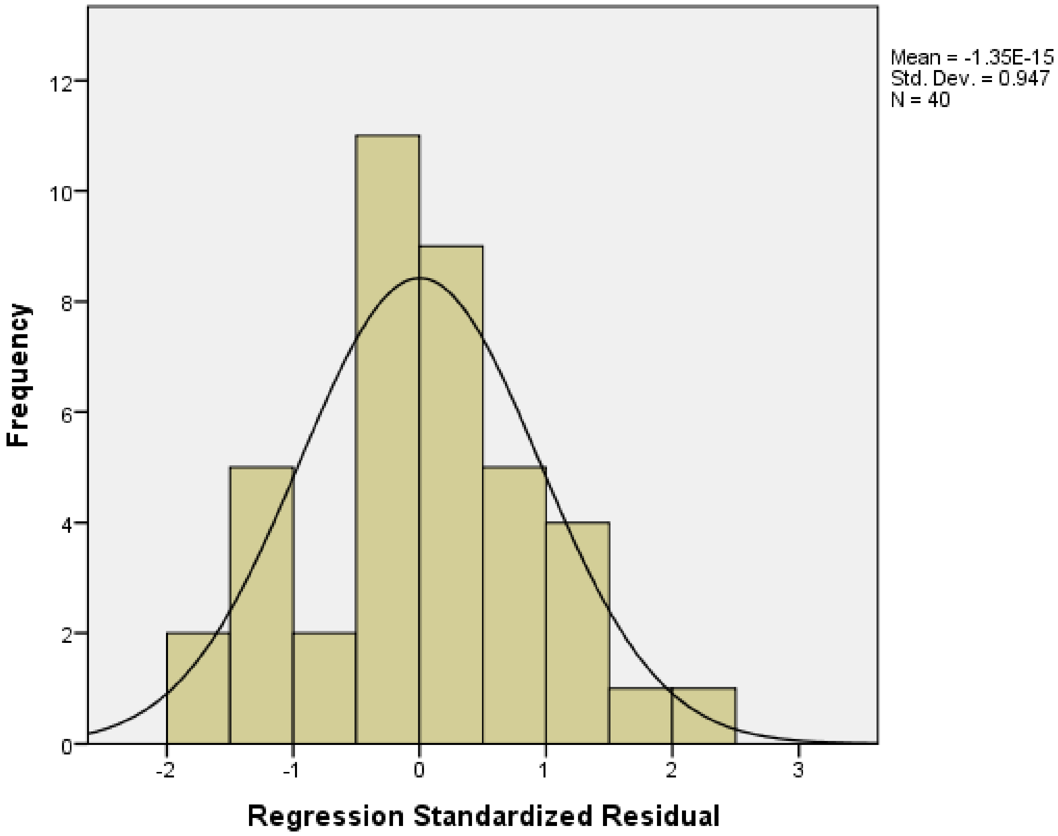


**Histogram of Standardized Residuals**

The dependent variable is the mean of pain intensity ratings.

**Figure S17.**

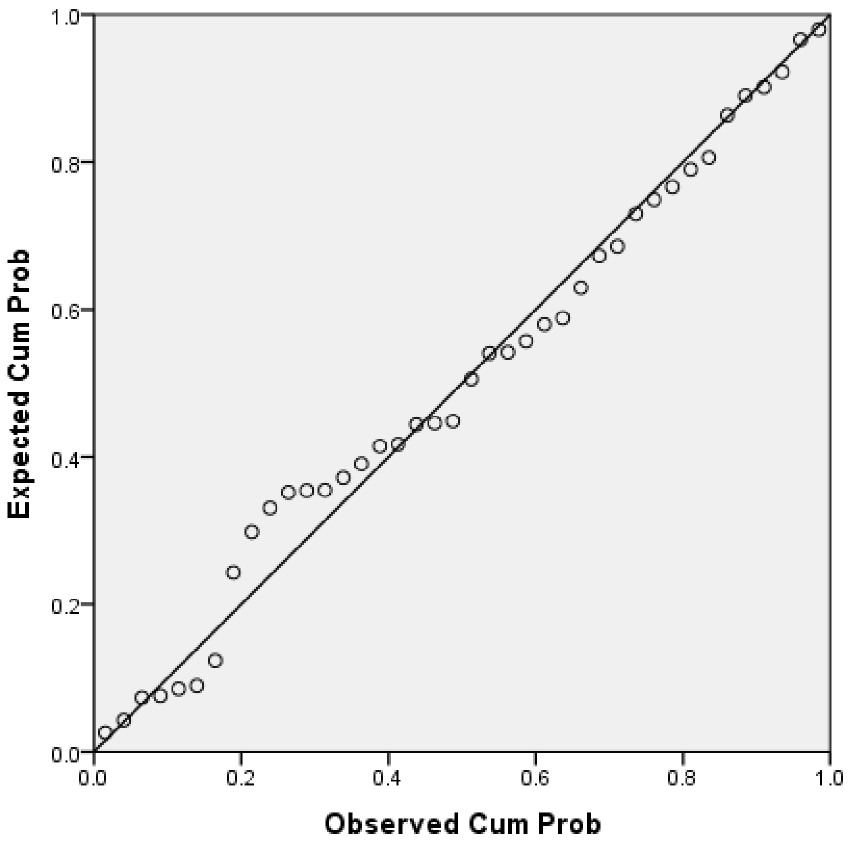


**Normal P-P Plot of Regression Standardized Residual.**

The dependent variable is the mean of pain intensity ratings.

**Figure S18.**

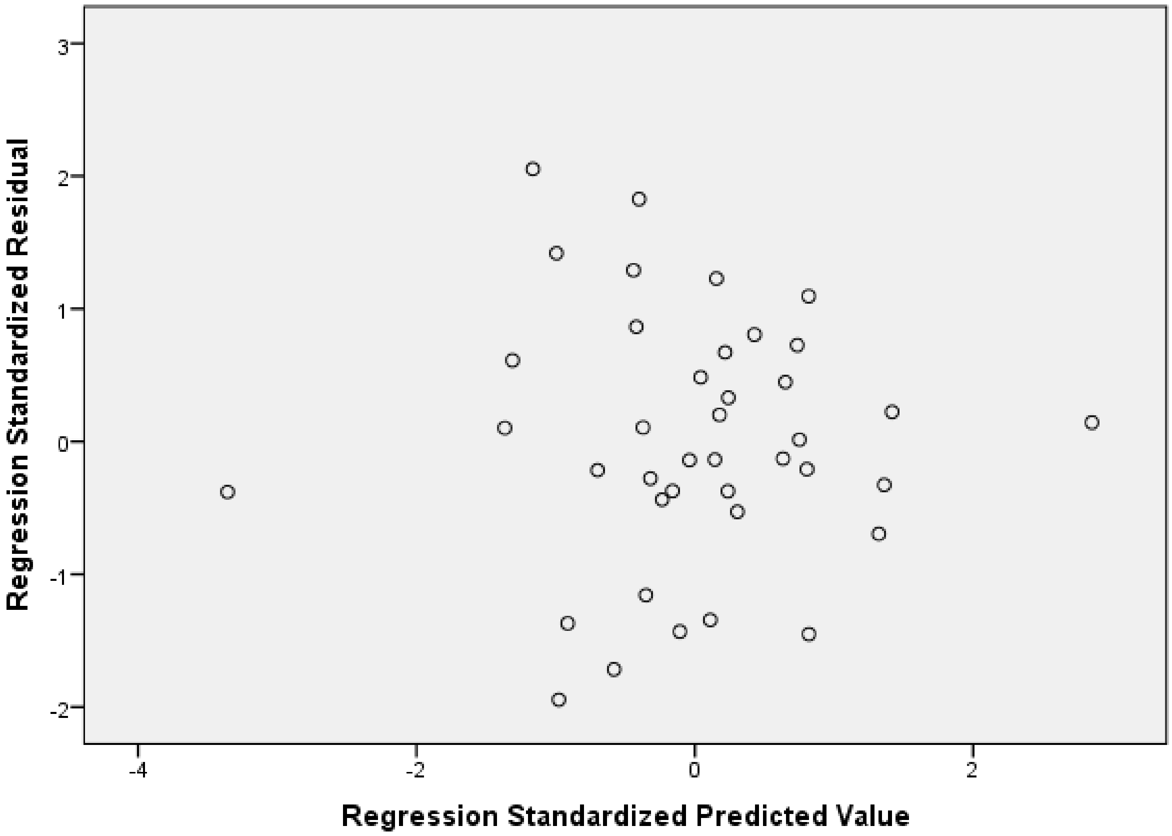


**Scatterplot.**

The dependent variable is the mean of pain intensity ratings.

Table S10. The Tolerance and the Variance Inflation Factor for Each Predictor Variable.

| Steps | Variables | Collinearity Statistics | |
|---|---|---|---|
| | | Tolerance | VIF |
| 1 | Frontal θ | 0.992 | 1.009 |
| | Trait Mindfulness | 0.992 | 1.009 |
| 2 | Frontal θ | 0.991 | 1.009 |
| | Trait Mindfulness | 0.870 | 1.149 |
| | Sex | 0.877 | 1.140 |
| 3 | Frontal θ | 0.919 | 1.088 |
| | Trait Mindfulness | 0.851 | 1.175 |
| | Sex | 0.740 | 1.352 |
| | Frontal θXTrait Mindfulness | 0.787 | 1.271 |

The dependent variable is the mean of pain intensity ratings. All continuous independent variables were centered (original values minus mean) before being entered into the models.

*2.7 Model 7.*

As shown in Figure S19 and Figure S20, the normality of residuals was evaluated visually using a histogram of standardized residuals and a probability-probability plot (P-P plot). Both plots indicated that the residuals approximately followed a normal distribution.

As shown in Figure S21, variance homogeneity was examined through a scatterplot of standardized residuals against predicted values. The residual plot showed no discernible pattern, suggesting constant variance across levels of the predictor variables.

Autocorrelation was assessed using the Durbin-Watson statistic, with values = 1.697, near 2, indicating no serious first-order autocorrelation.

As shown in Table S11, multicollinearity was evaluated by calculating both the tolerance and the variance inflation factor (VIF) for each predictor variable. All tolerance values were above 0.1, and all VIF values were well below the threshold of 10, indicating a low risk of multicollinearity.

A casewise diagnostic was also performed, identifying observations with standardized residuals exceeding three standard deviations to detect influential outliers; no such cases were found, suggesting the model is robust and not unduly affected by extreme values. Together, these diagnostics confirm that the model satisfies the fundamental assumptions, supporting the reliability of the regression estimates.

**Figure S19.**

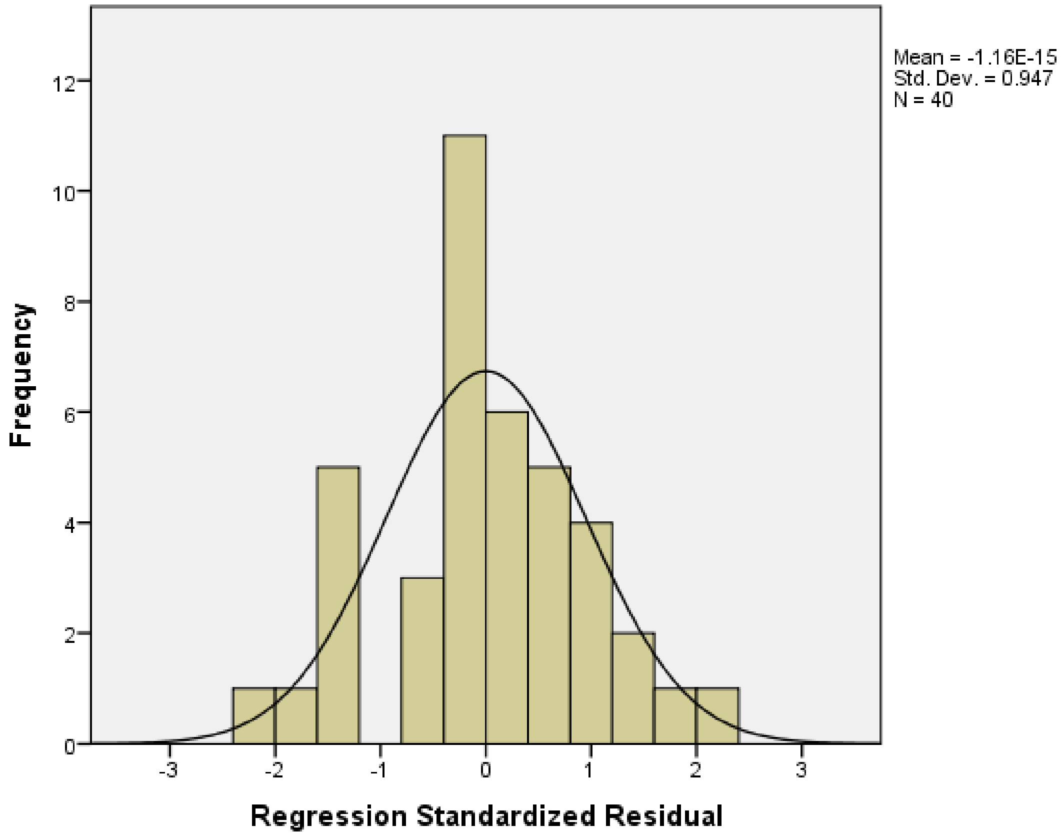


**Histogram of Standardized Residuals**

The dependent variable is the mean of pain intensity ratings.

**Figure S20.**

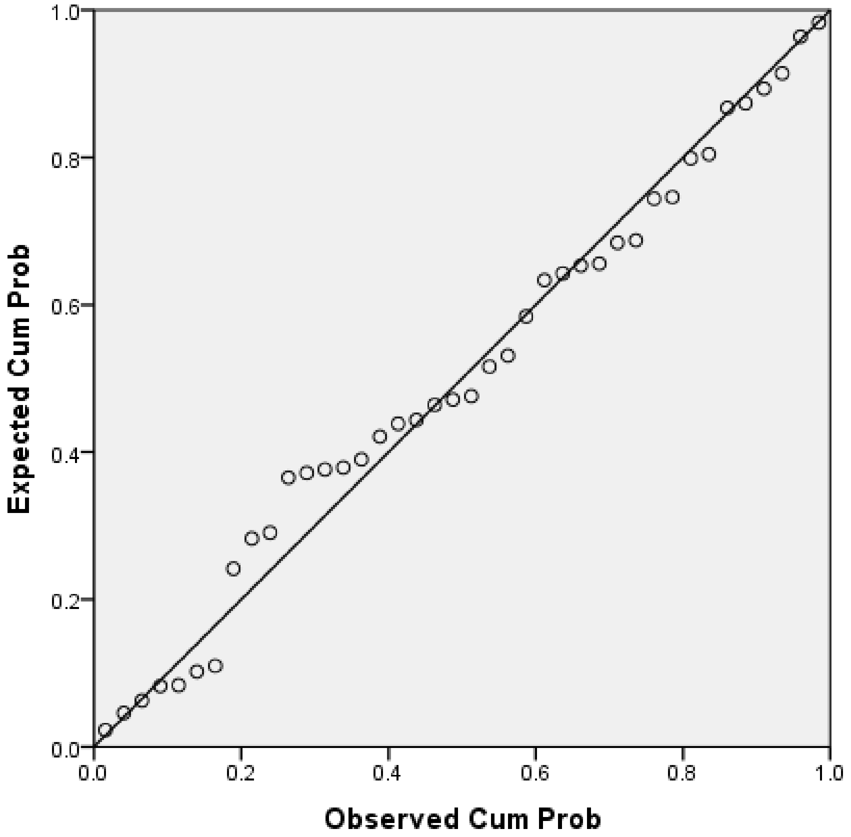


**Normal P-P Plot of Regression Standardized Residual.**

The dependent variable is the mean of pain intensity ratings.

**Figure S21.**

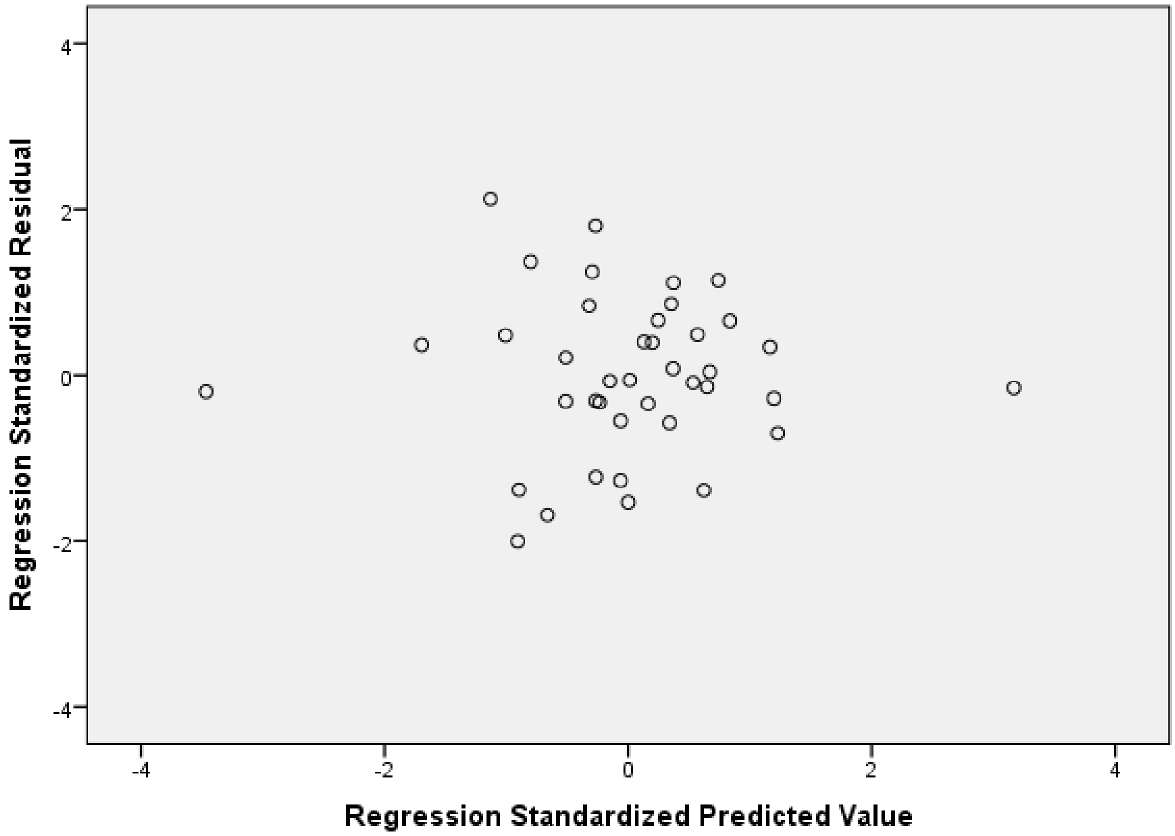


**Scatterplot.**

The dependent variable is the mean of pain intensity ratings.

Table S11. The Tolerance and the Variance Inflation Factor for Each Predictor Variable.

| Steps | Variables | Collinearity Statistics | |
|---|---|---|---|
| | | Tolerance | VIF |
| 1 | Central θ | 0.994 | 1.006 |
| | Trait Mindfulness | 0.994 | 1.006 |
| 2 | Central θ | 0.993 | 1.007 |
| | Trait Mindfulness | 0.872 | 1.147 |
| | Sex | 0.877 | 1.140 |
| 3 | Central θ | 0.920 | 1.087 |
| | Trait Mindfulness | 0.845 | 1.183 |
| | Sex | 0.753 | 1.328 |
| | Central θXTrait Mindfulness | 0.798 | 1.254 |

The dependent variable is the mean of pain intensity ratings. All continuous independent variables were centered (original values minus mean) before being entered into the models.

*2.8 Model 8.*

As shown in Figure S22 and Figure S23, the normality of residuals was evaluated visually using a histogram of standardized residuals and a probability-probability plot (P-P plot). Both plots indicated that the residuals approximately followed a normal distribution.

As shown in Figure S24, variance homogeneity was examined through a scatterplot of standardized residuals against predicted values. The residual plot showed no discernible pattern, suggesting constant variance across levels of the predictor variables.

Autocorrelation was assessed using the Durbin-Watson statistic, with values = 1.616, near 2, indicating no serious first-order autocorrelation.

As shown in Table S12, multicollinearity was evaluated by calculating both the tolerance and the variance inflation factor (VIF) for each predictor variable. All tolerance values were above 0.1, and all VIF values were well below the threshold of 10, indicating a low risk of multicollinearity.

A casewise diagnostic was also performed, identifying observations with standardized residuals exceeding three standard deviations to detect influential outliers; no such cases were found, suggesting the model is robust and not unduly affected by extreme values. Together, these diagnostics confirm that the model satisfies the fundamental assumptions, supporting the reliability of the regression estimates.

**Figure S22.**

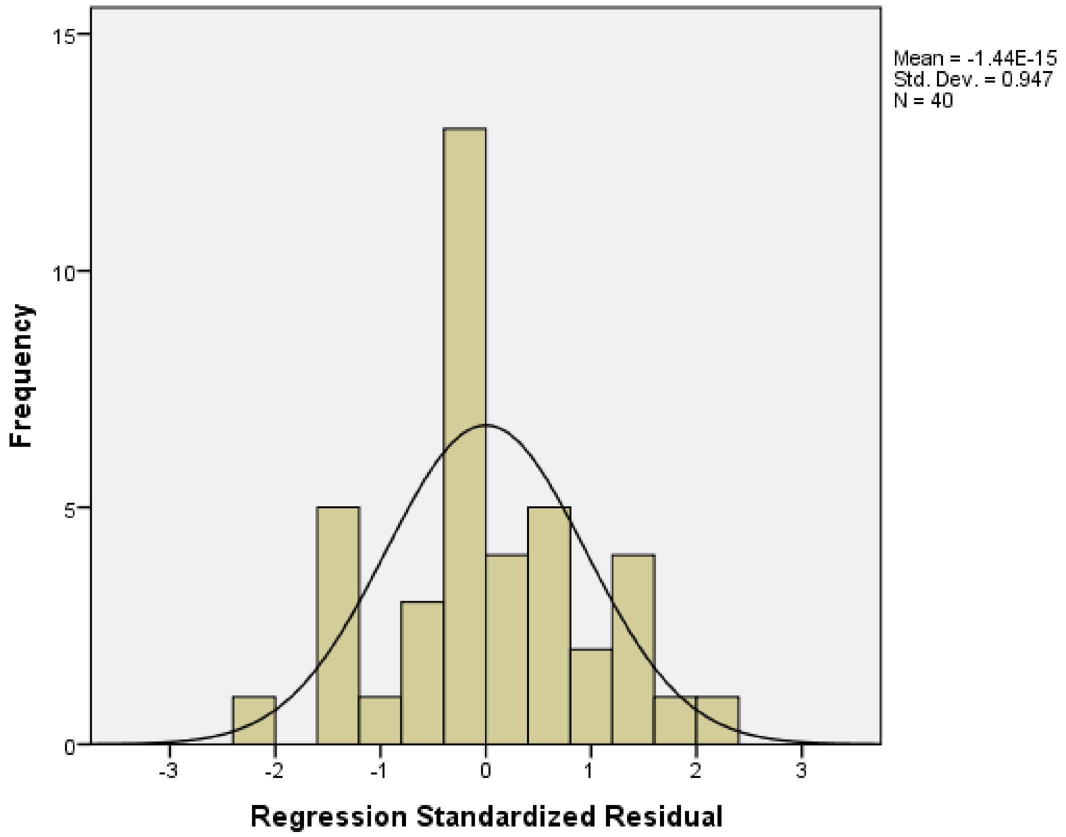


**Histogram of Standardized Residuals**

The dependent variable is the mean of pain intensity ratings.

**Figure S23.**

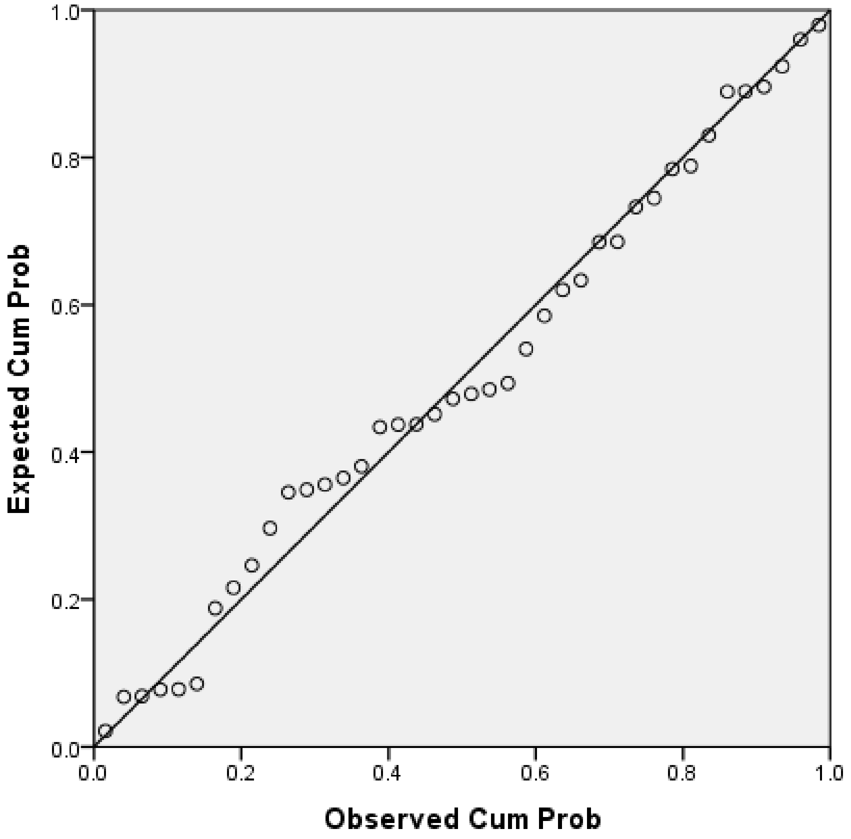


**Normal P-P Plot of Regression Standardized Residual.**

The dependent variable is the mean of pain intensity ratings.

**Figure S24.**

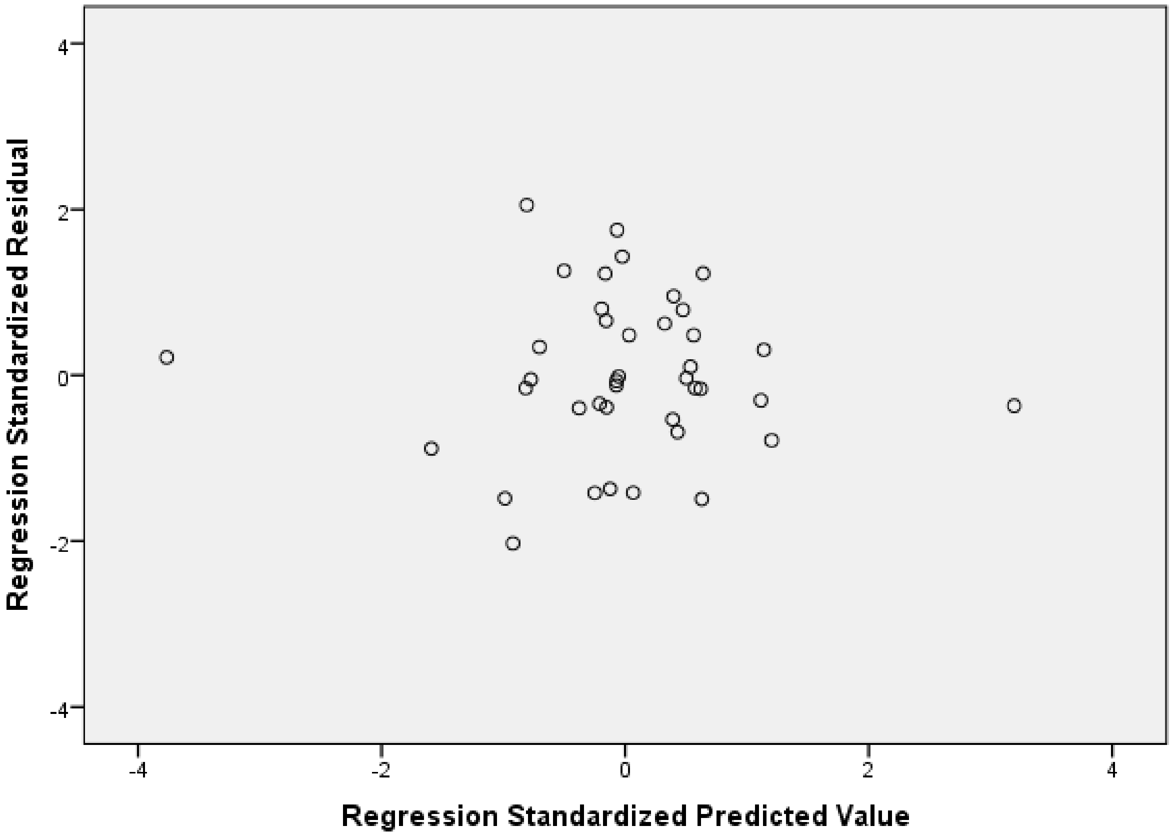


**Scatterplot.**

The dependent variable is the mean of pain intensity ratings.

Table S12. The Tolerance and the Variance Inflation Factor for Each Predictor Variable.

| Steps | Variables | Collinearity Statistics | |
|---|---|---|---|
| | | Tolerance | VIF |
| 1 | Parietal θ | 0.979 | 1.021 |
| | Trait Mindfulness | 0.979 | 1.021 |
| 2 | Parietal θ | 0.969 | 1.032 |
| | Trait Mindfulness | 0.852 | 1.173 |
| | Sex | 0.868 | 1.152 |
| 3 | Parietal θ | 0.693 | 1.443 |
| | Trait Mindfulness | 0.852 | 1.174 |
| | Sex | 0.756 | 1.322 |
| | Parietal θXTrait Mindfulness | 0.622 | 1.608 |

The dependent variable is the mean of pain intensity ratings. All continuous independent variables were centered (original values minus mean) before being entered into the models.

*2.9 Model 9.*

As shown in Figure S25 and Figure S26, the normality of residuals was evaluated visually using a histogram of standardized residuals and a probability-probability plot (P-P plot). Both plots indicated that the residuals approximately followed a normal distribution.

As shown in Figure S27, variance homogeneity was examined through a scatterplot of standardized residuals against predicted values. The residual plot showed no discernible pattern, suggesting constant variance across levels of the predictor variables.

Autocorrelation was assessed using the Durbin-Watson statistic, with values = 1.660, near 2, indicating no serious first-order autocorrelation.

As shown in Table S13, multicollinearity was evaluated by calculating both the tolerance and the variance inflation factor (VIF) for each predictor variable. All tolerance values were above 0.1, and all VIF values were well below the threshold of 10, indicating a low risk of multicollinearity.

A casewise diagnostic was also performed, identifying observations with standardized residuals exceeding three standard deviations to detect influential outliers; no such cases were found, suggesting the model is robust and not unduly affected by extreme values. Together, these diagnostics confirm that the model satisfies the fundamental assumptions, supporting the reliability of the regression estimates.

**Figure S25.**

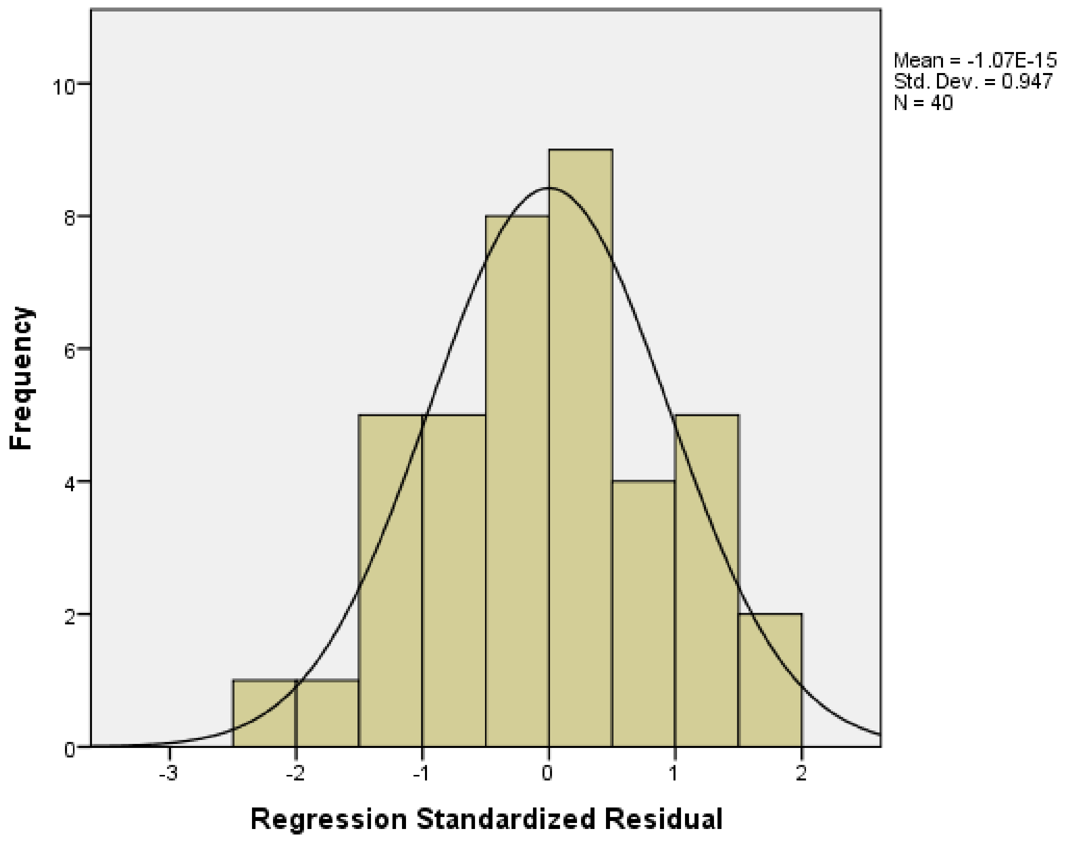


**Histogram of Standardized Residuals**

The dependent variable is the mean of pain intensity ratings.

**Figure S26.**

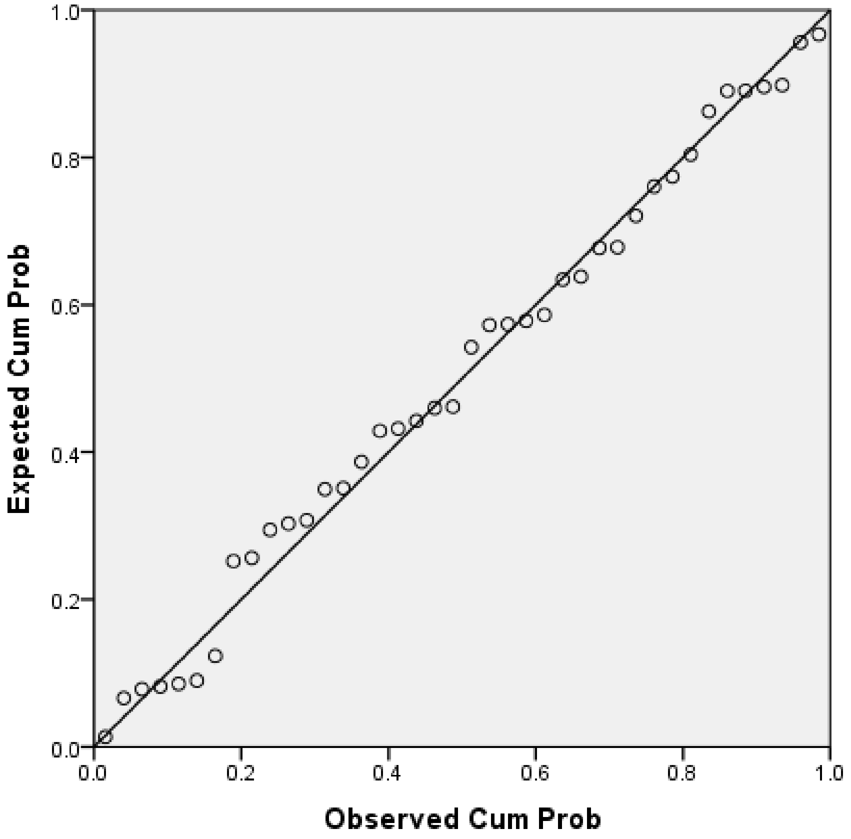


**Normal P-P Plot of Regression Standardized Residual.**

The dependent variable is the mean of pain intensity ratings.

**Figure S27.**

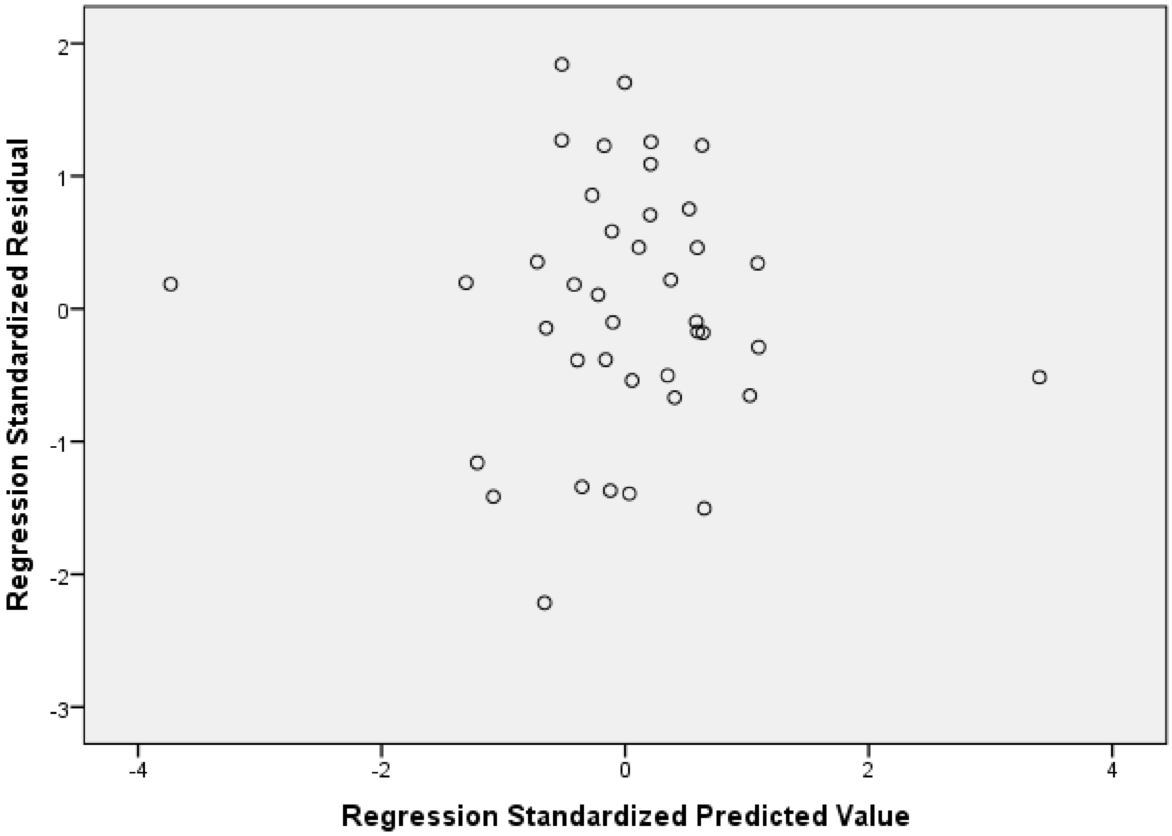


**Scatterplot.**

The dependent variable is the mean of pain intensity ratings.

Table S13. The Tolerance and the Variance Inflation Factor for Each Predictor Variable.

| Steps | Variables | Collinearity Statistics | |
|---|---|---|---|
| | | Tolerance | VIF |
| 1 | Occipital θ | 0.965 | 1.037 |
| | Trait Mindfulness | 0.965 | 1.037 |
| 2 | Occipital θ | 0.956 | 1.046 |
| | Trait Mindfulness | 0.839 | 1.192 |
| | Sex | 0.870 | 1.150 |
| 3 | Occipital θ | 0.661 | 1.513 |
| | Trait Mindfulness | 0.839 | 1.193 |
| | Sex | 0.779 | 1.283 |
| | Occipital θXTrait Mindfulness | 0.620 | 1.612 |

The dependent variable is the mean of pain intensity ratings. All continuous independent variables were centered (original values minus mean) before being entered into the models.

*2.10 Model 10.*

As shown in Figure S28 and Figure S29, the normality of residuals was evaluated visually using a histogram of standardized residuals and a probability-probability plot (P-P plot). Both plots indicated that the residuals approximately followed a normal distribution.

As shown in Figure S30, variance homogeneity was examined through a scatterplot of standardized residuals against predicted values. The residual plot showed no discernible pattern, suggesting constant variance across levels of the predictor variables.

Autocorrelation was assessed using the Durbin-Watson statistic, with values = 1.633, near 2, indicating no serious first-order autocorrelation.

As shown in Table S14, multicollinearity was evaluated by calculating both the tolerance and the variance inflation factor (VIF) for each predictor variable. All tolerance values were above 0.1, and all VIF values were well below the threshold of 10, indicating a low risk of multicollinearity.

A casewise diagnostic was also performed, identifying observations with standardized residuals exceeding three standard deviations to detect influential outliers; no such cases were found, suggesting the model is robust and not unduly affected by extreme values. Together, these diagnostics confirm that the model satisfies the fundamental assumptions, supporting the reliability of the regression estimates.

**Figure S28.**

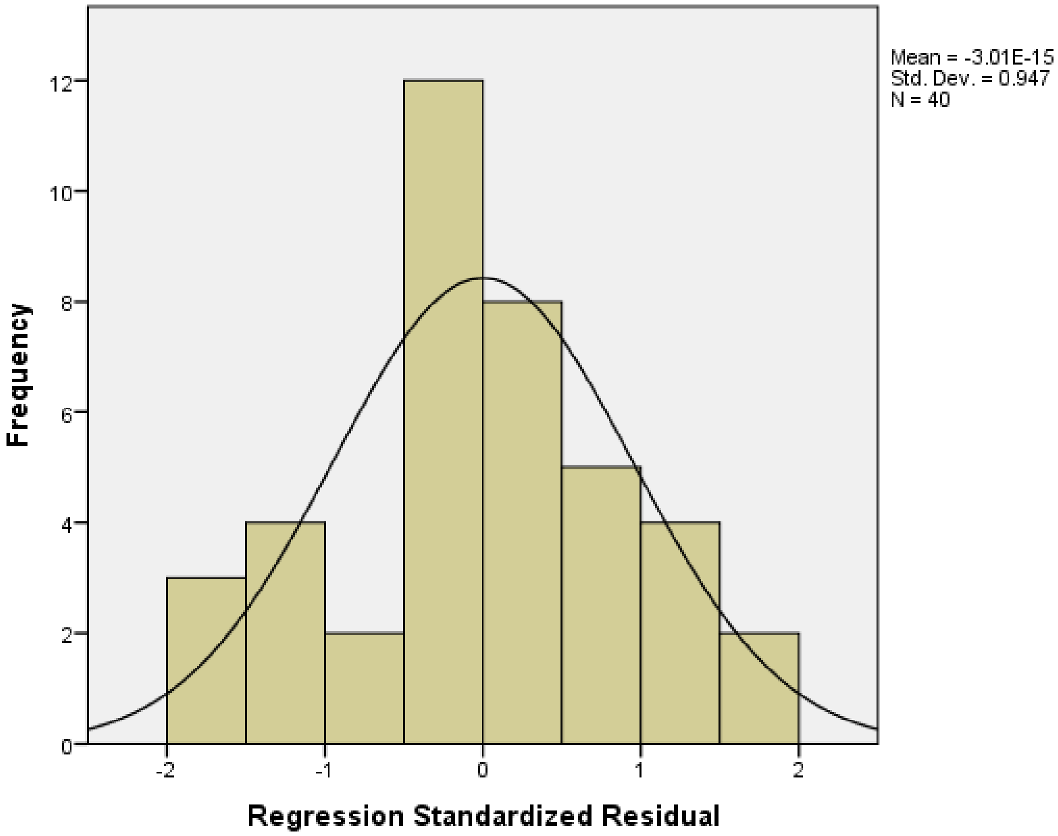


**Histogram of Standardized Residuals**

The dependent variable is the mean of pain intensity ratings.

**Figure S29.**

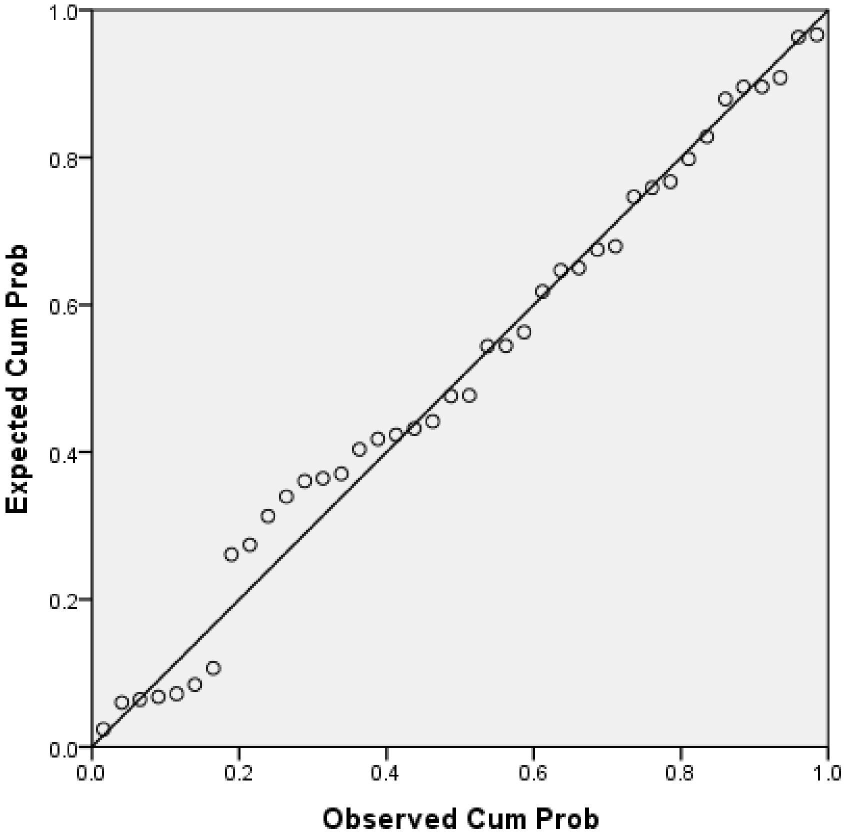


**Normal P-P Plot of Regression Standardized Residual.**

The dependent variable is the mean of pain intensity ratings.

**Figure S30.**

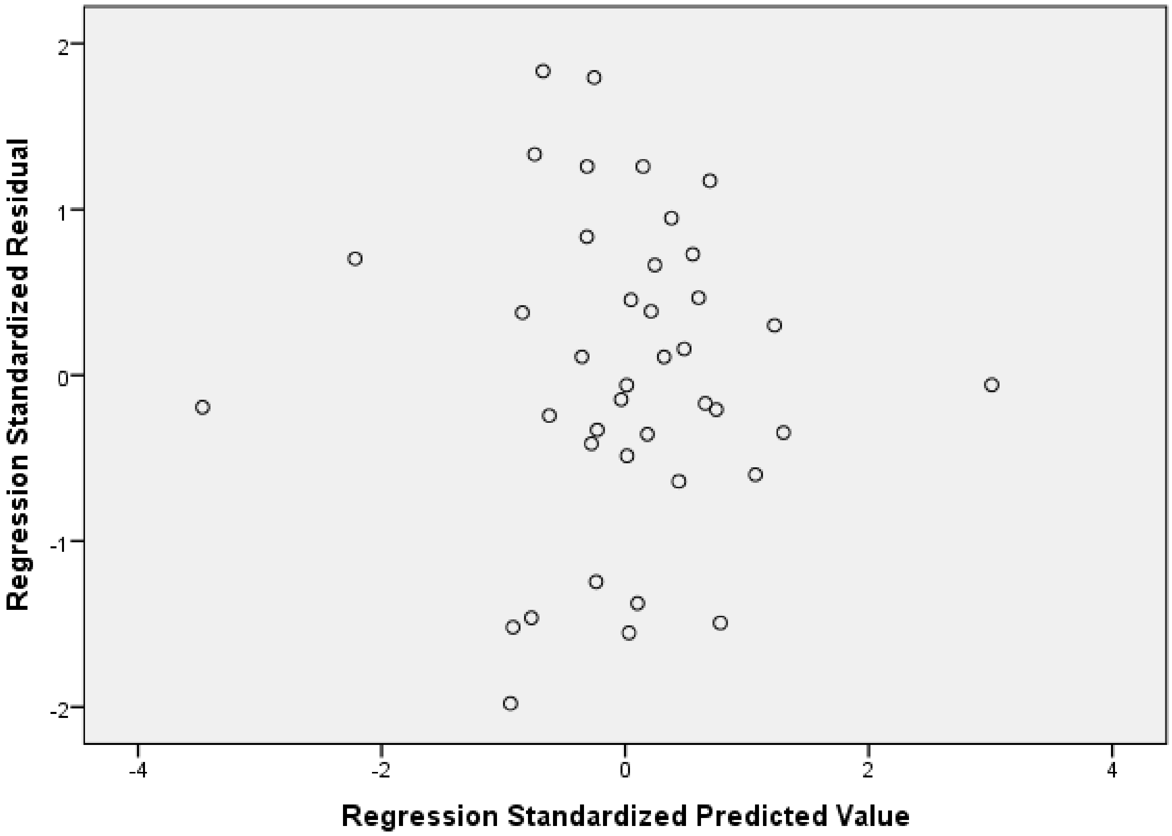


**Scatterplot.**

The dependent variable is the mean of pain intensity ratings.

Table S14. The Tolerance and the Variance Inflation Factor for Each Predictor Variable.

| Steps | Variables | Collinearity Statistics | |
|---|---|---|---|
| | | Tolerance | VIF |
| 1 | Temporal θ | 0.966 | 1.035 |
| | Trait Mindfulness | 0.966 | 1.035 |
| 2 | Temporal θ | 0.959 | 1.043 |
| | Trait Mindfulness | 0.842 | 1.188 |
| | Sex | 0.872 | 1.147 |
| 3 | Temporal θ | 0.763 | 1.310 |
| | Trait Mindfulness | 0.824 | 1.213 |
| | Sex | 0.756 | 1.323 |
| | Temporal θXTrait Mindfulness | 0.686 | 1.458 |

The dependent variable is the mean of pain intensity ratings. All continuous independent variables were centered (original values minus mean) before being entered into the models.

*2.11 Model 11.*

As shown in Figure S33 and Figure S34, the normality of residuals was evaluated visually using a histogram of standardized residuals and a probability-probability plot (P-P plot). Both plots indicated that the residuals approximately followed a normal distribution.

As shown in Figure S35, variance homogeneity was examined through a scatterplot of standardized residuals against predicted values. The residual plot showed no discernible pattern, suggesting constant variance across levels of the predictor variables.

Autocorrelation was assessed using the Durbin-Watson statistic, with values = 1.342, near 2, indicating no serious first-order autocorrelation.

As shown in Table S15, multicollinearity was evaluated by calculating both the tolerance and the variance inflation factor (VIF) for each predictor variable. All tolerance values were above 0.1, and all VIF values were well below the threshold of 10, indicating a low risk of multicollinearity.

A casewise diagnostic was also performed, identifying observations with standardized residuals exceeding three standard deviations to detect influential outliers; no such cases were found, suggesting the model is robust and not unduly affected by extreme values. Together, these diagnostics confirm that the model satisfies the fundamental assumptions, supporting the reliability of the regression estimates.

**Figure S31.**

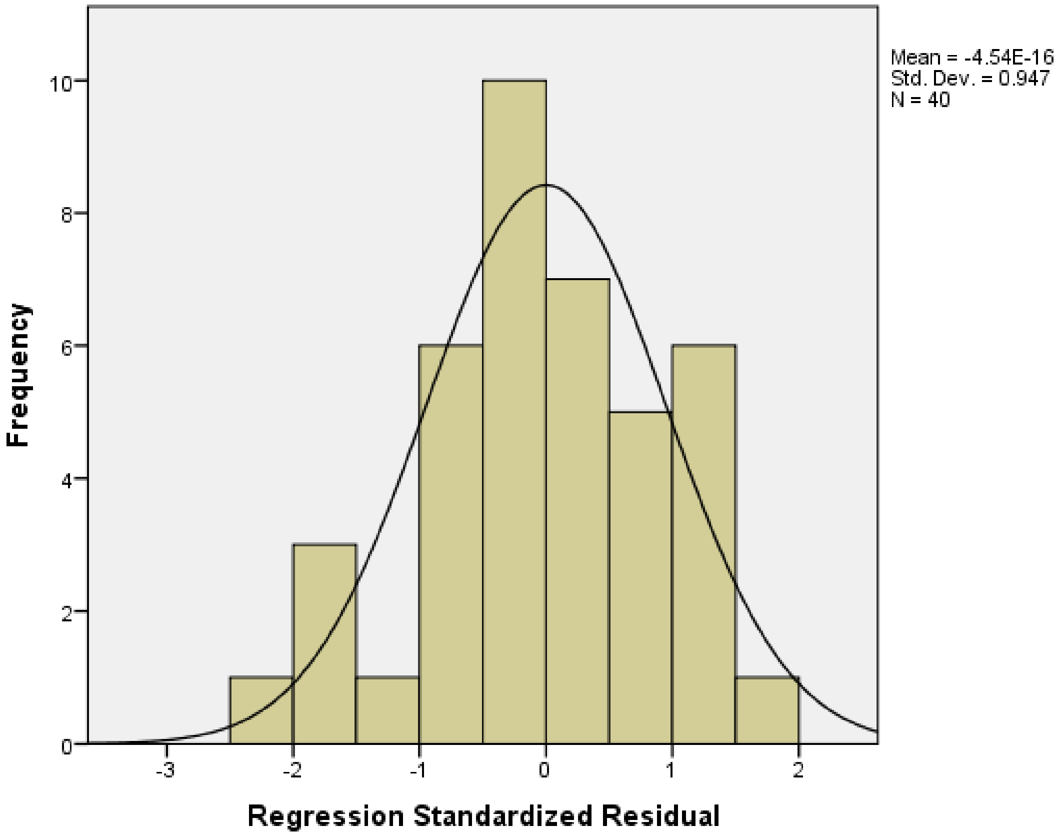


**Histogram of Standardized Residuals**

The dependent variable is the mean of pain intensity ratings.

**Figure S32.**

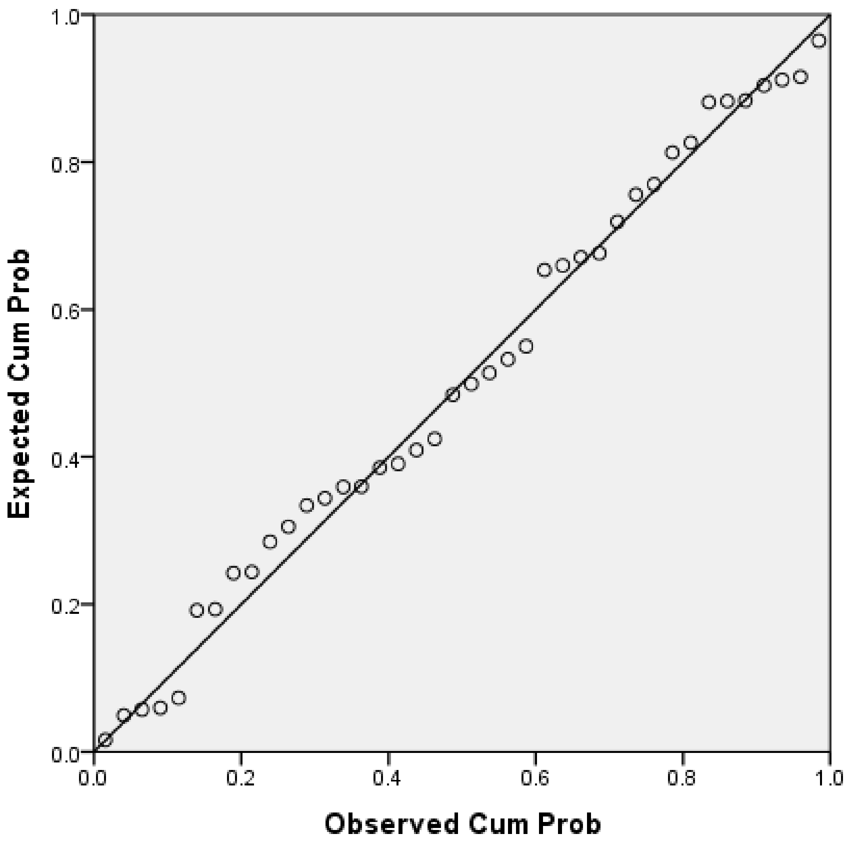


**Normal P-P Plot of Regression Standardized Residual.**

The dependent variable is the mean of pain intensity ratings.

**Figure S33.**

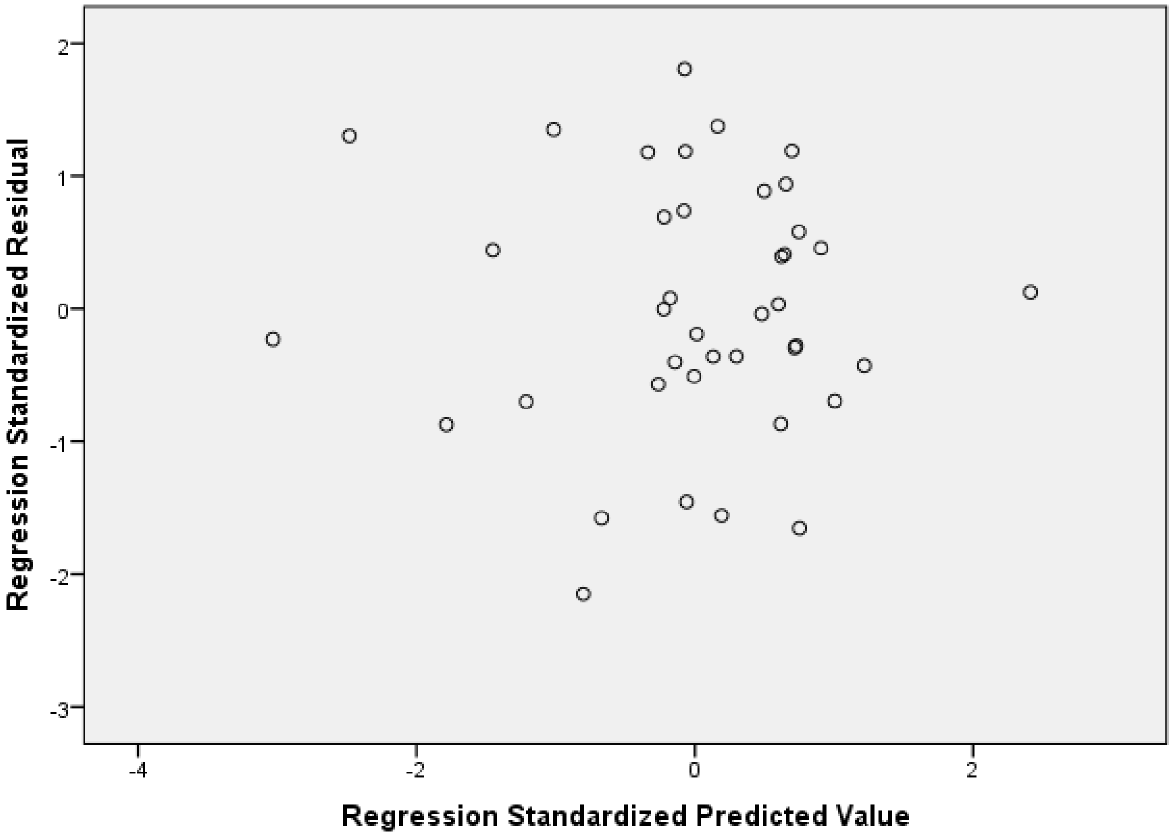


**Scatterplot.**

The dependent variable is the mean of pain intensity ratings.

Table S15. The Tolerance and the Variance Inflation Factor for Each Predictor Variable.

| Steps | Variables | Collinearity Statistics | |
|---|---|---|---|
| | | Tolerance | VIF |
| 1 | Frontal α | 0.983 | 1.018 |
| | Trait Mindfulness | 0.983 | 1.018 |
| 2 | Frontal α | 0.972 | 1.028 |
| | Trait Mindfulness | 0.871 | 1.148 |
| | Sex | 0.869 | 1.151 |
| 3 | Frontal α | 0.889 | 1.124 |
| | Trait Mindfulness | 0.869 | 1.151 |
| | Sex | 0.829 | 1.206 |
| | Frontal αXTrait Mindfulness | 0.886 | 1.129 |

The dependent variable is the mean of pain intensity ratings. All continuous independent variables were centered (original values minus mean) before being entered into the models.

*2.12 Model 12.*

As shown in Figure S34 and Figure S35, the normality of residuals was evaluated visually using a histogram of standardized residuals and a probability-probability plot (P-P plot). Both plots indicated that the residuals approximately followed a normal distribution.

As shown in Figure S36, variance homogeneity was examined through a scatterplot of standardized residuals against predicted values. The residual plot showed no discernible pattern, suggesting constant variance across levels of the predictor variables.

Autocorrelation was assessed using the Durbin-Watson statistic, with values = 1.425, near 2, indicating no serious first-order autocorrelation.

As shown in Table S16, multicollinearity was evaluated by calculating both the tolerance and the variance inflation factor (VIF) for each predictor variable. All tolerance values were above 0.1, and all VIF values were well below the threshold of 10, indicating a low risk of multicollinearity.

A casewise diagnostic was also performed, identifying observations with standardized residuals exceeding three standard deviations to detect influential outliers; no such cases were found, suggesting the model is robust and not unduly affected by extreme values. Together, these diagnostics confirm that the model satisfies the fundamental assumptions, supporting the reliability of the regression estimates.

**Figure S34.**

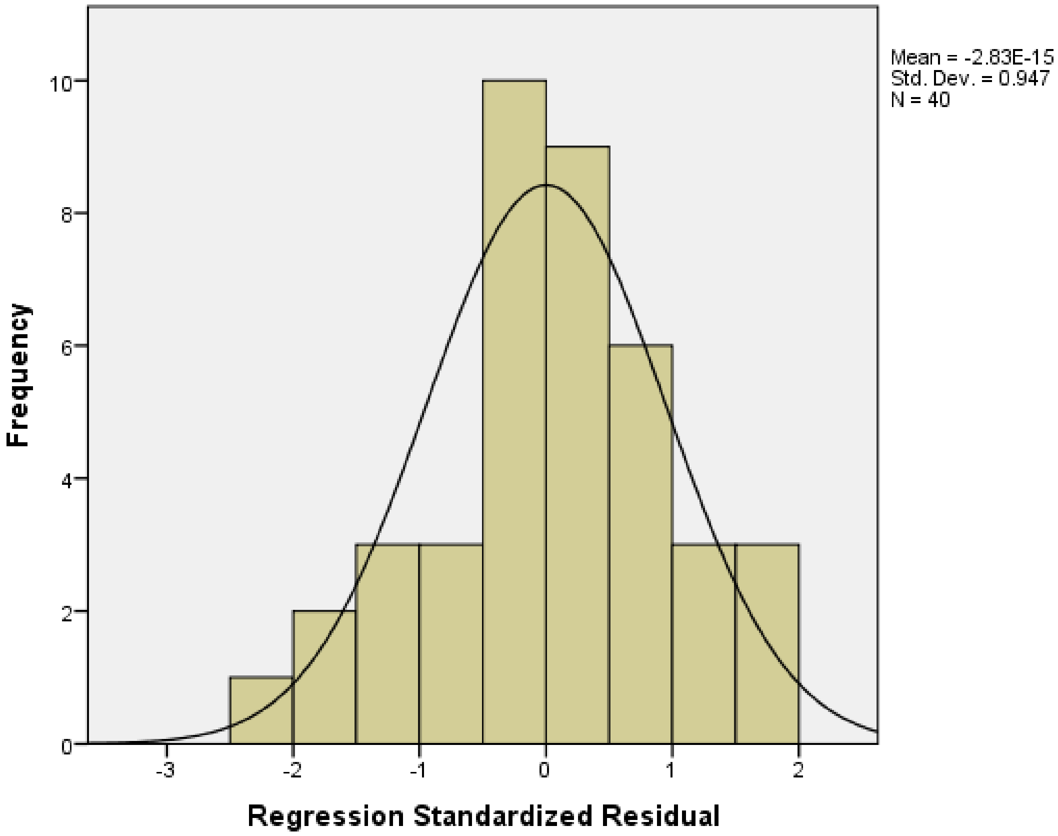


**Histogram of Standardized Residuals**

The dependent variable is the mean of pain intensity ratings.

**Figure S35.**

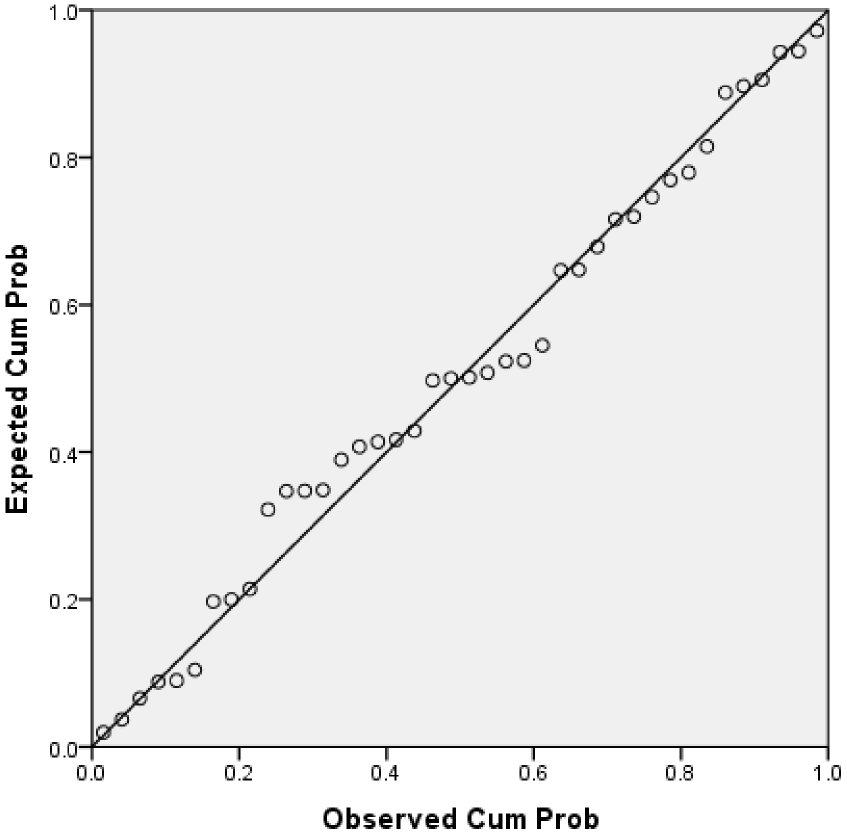


**Normal P-P Plot of Regression Standardized Residual.**

The dependent variable is the mean of pain intensity ratings.

**Figure S36.**

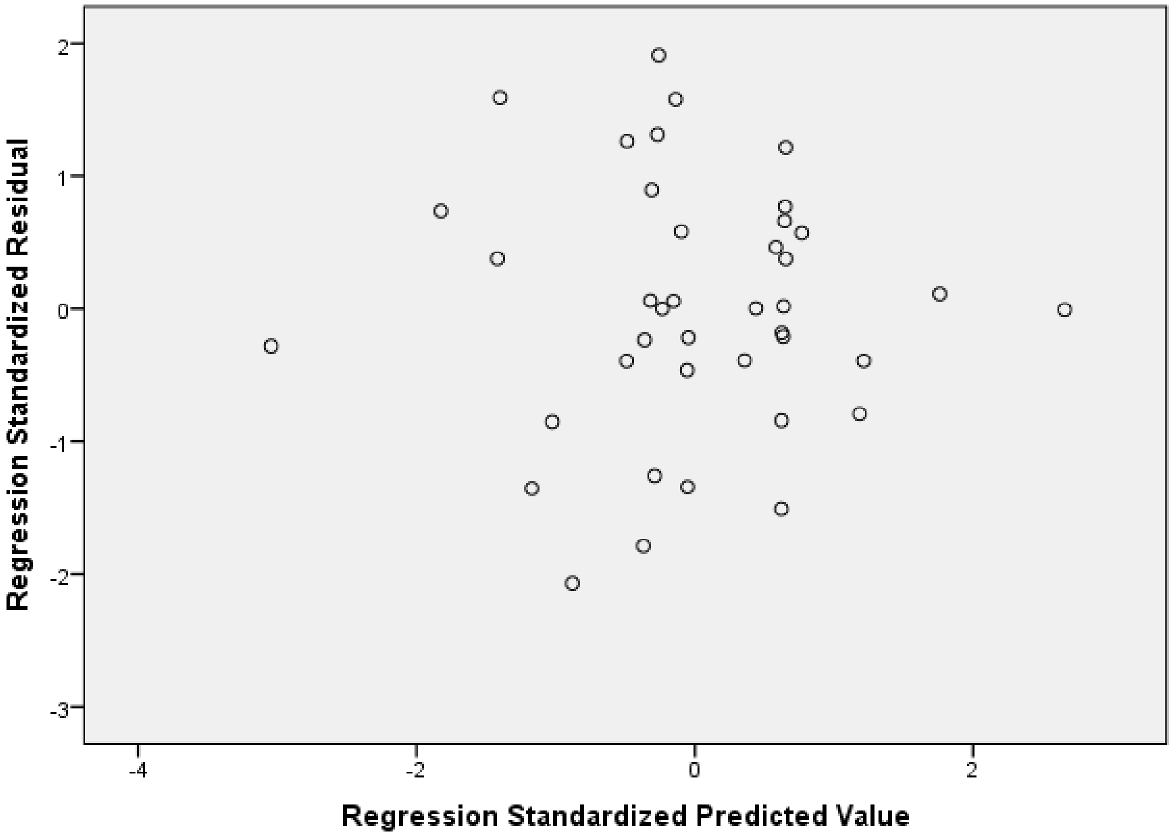


**Scatterplot.**

The dependent variable is the mean of pain intensity ratings.

Table S16. The Tolerance and the Variance Inflation Factor for Each Predictor Variable.

| Steps | Variables | Collinearity Statistics | |
|---|---|---|---|
| | | Tolerance | VIF |
| 1 | Central α | 0.984 | 1.017 |
| | Trait Mindfulness | 0.984 | 1.017 |
| 2 | Central α | 0.972 | 1.029 |
| | Trait Mindfulness | 0.872 | 1.147 |
| | Sex | 0.867 | 1.153 |
| 3 | Central α | 0.864 | 1.158 |
| | Trait Mindfulness | 0.870 | 1.149 |
| | Sex | 0.853 | 1.172 |
| | Central αXTrait Mindfulness | 0.881 | 1.135 |

The dependent variable is the mean of pain intensity ratings. All continuous independent variables were centered (original values minus mean) before being entered into the models.

*2.13 Model 13.*

As shown in Figure S37 and Figure S38, the normality of residuals was evaluated visually using a histogram of standardized residuals and a probability-probability plot (P-P plot). Both plots indicated that the residuals approximately followed a normal distribution.

As shown in Figure S39, variance homogeneity was examined through a scatterplot of standardized residuals against predicted values. The residual plot showed no discernible pattern, suggesting constant variance across levels of the predictor variables.

Autocorrelation was assessed using the Durbin-Watson statistic, with values = 1.329, near 2, indicating no serious first-order autocorrelation.

As shown in Table S17, multicollinearity was evaluated by calculating both the tolerance and the variance inflation factor (VIF) for each predictor variable. All tolerance values were above 0.1, and all VIF values were well below the threshold of 10, indicating a low risk of multicollinearity.

A casewise diagnostic was also performed, identifying observations with standardized residuals exceeding three standard deviations to detect influential outliers; no such cases were found, suggesting the model is robust and not unduly affected by extreme values. Together, these diagnostics confirm that the model satisfies the fundamental assumptions, supporting the reliability of the regression estimates.

**Figure S37.**

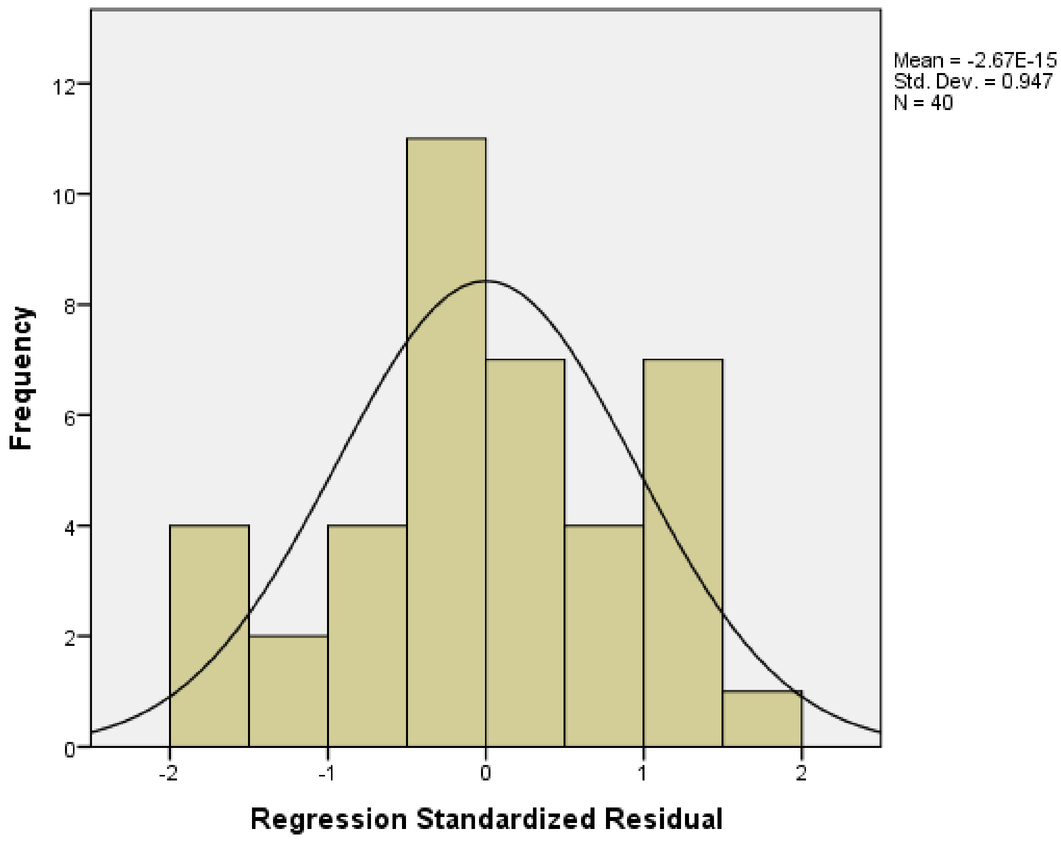


**Histogram of Standardized Residuals**

The dependent variable is the mean of pain intensity ratings.

**Figure S38.**

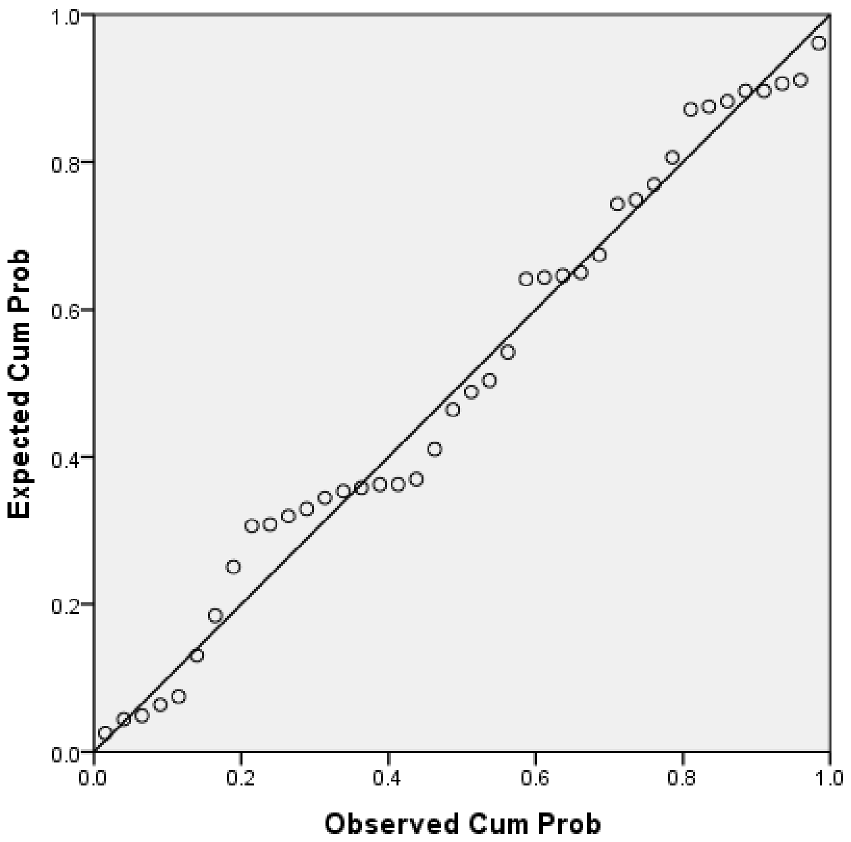


**Normal P-P Plot of Regression Standardized Residual.**

The dependent variable is the mean of pain intensity ratings.

**Figure S39.**

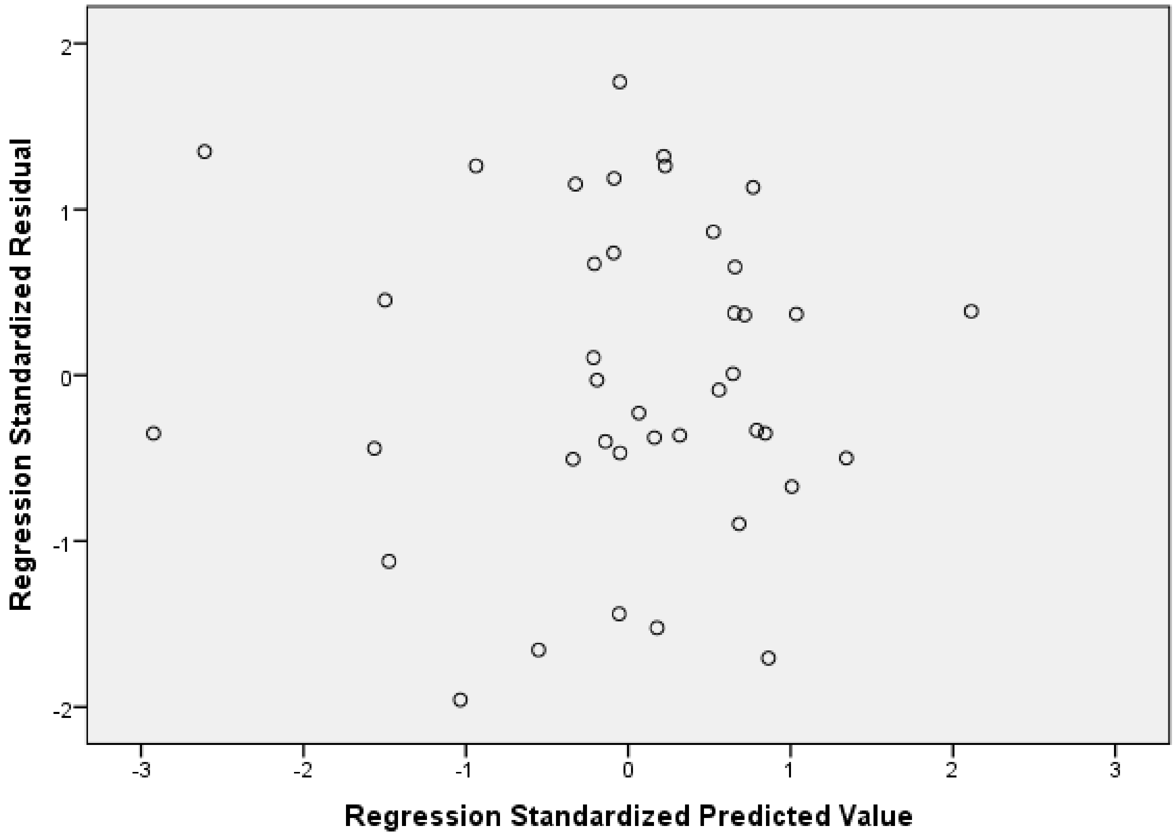


**Scatterplot.**
The dependent variable is the mean of pain intensity ratings.

Table S17. The Tolerance and the Variance Inflation Factor for Each Predictor Variable.

| | | Collinearity Statistics | |
|---|---|---|---|
| Steps | Variables | Tolerance | VIF |
| 1 | Parietal α | 0.999 | 1.001 |
| | C_MAAS | 0.999 | 1.001 |
| 2 | Parietal α | 0.996 | 1.004 |
| | C_MAAS | 0.878 | 1.139 |
| | Sex | 0.875 | 1.142 |
| 3 | Parietal α | 0.986 | 1.015 |
| | C_MAAS | 0.876 | 1.141 |
| | Sex | 0.828 | 1.207 |
| | Parietal αXC_MAAS | 0.937 | 1.068 |

The dependent variable is the mean of pain intensity ratings. All continuous independent variables were centered (original values minus mean) before being entered into the models.

*2.14 Model 14.*

As shown in Figure S40 and Figure S41, the normality of residuals was evaluated visually using a histogram of standardized residuals and a probability-probability plot (P-P plot). Both plots indicated that the residuals approximately followed a normal distribution.

As shown in Figure S42, variance homogeneity was examined through a scatterplot of standardized residuals against predicted values. The residual plot showed no discernible pattern, suggesting constant variance across levels of the predictor variables.

Autocorrelation was assessed using the Durbin-Watson statistic, with values = 1.325, near 2, indicating no serious first-order autocorrelation.

As shown in Table S18, multicollinearity was evaluated by calculating both the tolerance and the variance inflation factor (VIF) for each predictor variable. All tolerance values were above 0.1, and all VIF values were well below the threshold of 10, indicating a low risk of multicollinearity.

A casewise diagnostic was also performed, identifying observations with standardized residuals exceeding three standard deviations to detect influential outliers; no such cases were found, suggesting the model is robust and not unduly affected by extreme values. Together, these diagnostics confirm that the model satisfies the fundamental assumptions, supporting the reliability of the regression estimates.

**Figure S40.**

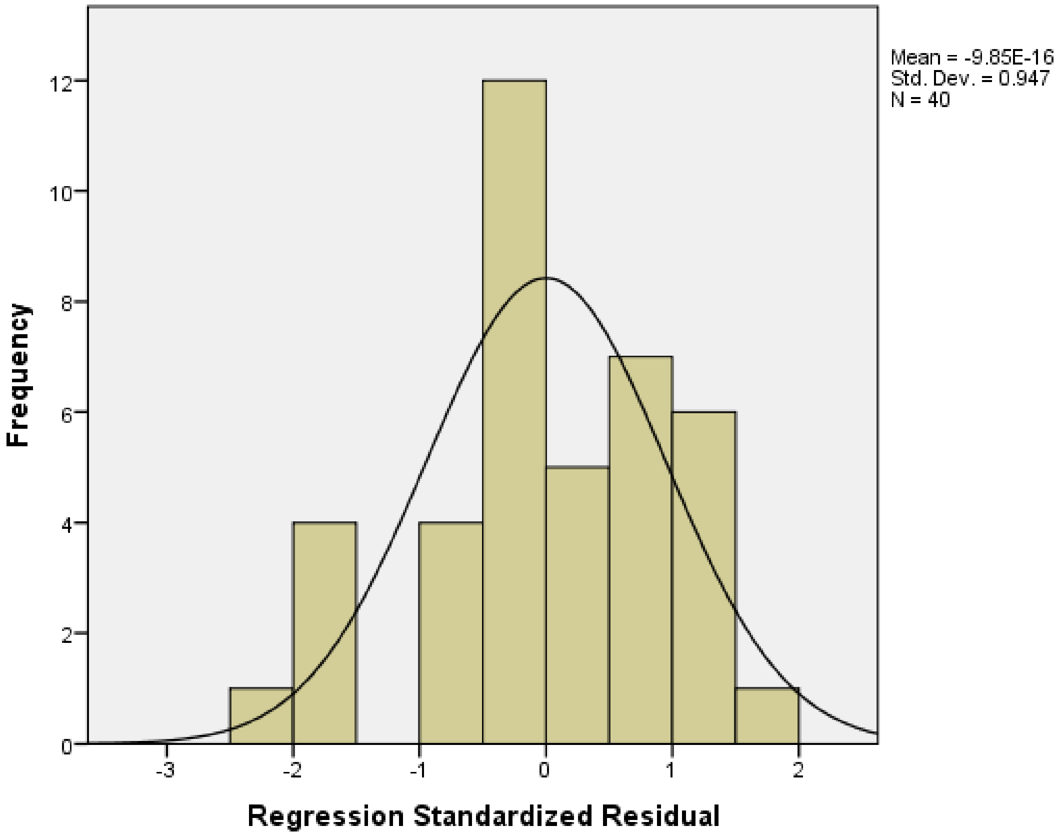


**Histogram of Standardized Residuals**

The dependent variable is the mean of pain intensity ratings.

**Figure S41.**

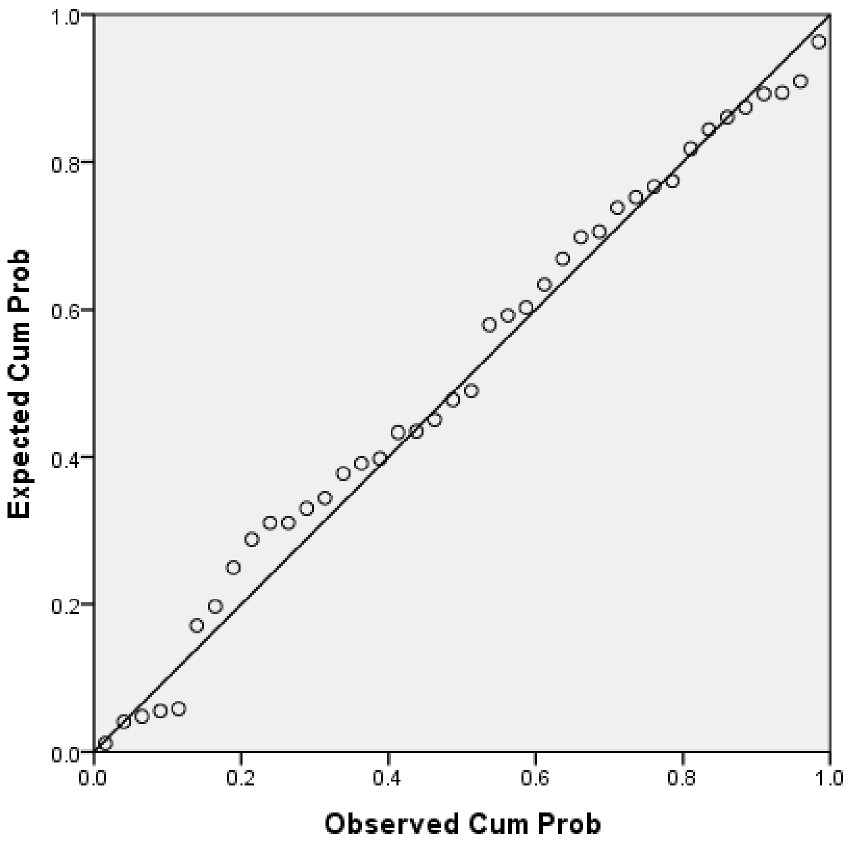


**Normal P-P Plot of Regression Standardized Residual.**

The dependent variable is the mean of pain intensity ratings.

**Figure S42.**

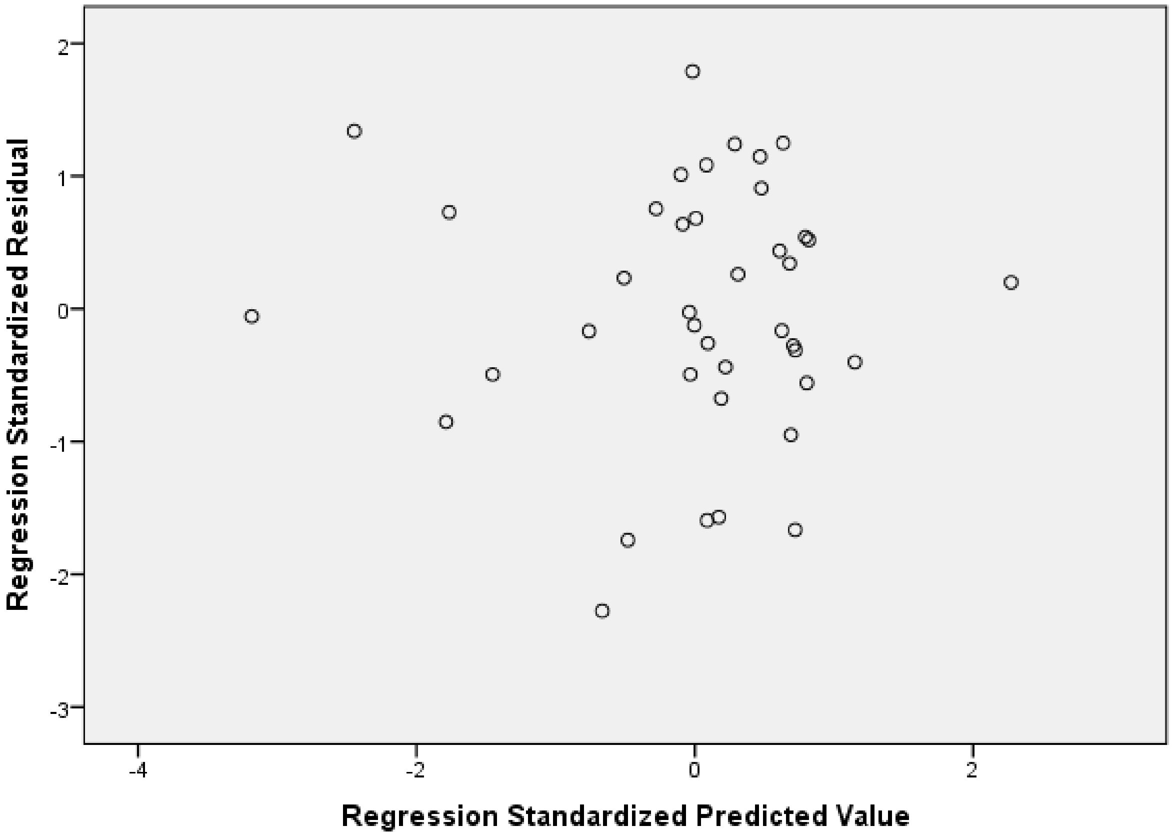


**Scatterplot.**
The dependent variable is the mean of pain intensity ratings.

Table S18. The Tolerance and the Variance Inflation Factor for Each Predictor Variable.

| | | Collinearity Statistics | |
|---|---|---|---|
| Steps | Variables | Tolerance | VIF |
| 1 | Occipital α | 0.988 | 1.012 |
| | Trait Mindfulness | 0.988 | 1.012 |
| 2 | Occipital α | 0.977 | 1.023 |
| | Trait Mindfulness | 0.874 | 1.144 |
| | Sex | 0.868 | 1.152 |
| 3 | Occipital α | 0.859 | 1.164 |
| | Trait Mindfulness | 0.859 | 1.164 |
| | Sex | 0.799 | 1.251 |
| | Occipital αXTrait Mindfulness | 0.828 | 1.208 |

The dependent variable is the mean of pain intensity ratings. All continuous independent variables were centered (original values minus mean) before being entered into the models.

*2.15 Model 15.*

As shown in Figure S43 and Figure S44, the normality of residuals was evaluated visually using a histogram of standardized residuals and a probability-probability plot (P-P plot). Both plots indicated that the residuals approximately followed a normal distribution.

As shown in Figure S45, variance homogeneity was examined through a scatterplot of standardized residuals against predicted values. The residual plot showed no discernible pattern, suggesting constant variance across levels of the predictor variables.

Autocorrelation was assessed using the Durbin-Watson statistic, with values = 1.319, near 2, indicating no serious first-order autocorrelation.

As shown in Table S19, multicollinearity was evaluated by calculating both the tolerance and the variance inflation factor (VIF) for each predictor variable. All tolerance values were above 0.1, and all VIF values were well below the threshold of 10, indicating a low risk of multicollinearity.

A casewise diagnostic was also performed, identifying observations with standardized residuals exceeding three standard deviations to detect influential outliers; no such cases were found, suggesting the model is robust and not unduly affected by extreme values. Together, these diagnostics confirm that the model satisfies the fundamental assumptions, supporting the reliability of the regression estimates.

**Figure S43.**

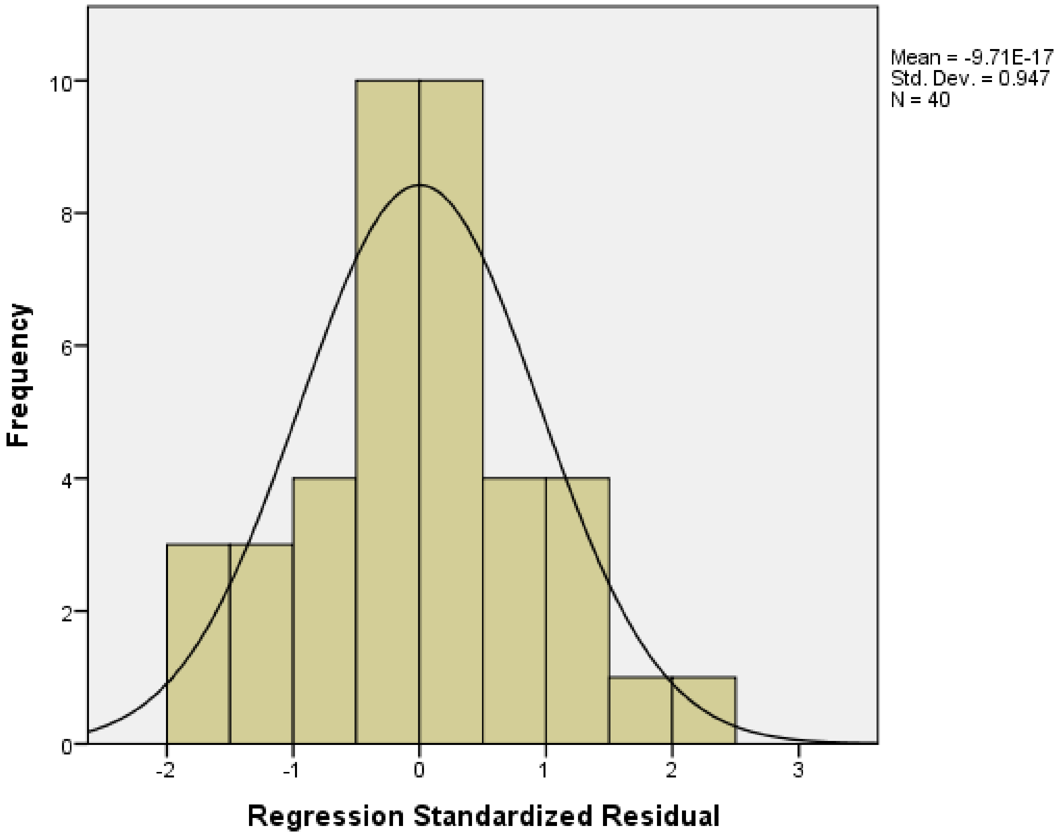


**Histogram of Standardized Residuals**

The dependent variable is the mean of pain intensity ratings.

**Figure S44.**

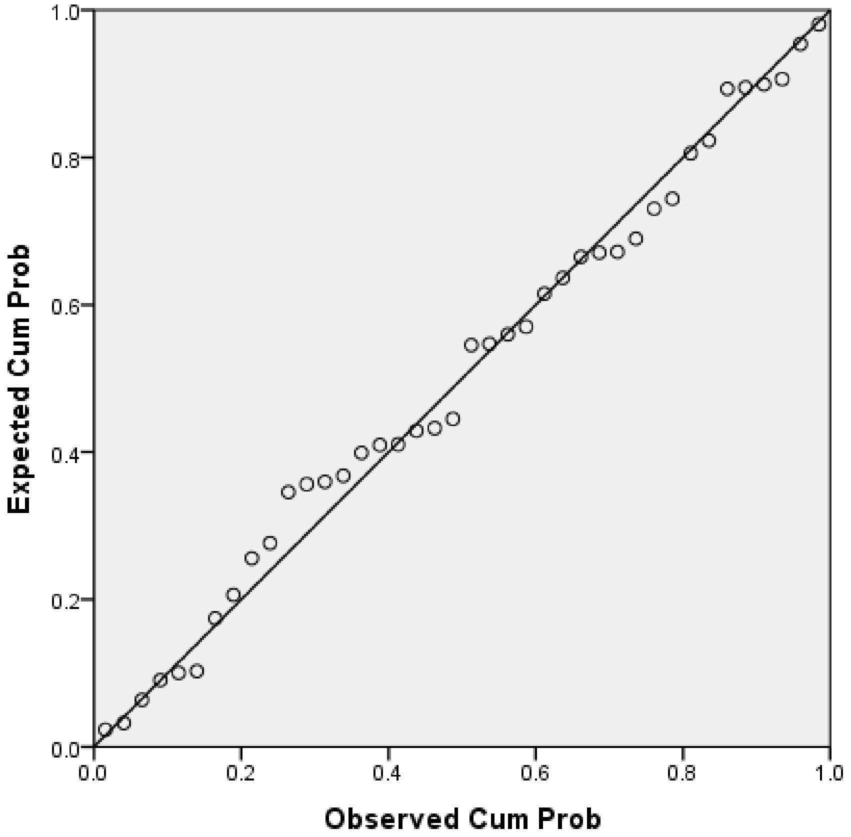


**Normal P-P Plot of Regression Standardized Residual.**

The dependent variable is the mean of pain intensity ratings.

**Figure S45.**

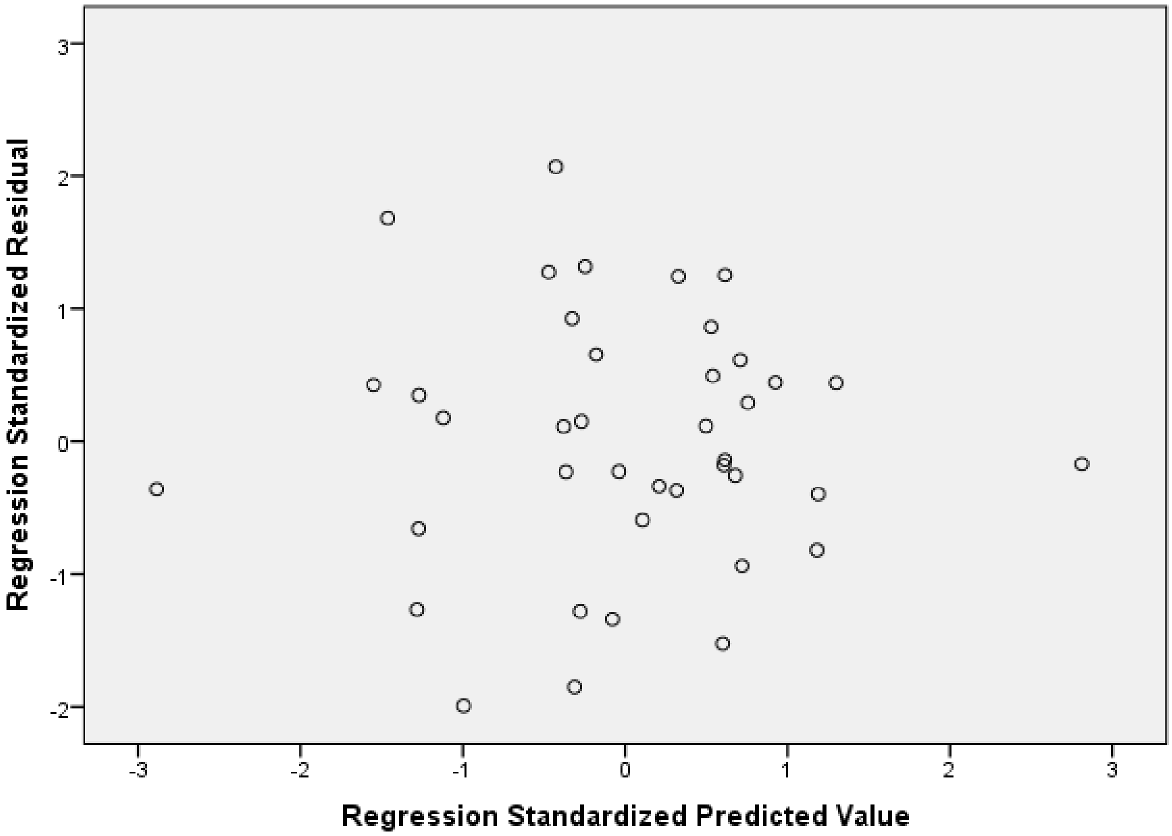


**Scatterplot.**
The dependent variable is the mean of pain intensity ratings.

Table S19. The Tolerance and the Variance Inflation Factor for Each Predictor Variable.

| | | Collinearity Statistics | |
|---|---|---|---|
| Steps | Variables | Tolerance | VIF |
| 1 | Temporal α | 0.961 | 1.040 |
| | Trait Mindfulness | 0.961 | 1.040 |
| 2 | Temporal α | 0.961 | 1.041 |
| | Trait Mindfulness | 0.850 | 1.176 |
| | Sex | 0.877 | 1.140 |
| 3 | Temporal α | 0.791 | 1.265 |
| | Trait Mindfulness | 0.842 | 1.187 |
| | Sex | 0.862 | 1.160 |
| | Temporal αXTrait Mindfulness | 0.799 | 1.252 |

The dependent variable is the mean of pain intensity ratings. All continuous independent variables were centered (original values minus mean) before being entered into the models.

*2.16 Model 16.*

As shown in Figure S46 and Figure S47, the normality of residuals was evaluated visually using a histogram of standardized residuals and a probability-probability plot (P-P plot). Both plots indicated that the residuals approximately followed a normal distribution.

As shown in Figure S48, variance homogeneity was examined through a scatterplot of standardized residuals against predicted values. The residual plot showed no discernible pattern, suggesting constant variance across levels of the predictor variables.

Autocorrelation was assessed using the Durbin-Watson statistic, with values = 1.943, near 2, indicating no significant first-order autocorrelation.

As shown in Table S20, multicollinearity was evaluated by calculating both the tolerance and the variance inflation factor (VIF) for each predictor variable. All tolerance values were above 0.1, and all VIF values were well below the threshold of 10, indicating a low risk of multicollinearity.

A casewise diagnostic was also performed, identifying observations with standardized residuals exceeding three standard deviations to detect influential outliers; no such cases were found, suggesting the model is robust and not unduly affected by extreme values. Together, these diagnostics confirm that the model satisfies the fundamental assumptions, supporting the reliability of the regression estimates.

**Figure S46.**

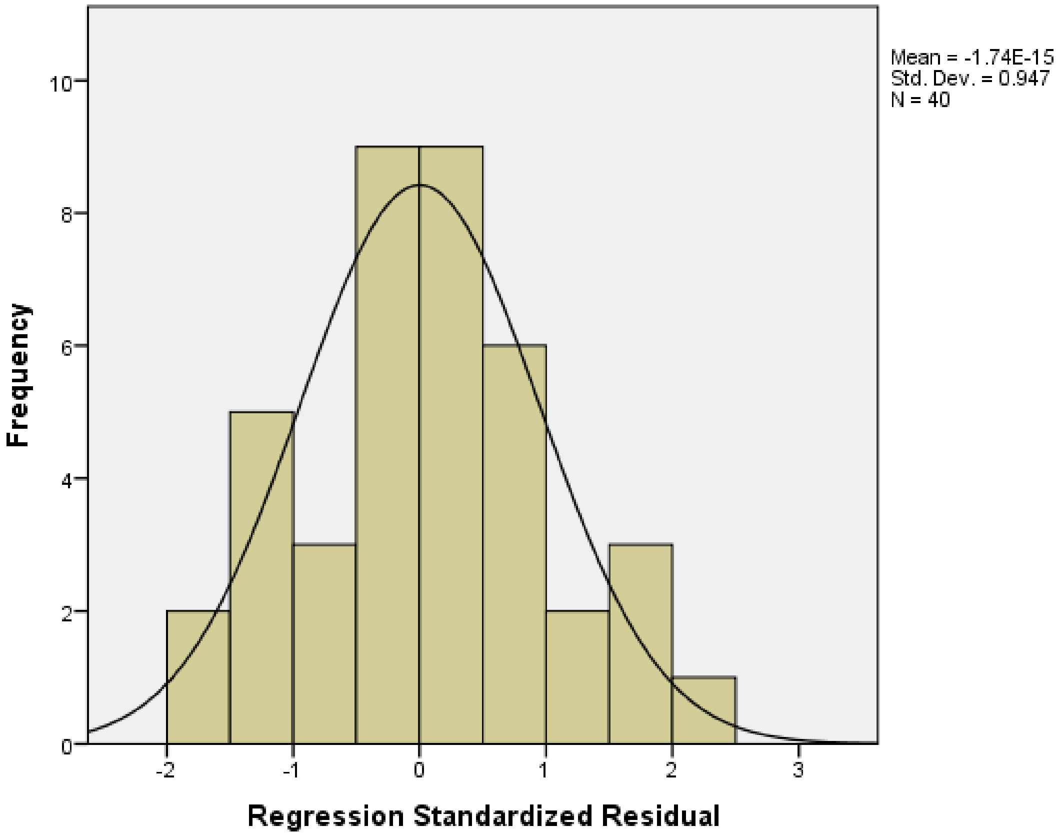


**Histogram of Standardized Residuals**

The dependent variable is the mean of pain intensity ratings.

**Figure S47.**

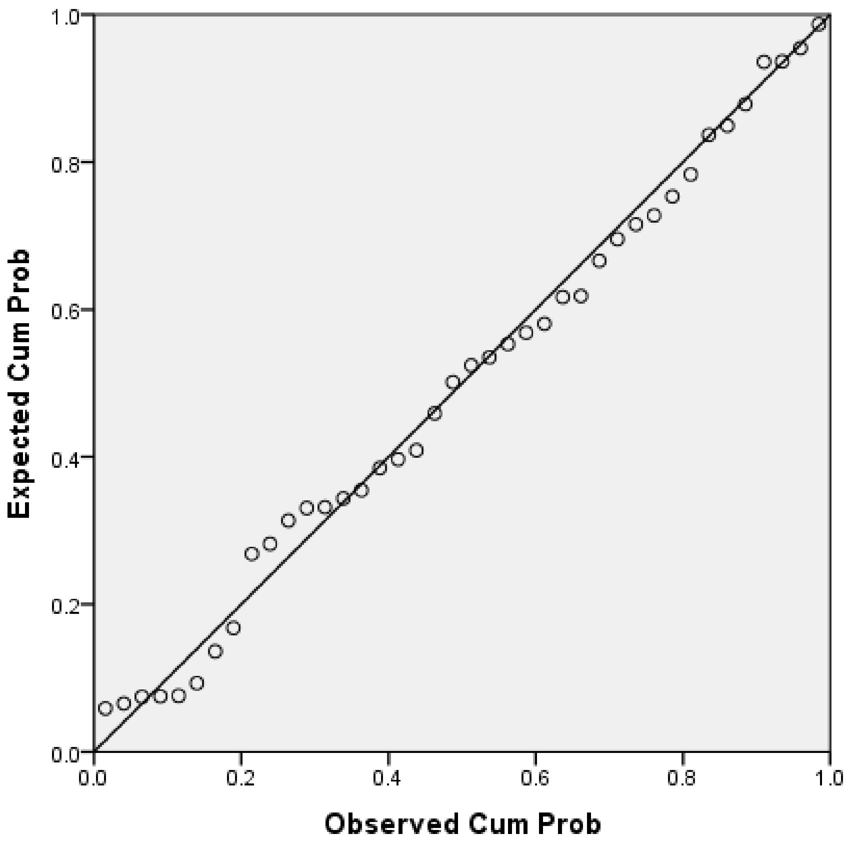


**Normal P-P Plot of Regression Standardized Residual.**

The dependent variable is the mean of pain intensity ratings.

**Figure S48.**

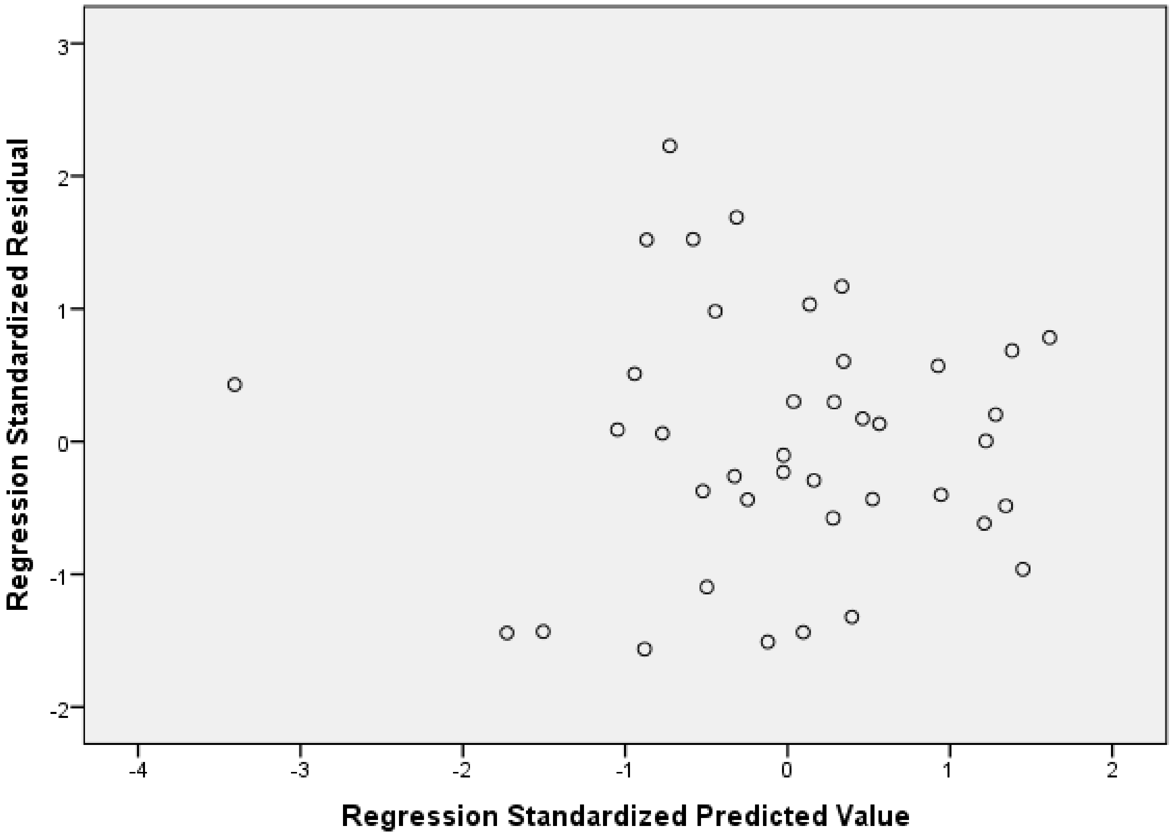


**Scatterplot.**
The dependent variable is the mean of pain intensity ratings.

Table S20. The Tolerance and the Variance Inflation Factor for Each Predictor Variable.

| | | Collinearity Statistics | |
|---|---|---|---|
| Steps | Variables | Tolerance | VIF |
| 1 | Frontal β | 0.994 | 1.007 |
| | Trait Mindfulness | 0.994 | 1.007 |
| 2 | Frontal β | 0.880 | 1.137 |
| | Trait Mindfulness | 0.876 | 1.142 |
| | Sex | 0.777 | 1.287 |
| 3 | Frontal β | 0.764 | 1.309 |
| | Trait Mindfulness | 0.818 | 1.223 |
| | Sex | 0.688 | 1.454 |
| | Frontal βXTrait Mindfulness | 0.806 | 1.241 |

The dependent variable is the mean of pain intensity ratings. All continuous independent variables were centered (original values minus mean) before being entered into the models.

*2.17 Model 17.*

As shown in Figure S49 and Figure S50, the normality of residuals was evaluated visually using a histogram of standardized residuals and a probability-probability plot (P-P plot). Both plots indicated that the residuals approximately followed a normal distribution.

As shown in Figure S51, variance homogeneity was examined through a scatterplot of standardized residuals against predicted values. The residual plot showed no discernible pattern, suggesting constant variance across levels of the predictor variables.

Autocorrelation was assessed using the Durbin-Watson statistic, with values = 1.707, near 2, indicating no significant first-order autocorrelation.

As shown in Table S21, multicollinearity was evaluated by calculating both the tolerance and the variance inflation factor (VIF) for each predictor variable. All tolerance values were above 0.1, and all VIF values were well below the threshold of 10, indicating a low risk of multicollinearity.

A casewise diagnostic was also performed, identifying observations with standardized residuals exceeding three standard deviations to detect influential outliers; no such cases were found, suggesting the model is robust and not unduly affected by extreme values. Together, these diagnostics confirm that the model satisfies the fundamental assumptions, supporting the reliability of the regression estimates.

**Figure S49.**

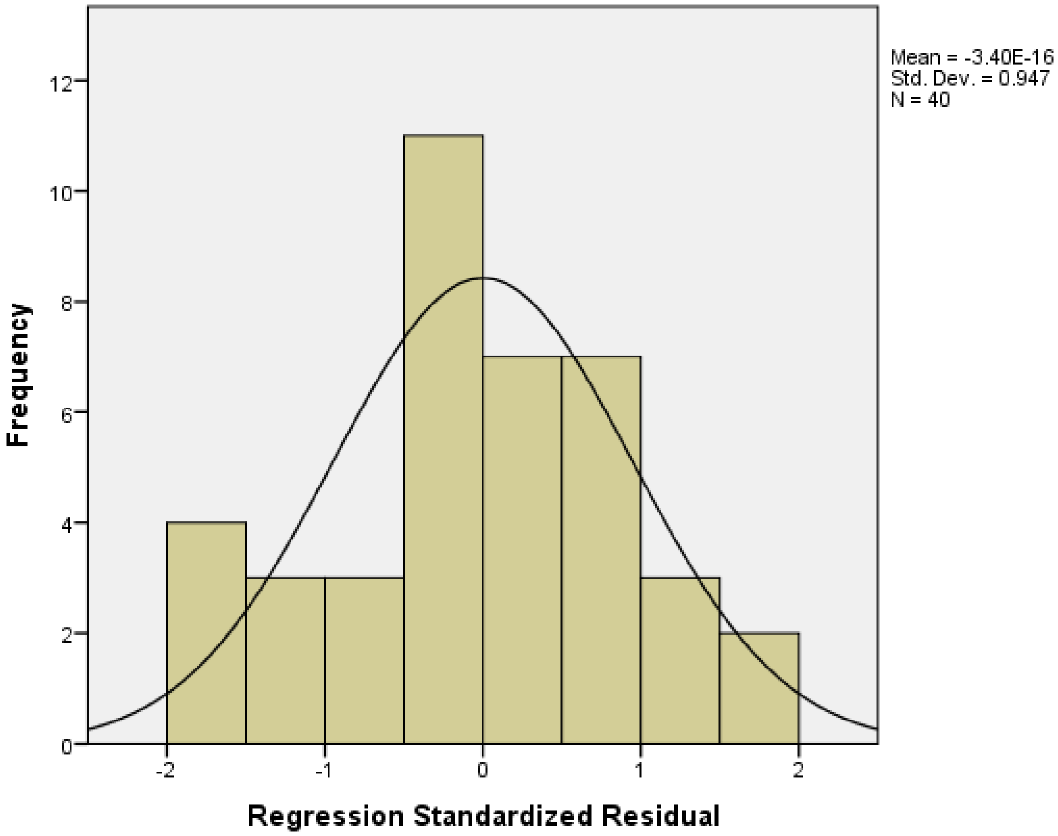


**Histogram of Standardized Residuals**

The dependent variable is the mean of pain intensity ratings.

**Figure S50.**

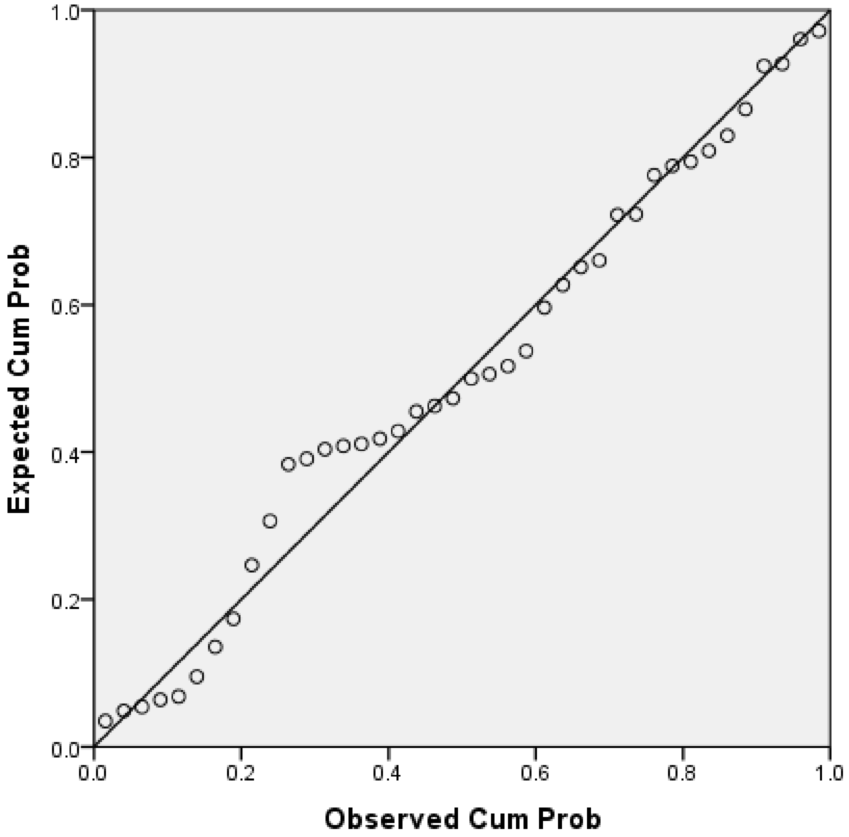


**Normal P-P Plot of Regression Standardized Residual.**

The dependent variable is the mean of pain intensity ratings.

**Figure S51.**

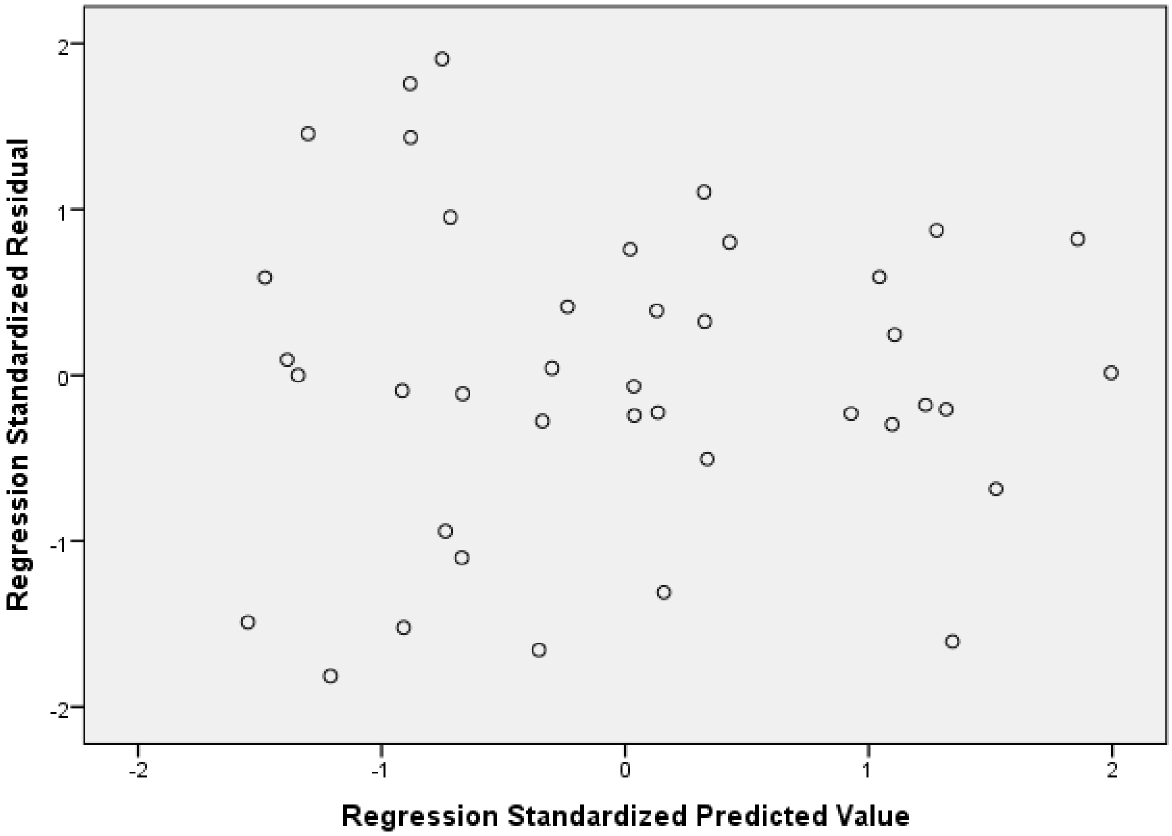


**Scatterplot.**
The dependent variable is the mean of pain intensity ratings.

Table S21. The Tolerance and the Variance Inflation Factor for Each Predictor Variable.

| | | Collinearity Statistics | |
|---|---|---|---|
| Steps | Variables | Tolerance | VIF |
| 1 | Central β | 0.999 | 1.001 |
| | Trait Mindfulness | 0.999 | 1.001 |
| 2 | Central β | 0.931 | 1.074 |
| | Trait Mindfulness | 0.863 | 1.159 |
| | Sex | 0.818 | 1.222 |
| 3 | Central β | 0.853 | 1.172 |
| | Trait Mindfulness | 0.797 | 1.255 |
| | Sex | 0.781 | 1.280 |
| | Central βXTrait Mindfulness | 0.805 | 1.243 |

The dependent variable is the mean of pain intensity ratings. All continuous independent variables were centered (original values minus mean) before being entered into the models.

*2.18 Model 18.*

As shown in Figure S52 and Figure S53, the normality of residuals was evaluated visually using a histogram of standardized residuals and a probability-probability plot (P-P plot). Both plots indicated that the residuals approximately followed a normal distribution.

As shown in Figure S54, variance homogeneity was examined through a scatterplot of standardized residuals against predicted values. The residual plot showed no discernible pattern, suggesting constant variance across levels of the predictor variables.

Autocorrelation was assessed using the Durbin-Watson statistic, with values = 1.714, near 2, indicating no significant first-order autocorrelation.

As shown in Table S22, multicollinearity was evaluated by calculating both the tolerance and the variance inflation factor (VIF) for each predictor variable. All tolerance values were above 0.1, and all VIF values were well below the threshold of 10, indicating a low risk of multicollinearity.

A casewise diagnostic was also performed, identifying observations with standardized residuals exceeding three standard deviations to detect influential outliers; no such cases were found, suggesting the model is robust and not unduly affected by extreme values. Together, these diagnostics confirm that the model satisfies the fundamental assumptions, supporting the reliability of the regression estimates.

**Figure S52.**

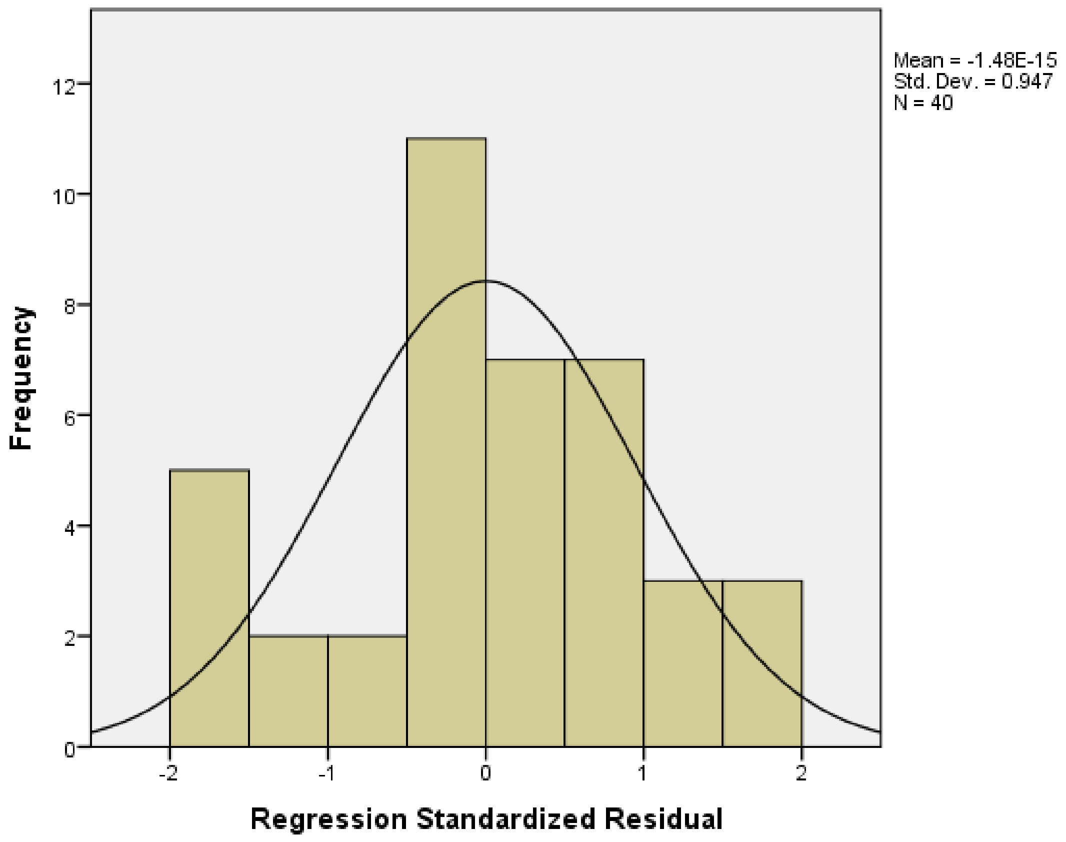


**Histogram of Standardized Residuals**

The dependent variable is the mean of pain intensity ratings.

**Figure S53.**

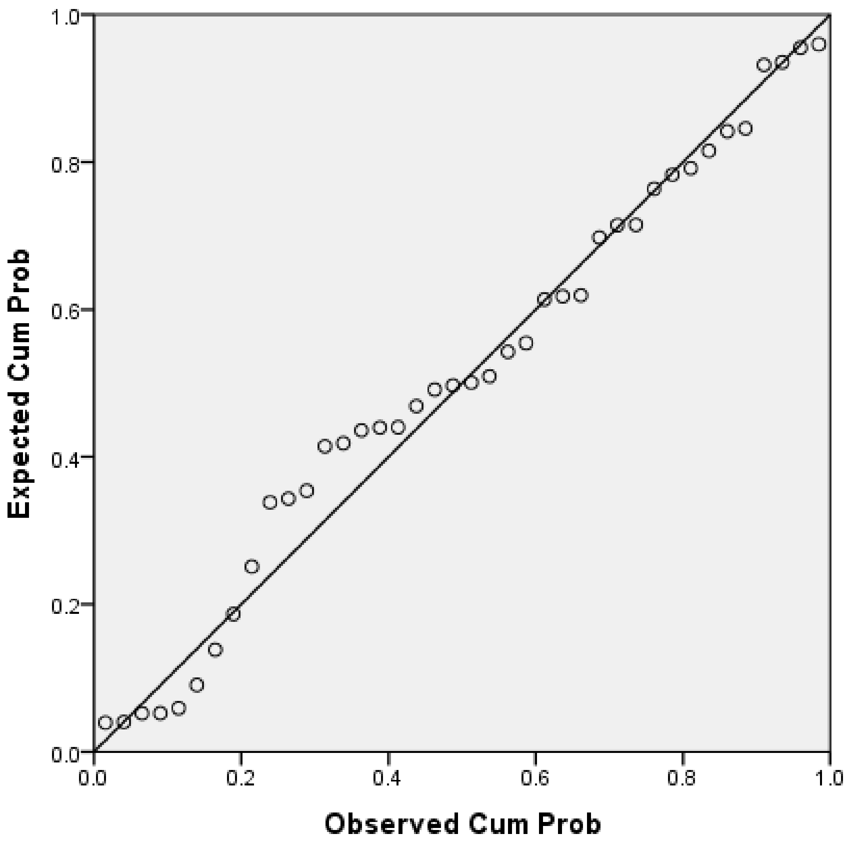


**Normal P-P Plot of Regression Standardized Residual.**
The dependent variable is the mean of pain intensity ratings.

**Figure S54.**

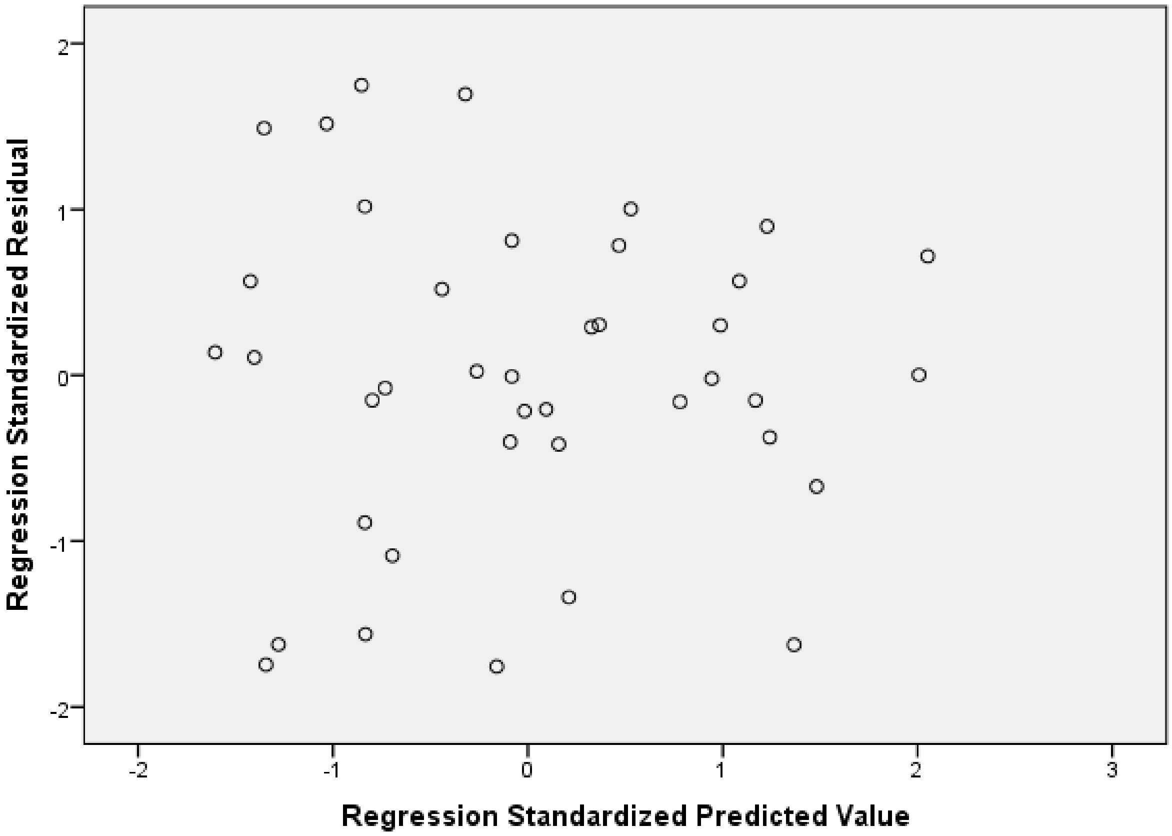


**Scatterplot.**
The dependent variable is the mean of pain intensity ratings.

Table S22. The Tolerance and the Variance Inflation Factor for Each Predictor Variable.

| | | Collinearity Statistics | |
|---|---|---|---|
| Steps | Variables | Tolerance | VIF |
| 1 | Parietal β | 0.990 | 1.011 |
| | Trait Mindfulness | 0.990 | 1.011 |
| 2 | Parietal β | 0.871 | 1.148 |
| | Trait Mindfulness | 0.833 | 1.200 |
| | Sex | 0.772 | 1.295 |
| 3 | Parietal β | 0.420 | 2.378 |
| | Trait Mindfulness | 0.818 | 1.222 |
| | Sex | 0.771 | 1.297 |
| | Parietal βXTrait Mindfulness | 0.432 | 2.315 |

The dependent variable is the mean of pain intensity ratings. All continuous independent variables were centered (original values minus mean) before being entered into the models.

*2.19 Model 19.*

As shown in Figure S55 and Figure S56, the normality of residuals was evaluated visually using a histogram of standardized residuals and a probability-probability plot (P-P plot). Both plots indicated that the residuals approximately followed a normal distribution.

As shown in Figure S57, variance homogeneity was examined through a scatterplot of standardized residuals against predicted values. The residual plot showed no discernible pattern, suggesting constant variance across levels of the predictor variables.

Autocorrelation was assessed using the Durbin-Watson statistic, with values = 1.705, near 2, indicating no significant first-order autocorrelation.

As shown in Table S23, multicollinearity was evaluated by calculating both the tolerance and the variance inflation factor (VIF) for each predictor variable. All tolerance values were above 0.1, and all VIF values were well below the threshold of 10, indicating a low risk of multicollinearity.

A casewise diagnostic was also performed, identifying observations with standardized residuals exceeding three standard deviations to detect influential outliers; no such cases were found, suggesting the model is robust and not unduly affected by extreme values. Together, these diagnostics confirm that the model satisfies the fundamental assumptions, supporting the reliability of the regression estimates.

**Figure S55.**

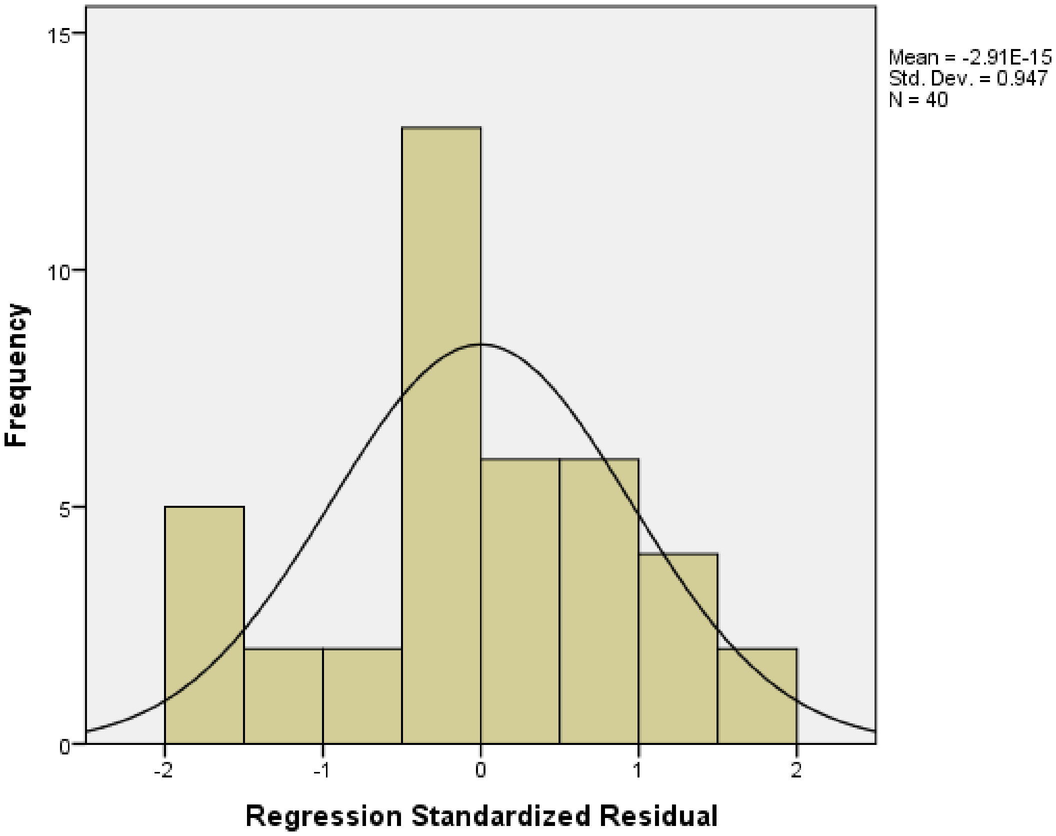


**Histogram of Standardized Residuals**

The dependent variable is the mean of pain intensity ratings.

**Figure S56.**

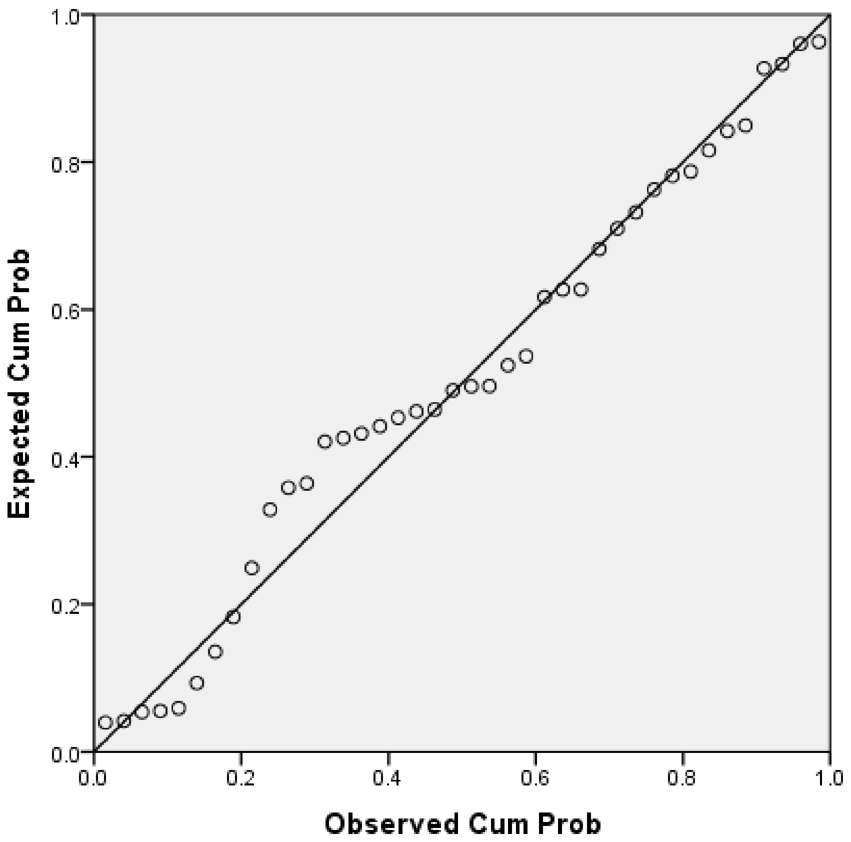


**Normal P-P Plot of Regression Standardized Residual.**
The dependent variable is the mean of pain intensity ratings.

**Figure S57.**

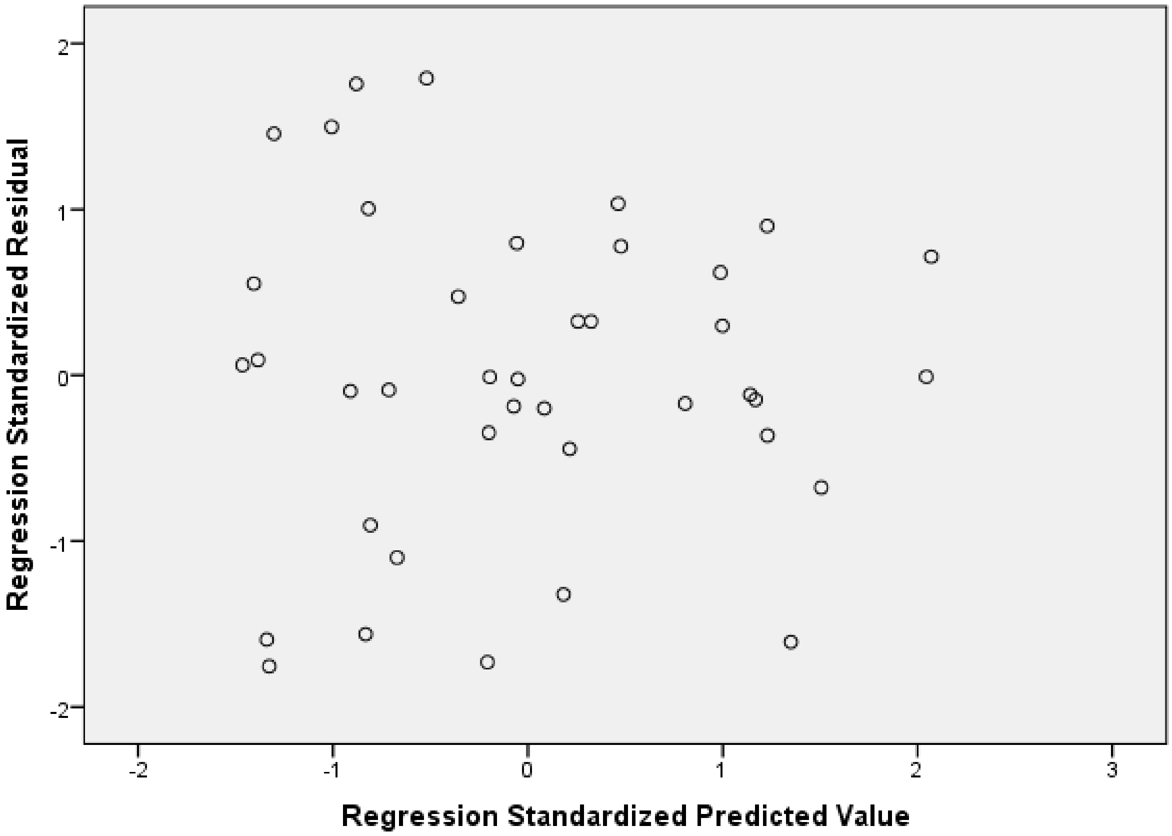


**Scatterplot.**
The dependent variable is the mean of pain intensity ratings.

Table S23. The Tolerance and the Variance Inflation Factor for Each Predictor Variable.

| | | Collinearity Statistics | |
|---|---|---|---|
| Steps | Variables | Tolerance | VIF |
| 1 | Occipital β | 0.975 | 1.026 |
| | Trait Mindfulness | 0.975 | 1.026 |
| 2 | Occipital β | 0.878 | 1.139 |
| | Trait Mindfulness | 0.816 | 1.225 |
| | Sex | 0.791 | 1.265 |
| 3 | Occipital β | 0.373 | 2.679 |
| | Trait Mindfulness | 0.813 | 1.230 |
| | Sex | 0.788 | 1.269 |
| | Occipital βXTrait Mindfulness | 0.382 | 2.615 |

The dependent variable is the mean of pain intensity ratings. All continuous independent variables were centered (original values minus mean) before being entered into the models.

*2.20 Model 20.*

As shown in Figure S58 and Figure S59, the normality of residuals was evaluated visually using a histogram of standardized residuals and a probability-probability plot (P-P plot). Both plots indicated that the residuals approximately followed a normal distribution.

As shown in Figure S60, variance homogeneity was examined through a scatterplot of standardized residuals against predicted values. The residual plot showed no discernible pattern, suggesting constant variance across levels of the predictor variables.

Autocorrelation was assessed using the Durbin-Watson statistic, with values = 1.822, near 2, indicating no significant first-order autocorrelation.

As shown in Table S24, multicollinearity was evaluated by calculating both the tolerance and the variance inflation factor (VIF) for each predictor variable. All tolerance values were above 0.1, and all VIF values were well below the threshold of 10, indicating a low risk of multicollinearity.

A casewise diagnostic was also performed, identifying observations with standardized residuals exceeding three standard deviations to detect influential outliers; no such cases were found, suggesting the model is robust and not unduly affected by extreme values. Together, these diagnostics confirm that the model satisfies the fundamental assumptions, supporting the reliability of the regression estimates.

**Figure S58.**

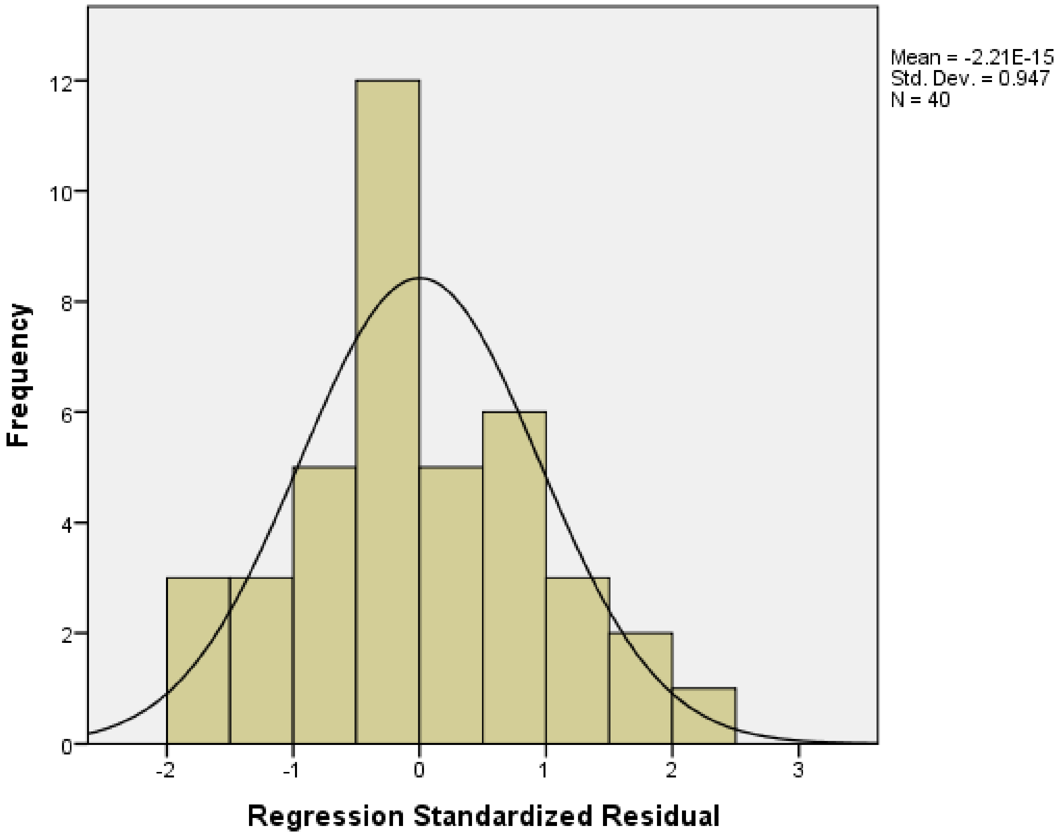


**Histogram of Standardized Residuals**

The dependent variable is the mean of pain intensity ratings.

**Figure S59.**

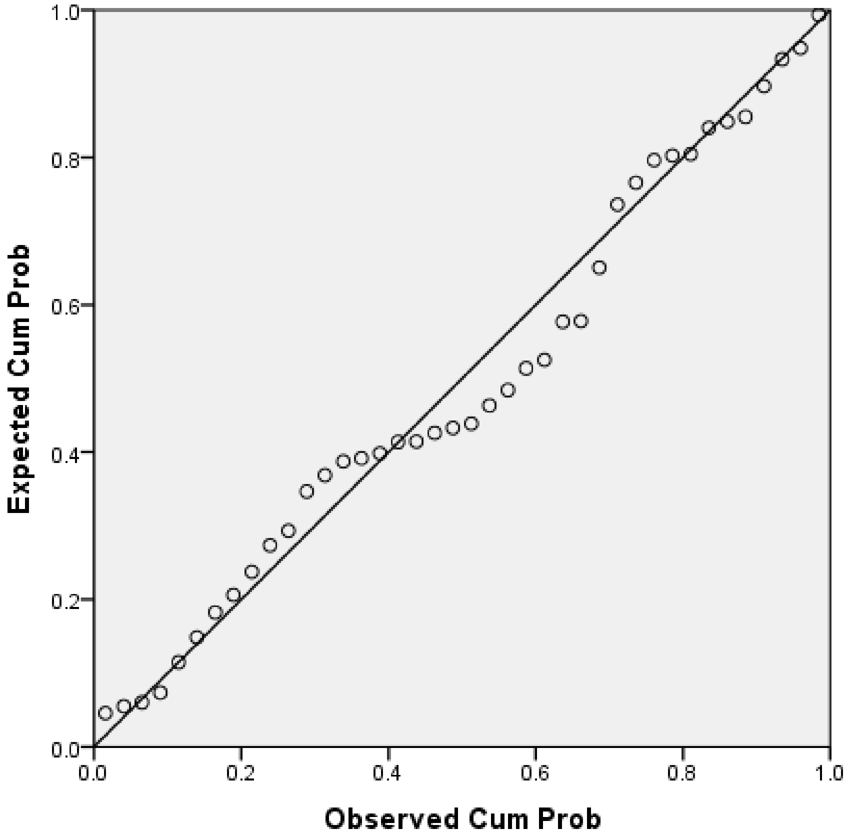


**Normal P-P Plot of Regression Standardized Residual.**
The dependent variable is the mean of pain intensity ratings.

**Figure S60.**

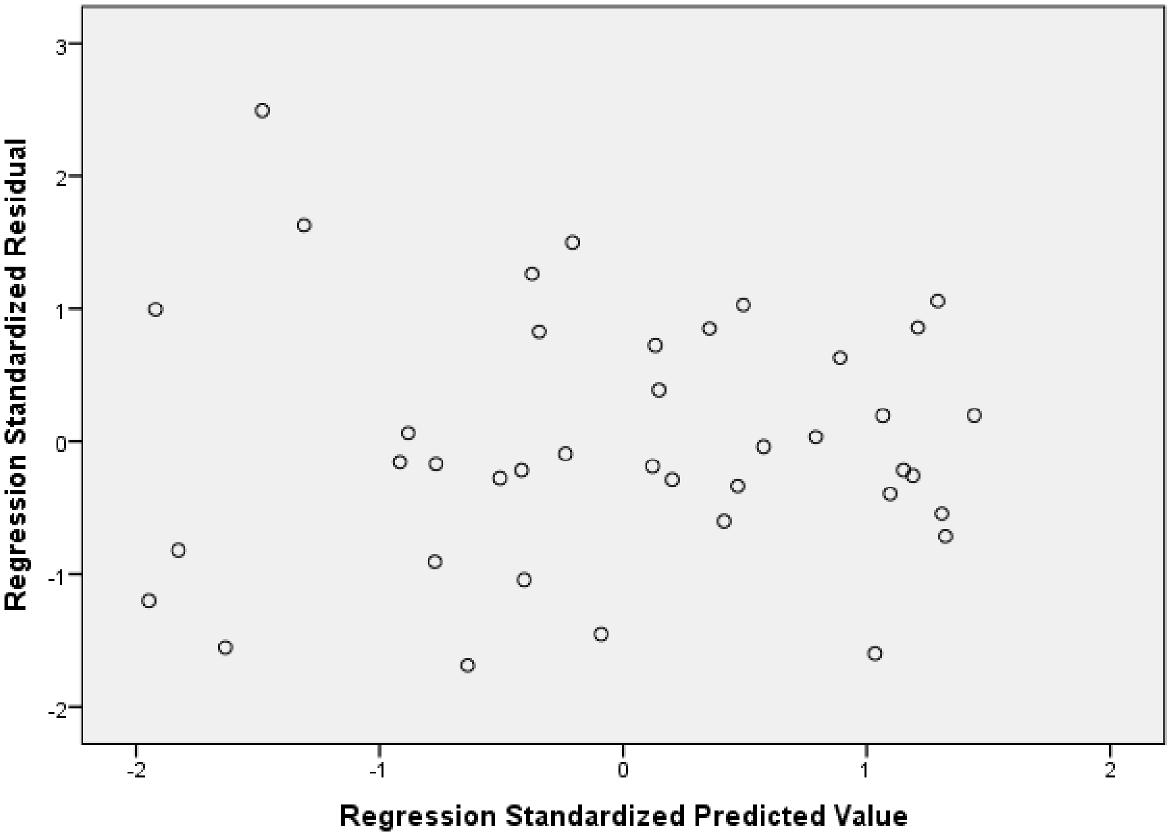


**Scatterplot.**
The dependent variable is the mean of pain intensity ratings.

Table S24. The Tolerance and the Variance Inflation Factor for Each Predictor Variable.

| Steps | Variables | Collinearity Statistics | |
|---|---|---|---|
| | | Tolerance | VIF |
| 1 | Temporal β | 0.968 | 1.033 |
| | Trait Mindfulness | 0.968 | 1.033 |
| 2 | Temporal β | 0.925 | 1.082 |
| | Trait Mindfulness | 0.826 | 1.211 |
| | Sex | 0.838 | 1.193 |
| 3 | Temporal β | 0.811 | 1.234 |
| | Trait Mindfulness | 0.728 | 1.373 |
| | Sex | 0.790 | 1.266 |
| | Temporal βXTrait Mindfulness | 0.717 | 1.396 |

The dependent variable is the mean of pain intensity ratings. All continuous independent variables were centered (original values minus mean) before being entered into the models.

*2.21 Model 21.*

As shown in Figure S61 and Figure S62, the normality of residuals was evaluated visually using a histogram of standardized residuals and a probability-probability plot (P-P plot). Both plots indicated that the residuals approximately followed a normal distribution.

As shown in Figure S63, variance homogeneity was examined through a scatterplot of standardized residuals against predicted values. The residual plot showed no discernible pattern, suggesting constant variance across levels of the predictor variables.

Autocorrelation was assessed using the Durbin-Watson statistic, with values = 2.089, near 2, indicating no significant first-order autocorrelation.

As shown in Table S25, multicollinearity was evaluated by calculating both the tolerance and the variance inflation factor (VIF) for each predictor variable. All tolerance values were above 0.1, and all VIF values were well below the threshold of 10, indicating a low risk of multicollinearity.

A casewise diagnostic was also performed, identifying observations with standardized residuals exceeding three standard deviations to detect influential outliers; no such cases were found, suggesting the model is robust and not unduly affected by extreme values. Together, these diagnostics confirm that the model satisfies the fundamental assumptions, supporting the reliability of the regression estimates.

**Figure S61.**

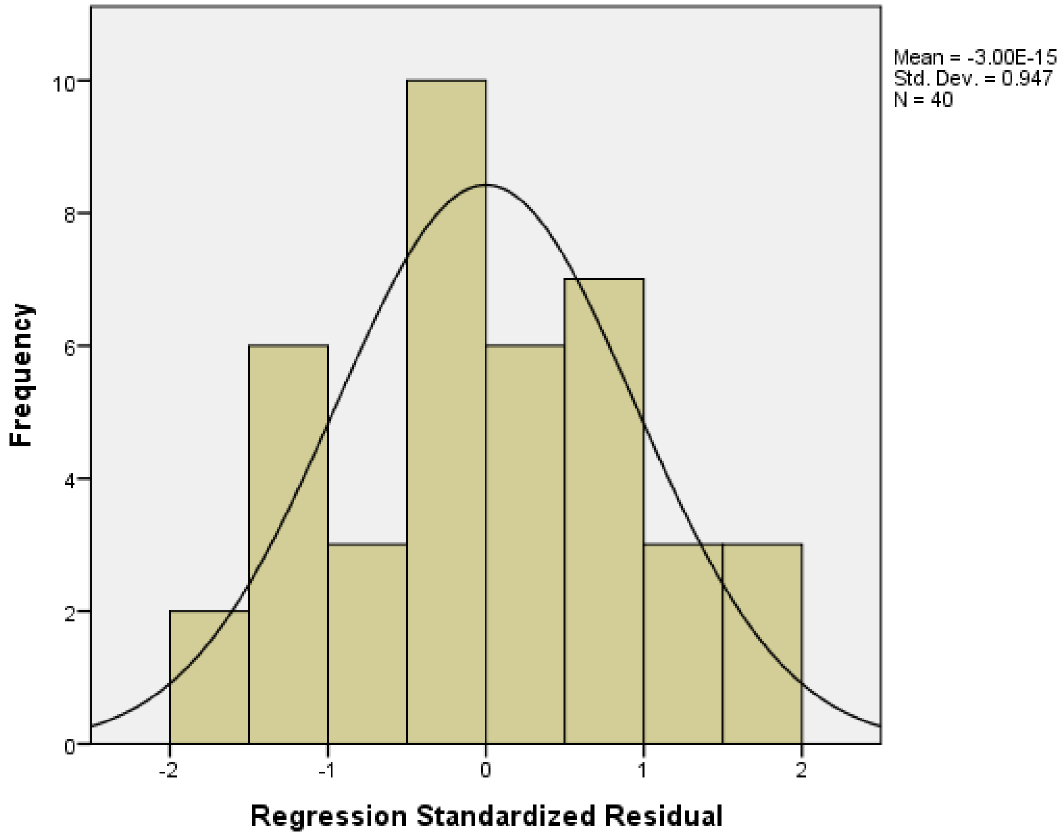


**Histogram of Standardized Residuals**

The dependent variable is the mean of pain intensity ratings.

**Figure S62.**

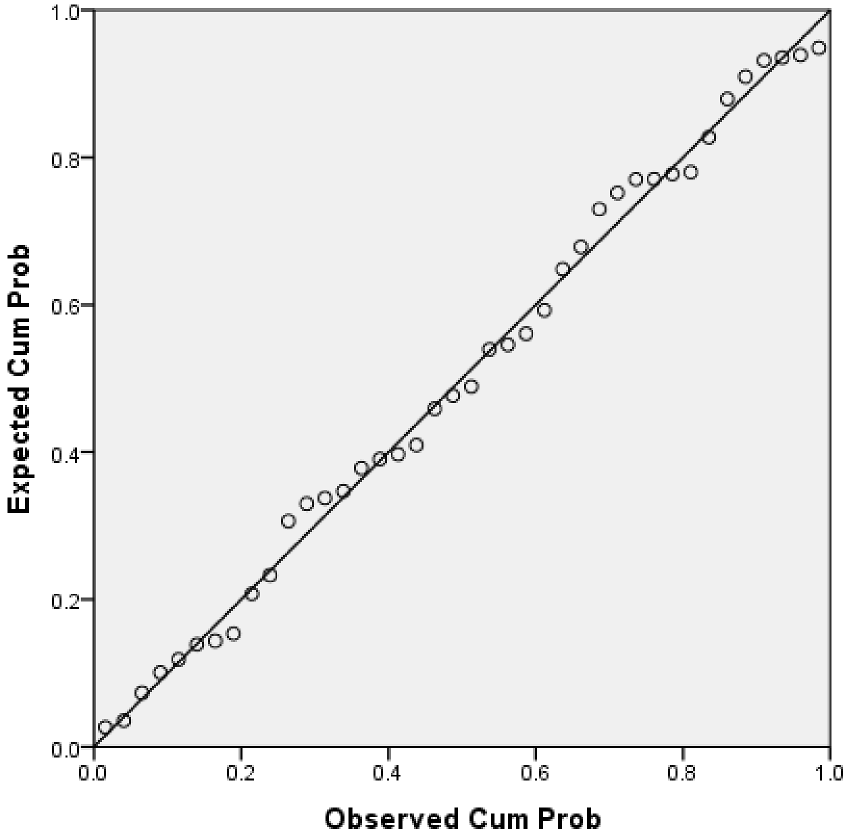


**Normal P-P Plot of Regression Standardized Residual.**
The dependent variable is the mean of pain intensity ratings.

**Figure S63.**

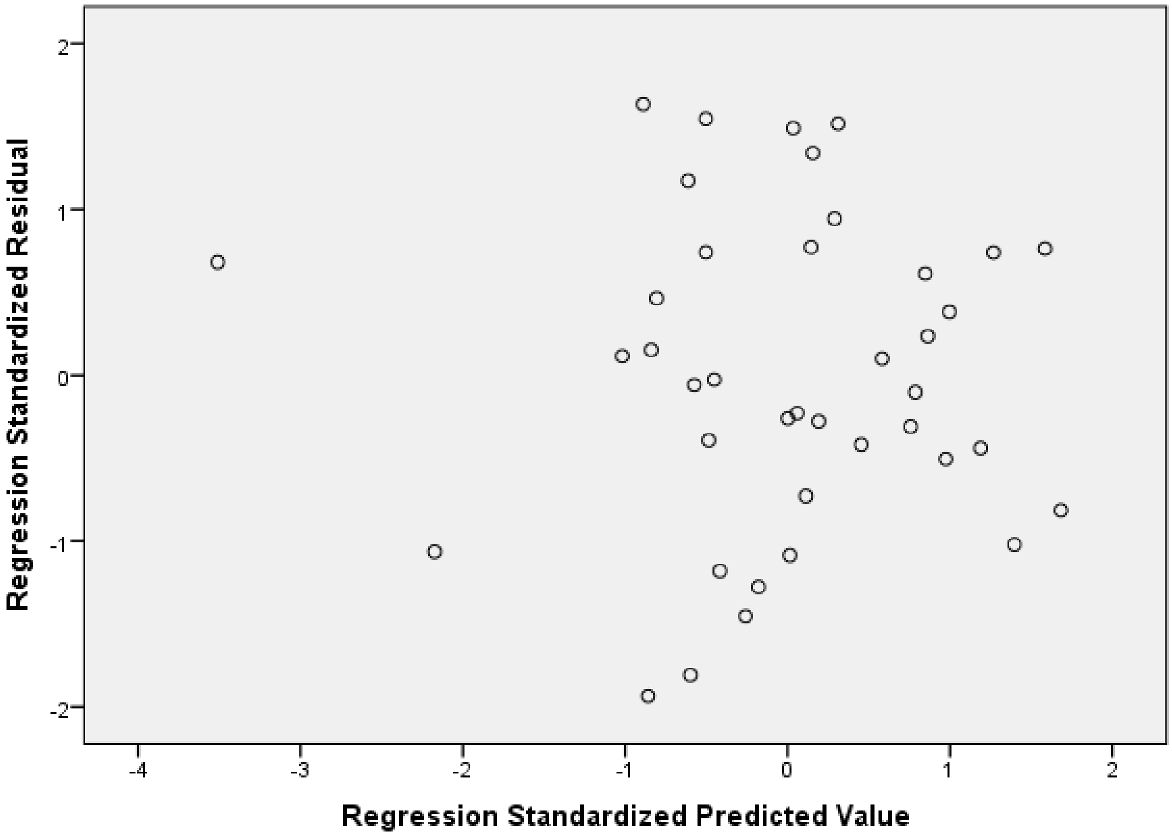


**Scatterplot.**
The dependent variable is the mean of pain intensity ratings.

Table S25. The Tolerance and the Variance Inflation Factor for Each Predictor Variable.

| | | Collinearity Statistics | |
|---|---|---|---|
| Steps | Variables | Tolerance | VIF |
| 1 | Frontal γ | 0.955 | 1.047 |
| | Trait Mindfulness | 0.955 | 1.047 |
| 2 | Frontal γ | 0.885 | 1.130 |
| | Trait Mindfulness | 0.867 | 1.154 |
| | Sex | 0.813 | 1.229 |
| 3 | Frontal γ | 0.611 | 1.637 |
| | Trait Mindfulness | 0.867 | 1.154 |
| | Sex | 0.760 | 1.316 |
| | Frontal γXTrait Mindfulness | 0.685 | 1.459 |

The dependent variable is the mean of pain intensity ratings. All continuous independent variables were centered (original values minus mean) before being entered into the models.

*2.22 Model 22.*

As shown in Figure S64 and Figure S65, the normality of residuals was evaluated visually using a histogram of standardized residuals and a probability-probability plot (P-P plot). Both plots indicated that the residuals approximately followed a normal distribution.

As shown in Figure S66, variance homogeneity was examined through a scatterplot of standardized residuals against predicted values. The residual plot showed no discernible pattern, suggesting constant variance across levels of the predictor variables.

Autocorrelation was assessed using the Durbin-Watson statistic, with values = 1.811, near 2, indicating no significant first-order autocorrelation.

As shown in Table S26, multicollinearity was evaluated by calculating both the tolerance and the variance inflation factor (VIF) for each predictor variable. All tolerance values were above 0.1, and all VIF values were well below the threshold of 10, indicating a low risk of multicollinearity.

A casewise diagnostic was also performed, identifying observations with standardized residuals exceeding three standard deviations to detect influential outliers; no such cases were found, suggesting the model is robust and not unduly affected by extreme values. Together, these diagnostics confirm that the model satisfies the fundamental assumptions, supporting the reliability of the regression estimates.

**Figure S64.**

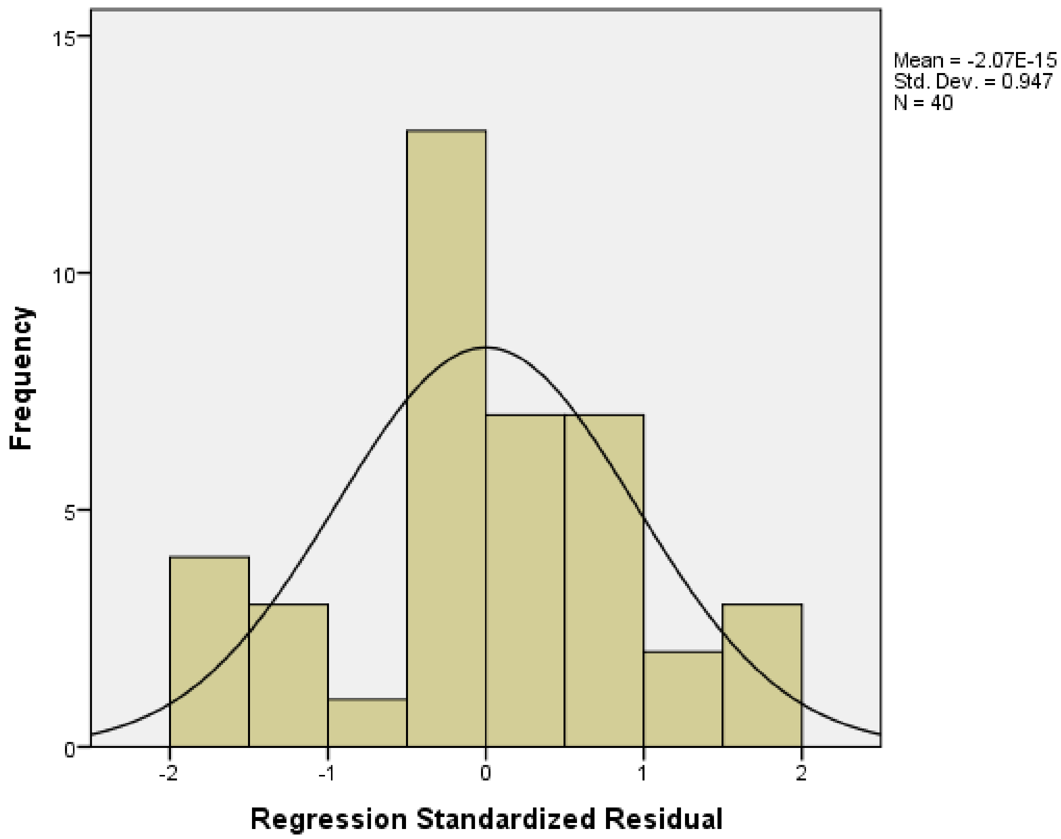


**Histogram of Standardized Residuals**

The dependent variable is the mean of pain intensity ratings.

**Figure S65.**

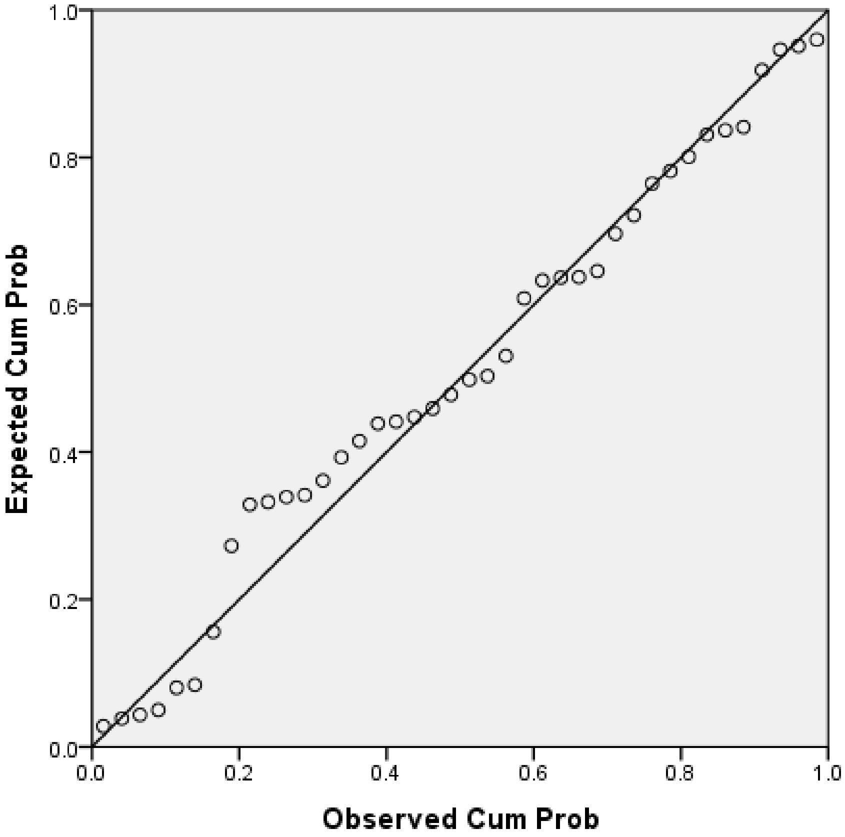


**Normal P-P Plot of Regression Standardized Residual.**
The dependent variable is the mean of pain intensity ratings.

**Figure S66.**

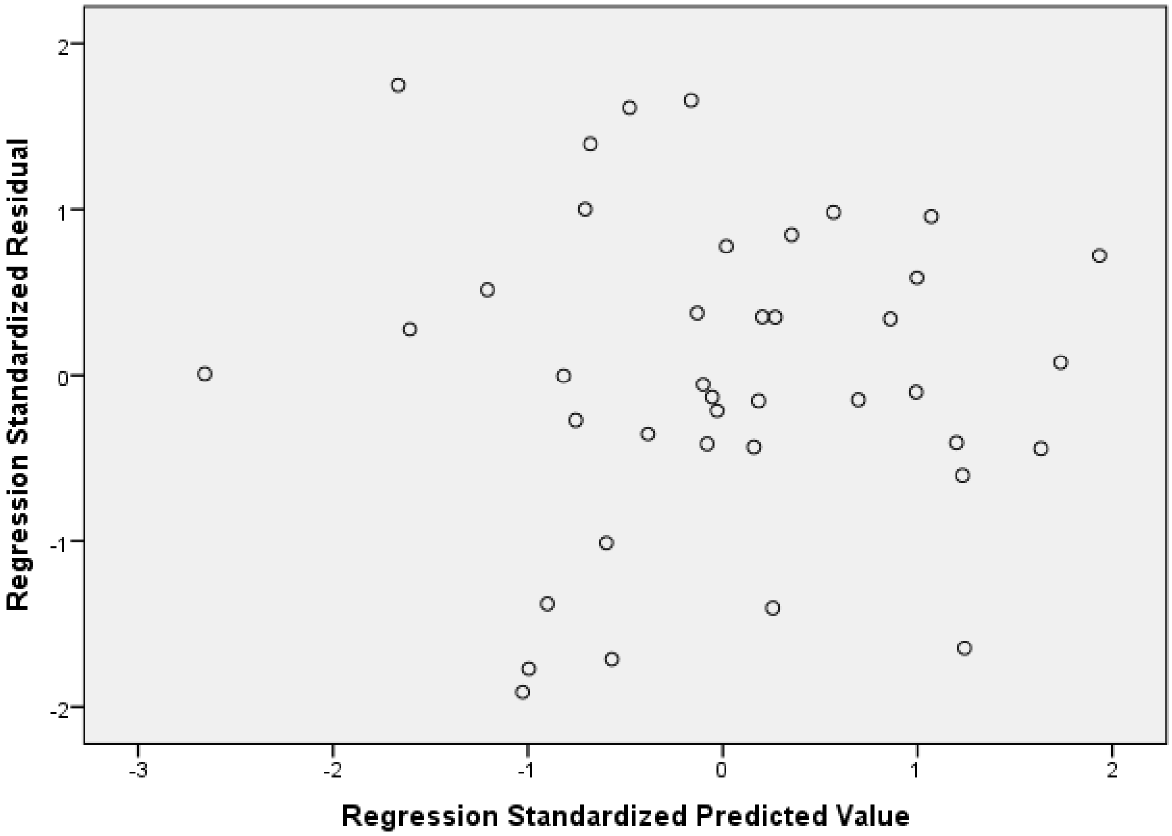


**Scatterplot.**
The dependent variable is the mean of pain intensity ratings.

Table S26. The Tolerance and the Variance Inflation Factor for Each Predictor Variable.

| | | Collinearity Statistics | |
|---|---|---|---|
| Steps | Variables | Tolerance | VIF |
| 1 | Central γ | 1.000 | 1.000 |
| | Trait Mindfulness | 1.000 | 1.000 |
| 2 | Central γ | 0.985 | 1.015 |
| | Trait Mindfulness | 0.876 | 1.142 |
| | Sex | 0.865 | 1.156 |
| 3 | Central γ | 0.950 | 1.053 |
| | Trait Mindfulness | 0.838 | 1.193 |
| | Sex | 0.865 | 1.157 |
| | Central γXTrait Mindfulness | 0.916 | 1.092 |

The dependent variable is the mean of pain intensity ratings. All continuous independent variables were centered (original values minus mean) before being entered into the models.

*2.23 Model 23.*

As shown in Figure S67 and Figure S68, the normality of residuals was evaluated visually using a histogram of standardized residuals and a probability-probability plot (P-P plot). Both plots indicated that the residuals approximately followed a normal distribution.

As shown in Figure S69, variance homogeneity was examined through a scatterplot of standardized residuals against predicted values. The residual plot showed no discernible pattern, suggesting constant variance across levels of the predictor variables.

Autocorrelation was assessed using the Durbin-Watson statistic, with values = 1.720, near 2, indicating no significant first-order autocorrelation.

As shown in Table S27, multicollinearity was evaluated by calculating both the tolerance and the variance inflation factor (VIF) for each predictor variable. All tolerance values were above 0.1, and all VIF values were well below the threshold of 10, indicating a low risk of multicollinearity.

A casewise diagnostic was also performed, identifying observations with standardized residuals exceeding three standard deviations to detect influential outliers; no such cases were found, suggesting the model is robust and not unduly affected by extreme values. Together, these diagnostics confirm that the model satisfies the fundamental assumptions, supporting the reliability of the regression estimates.

**Figure S67.**

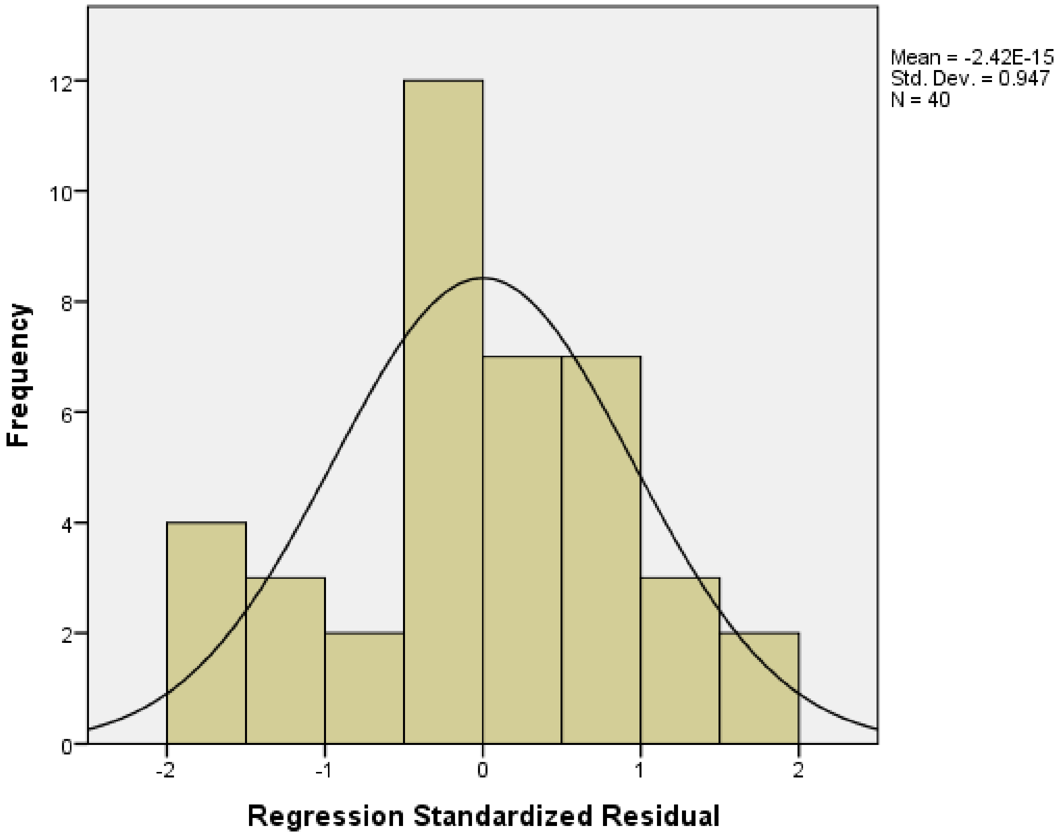


**Histogram of Standardized Residuals**

The dependent variable is the mean of pain intensity ratings.

**Figure S68.**

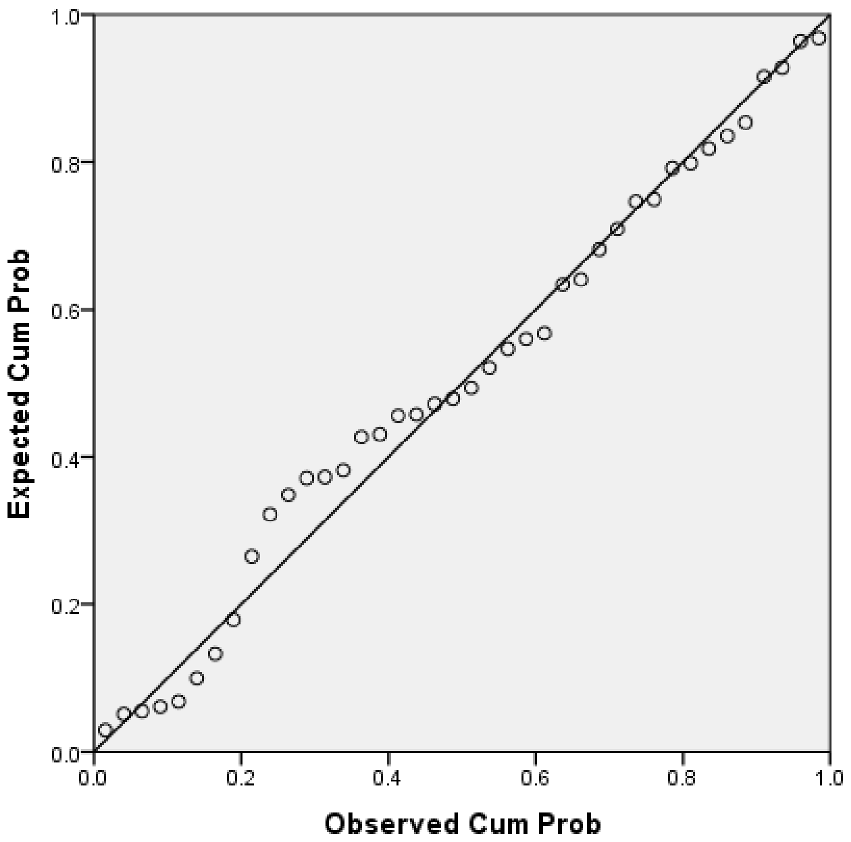


**Normal P-P Plot of Regression Standardized Residual.**
The dependent variable is the mean of pain intensity ratings.

**Figure S69.**

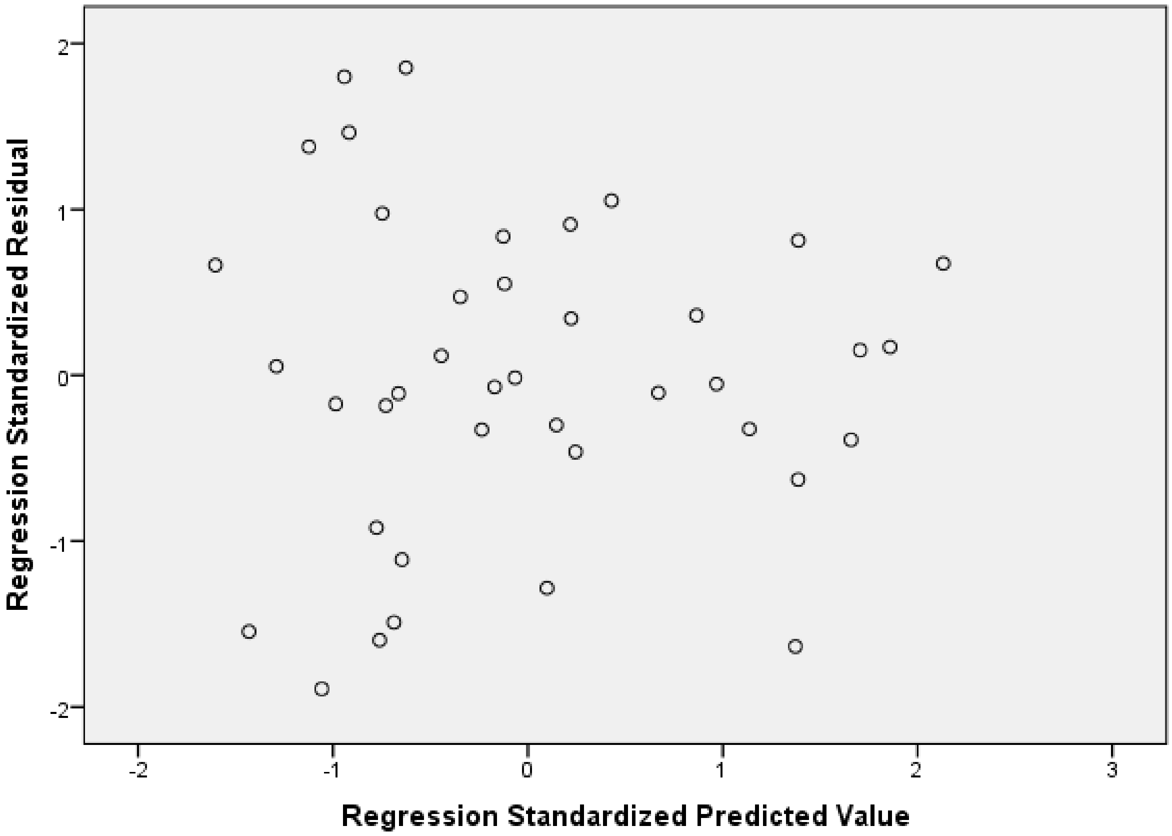


**Scatterplot.**
The dependent variable is the mean of pain intensity ratings.

Table S27. The Tolerance and the Variance Inflation Factor for Each Predictor Variable.

| | | Collinearity Statistics | |
|---|---|---|---|
| Steps | Variables | Tolerance | VIF |
| 1 | Parietal γ | 0.999 | 1.001 |
| | Trait Mindfulness | 0.999 | 1.001 |
| 2 | Parietal γ | 0.951 | 1.052 |
| | Trait Mindfulness | 0.867 | 1.154 |
| | Sex | 0.836 | 1.196 |
| 3 | Parietal γ | 0.779 | 1.284 |
| | Trait Mindfulness | 0.760 | 1.316 |
| | Sex | 0.830 | 1.206 |
| | Parietal γXTrait Mindfulness | 0.697 | 1.434 |

The dependent variable is the mean of pain intensity ratings. All continuous independent variables were centered (original values minus mean) before being entered into the models.

*2.24 Model 24.*

As shown in Figure S70 and Figure S71, the normality of residuals was evaluated visually using a histogram of standardized residuals and a probability-probability plot (P-P plot). Both plots indicated that the residuals approximately followed a normal distribution.

As shown in Figure S72, variance homogeneity was examined through a scatterplot of standardized residuals against predicted values. The residual plot showed no discernible pattern, suggesting constant variance across levels of the predictor variables.

Autocorrelation was assessed using the Durbin-Watson statistic, with values = 1.739, near 2, indicating no significant first-order autocorrelation.

As shown in Table S28, multicollinearity was evaluated by calculating both the tolerance and the variance inflation factor (VIF) for each predictor variable. All tolerance values were above 0.1, and all VIF values were well below the threshold of 10, indicating a low risk of multicollinearity.

A casewise diagnostic was also performed, identifying observations with standardized residuals exceeding three standard deviations to detect influential outliers; no such cases were found, suggesting the model is robust and not unduly affected by extreme values. Together, these diagnostics confirm that the model satisfies the fundamental assumptions, supporting the reliability of the regression estimates.

**Figure S70.**

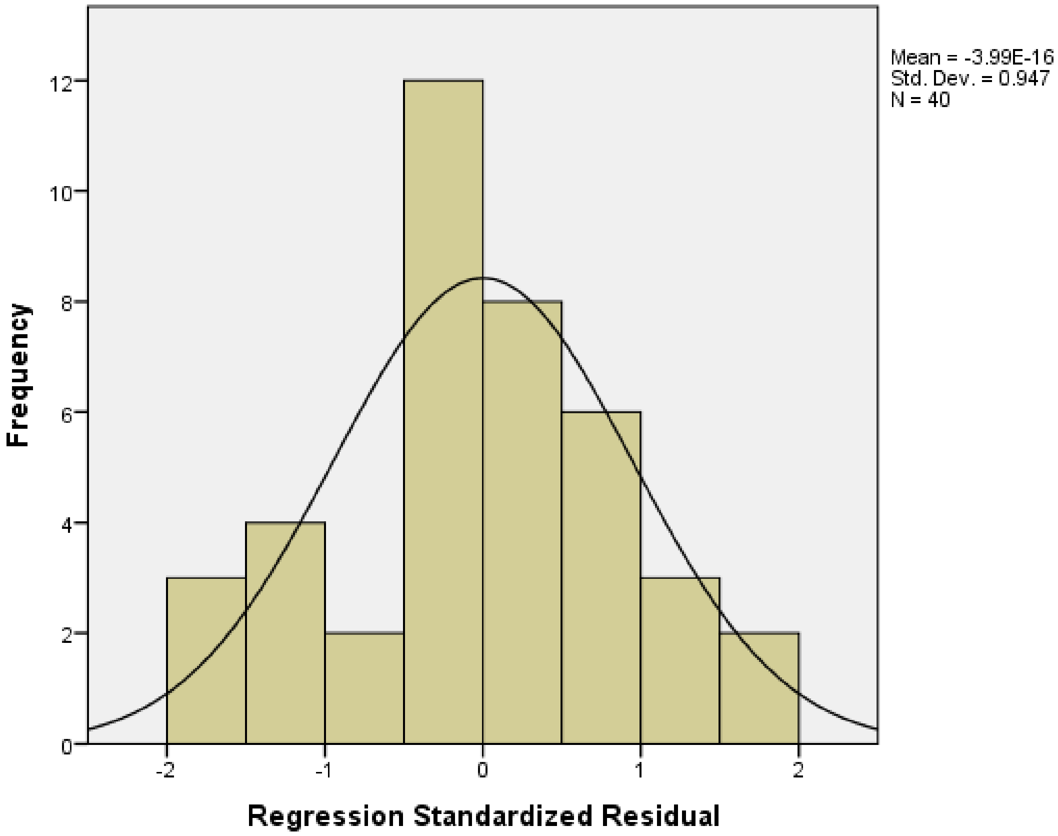


**Histogram of Standardized Residuals**

The dependent variable is the mean of pain intensity ratings.

**Figure S71.**

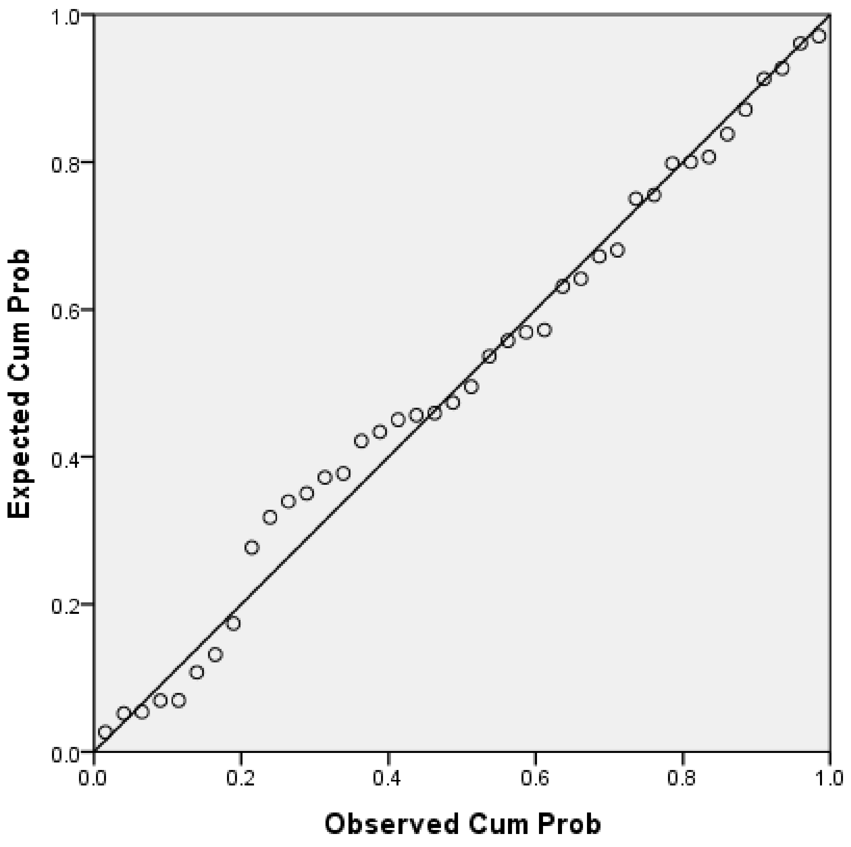


**Normal P-P Plot of Regression Standardized Residual.**
The dependent variable is the mean of pain intensity ratings.

**Figure S72.**

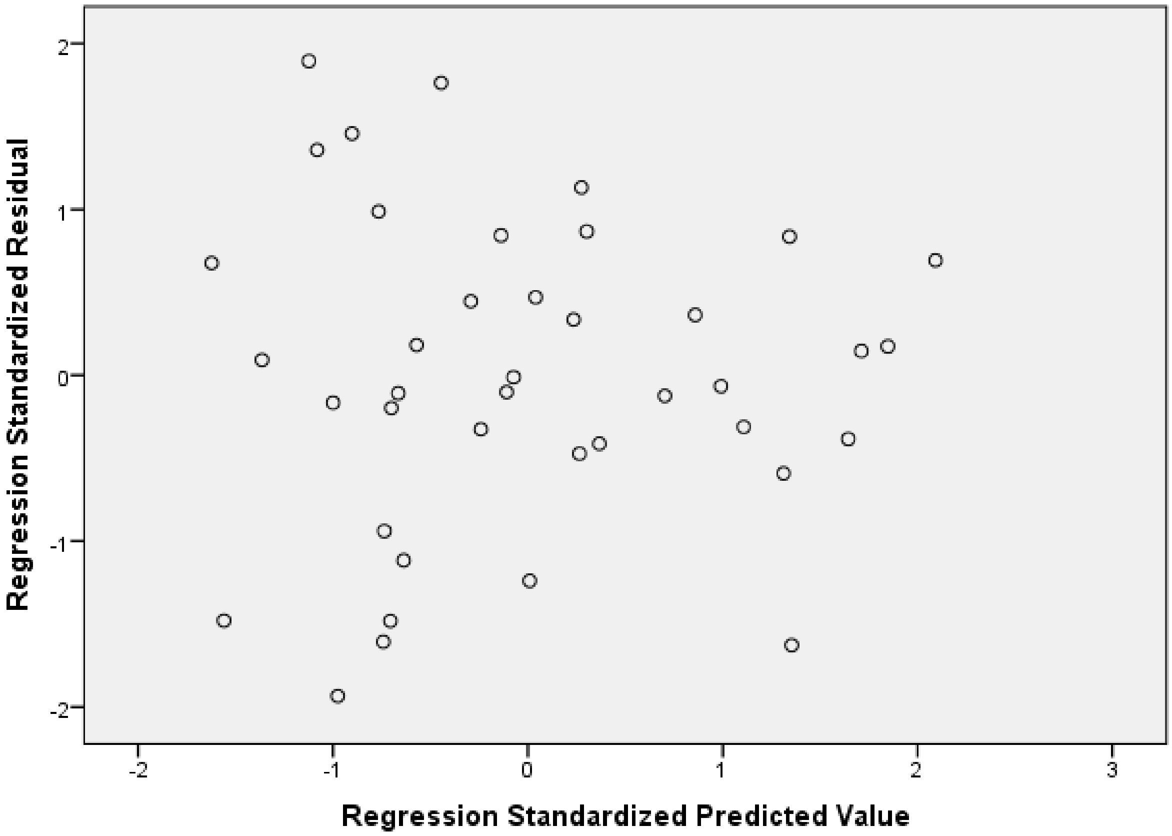


**Scatterplot.**
The dependent variable is the mean of pain intensity ratings.

Table S28. The Tolerance and the Variance Inflation Factor for Each Predictor Variable.

| Steps | Variables | Collinearity Statistics | |
|---|---|---|---|
| | | Tolerance | VIF |
| 1 | Occipital γ | 0.995 | 1.005 |
| | Trait Mindfulness | 0.995 | 1.005 |
| 2 | Occipital γ | 0.971 | 1.029 |
| | Trait Mindfulness | 0.865 | 1.156 |
| | Sex | 0.857 | 1.167 |
| 3 | Occipital γ | 0.323 | 3.096 |
| | Trait Mindfulness | 0.672 | 1.489 |
| | Sex | 0.854 | 1.171 |
| | Occipital γXTrait Mindfulness | 0.300 | 3.335 |

The dependent variable is the mean of pain intensity ratings. All continuous independent variables were centered (original values minus mean) before being entered into the models.

*2.25 Model 25.*

As shown in Figure S73 and Figure S74, the normality of residuals was evaluated visually using a histogram of standardized residuals and a probability-probability plot (P-P plot). Both plots indicated that the residuals approximately followed a normal distribution.

As shown in Figure S75, variance homogeneity was examined through a scatterplot of standardized residuals against predicted values. The residual plot showed no discernible pattern, suggesting constant variance across levels of the predictor variables.

Autocorrelation was assessed using the Durbin-Watson statistic, with values = 1.803, near 2, indicating no significant first-order autocorrelation.

As shown in Table S29, multicollinearity was evaluated by calculating both the tolerance and the variance inflation factor (VIF) for each predictor variable. All tolerance values were above 0.1, and all VIF values were well below the threshold of 10, indicating a low risk of multicollinearity.

A casewise diagnostic was also performed, identifying observations with standardized residuals exceeding three standard deviations to detect influential outliers; no such cases were found, suggesting the model is robust and not unduly affected by extreme values. Together, these diagnostics confirm that the model satisfies the fundamental assumptions, supporting the reliability of the regression estimates.

**Figure S73.**

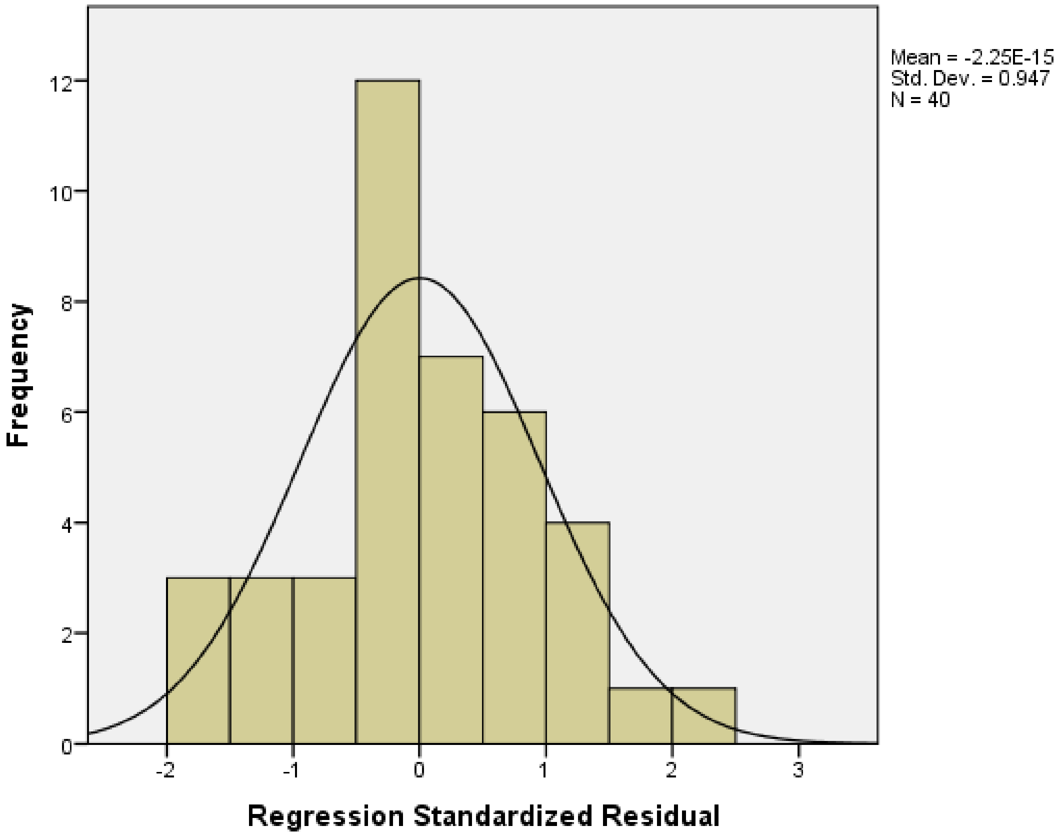


**Histogram of Standardized Residuals**

The dependent variable is the mean of pain intensity ratings.

**Figure S74.**

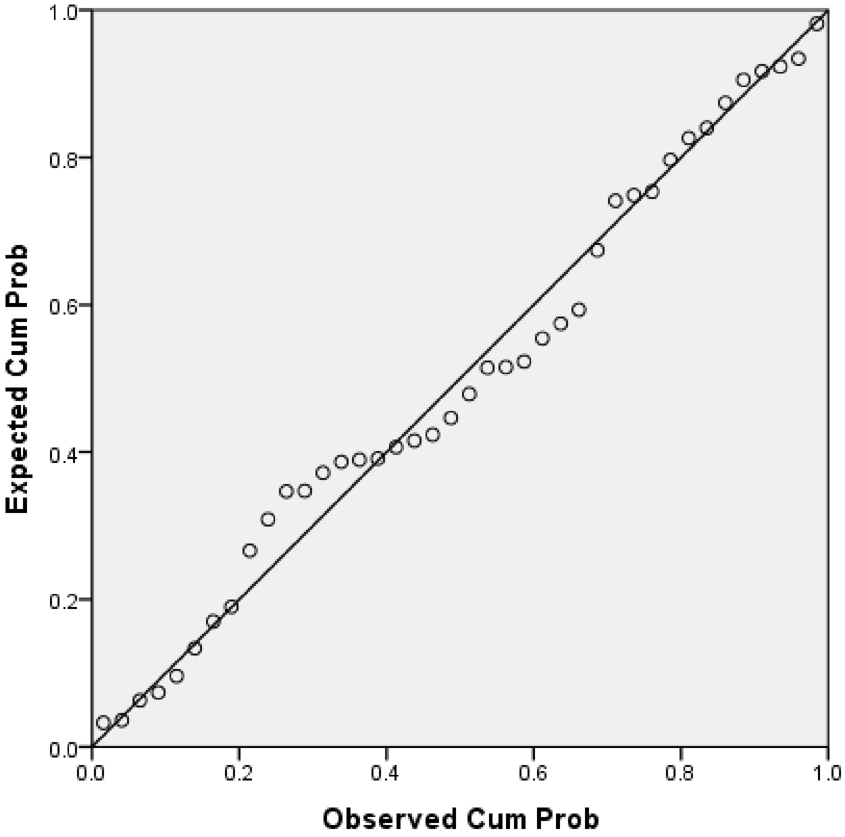


**Normal P-P Plot of Regression Standardized Residual.**
The dependent variable is the mean of pain intensity ratings.

**Figure S75.**

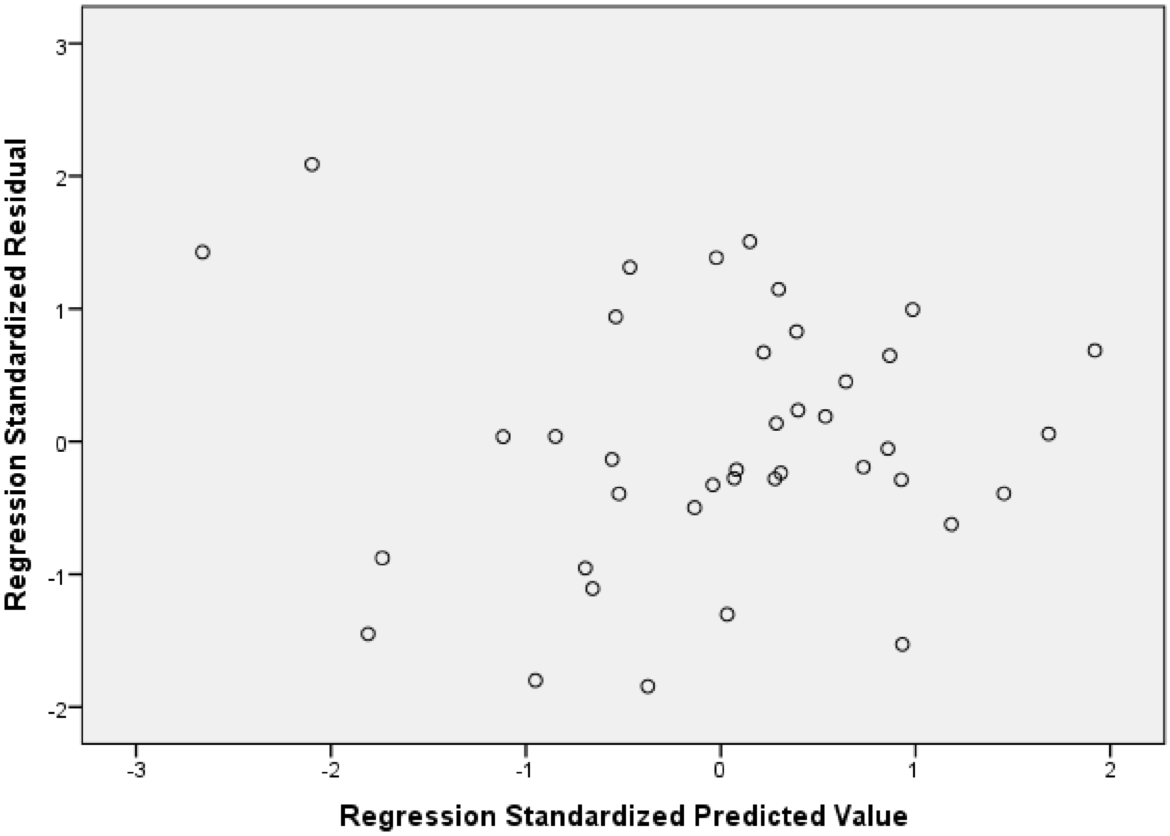


**Scatterplot.**
The dependent variable is the mean of pain intensity ratings.

Table S29. The Tolerance and the Variance Inflation Factor for Each Predictor Variable.

| | | Collinearity Statistics | |
|---|---|---|---|
| Steps | Varaibles | Tolerance | VIF |
| 1 | Temporal γ | 0.988 | 1.012 |
| | Trait Mindfulness | 0.988 | 1.012 |
| 2 | Temporal γ | 0.988 | 1.012 |
| | Trait Mindfulness | 0.867 | 1.153 |
| | Sex | 0.877 | 1.140 |
| 3 | Temporal γ | 0.919 | 1.088 |
| | Trait Mindfulness | 0.838 | 1.193 |
| | Sex | 0.830 | 1.205 |
| | Temporal γXTrait Mindfulness | 0.866 | 1.155 |

The dependent variable is the mean of pain intensity ratings. All continuous independent variables were centered (original values minus mean) before being entered into the models.
